# A Multiplex MoClo Toolkit for Extensive and Flexible Engineering of *Saccharomyces cerevisiae*

**DOI:** 10.1021/acssynbio.3c00423

**Published:** 2023-11-06

**Authors:** William M. Shaw, Ahmad S. Khalil, Tom Ellis

**Affiliations:** † Biological Design Center, Boston University, Boston, Massachusetts 02215, United States; ‡ Department of Biomedical Engineering, Boston University, Boston, Massachusetts 02215, United States; § Department of Bioengineering, Imperial College London, London SW7 2AZ, U.K.; ∥ Imperial College Centre for Synthetic Biology, Imperial College London, London SW7 2AZ, U.K.; ⊥ Wyss Institute for Biologically Inspired Engineering, Harvard University, Boston, Massachusetts 02215, United States

**Keywords:** *Saccharomyces cerevisiae*, multiplex, modular cloning, toolkit, synthetic biology, CRISPR-Cas9

## Abstract

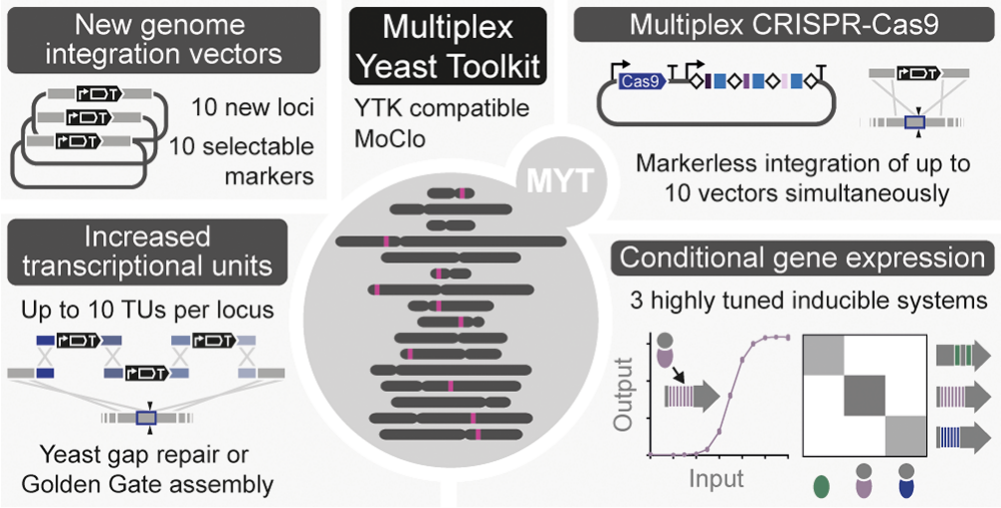

Synthetic biology toolkits are one of the core foundations on which the field has been built, facilitating and accelerating efforts to reprogram cells and organisms for diverse biotechnological applications. The yeast *Saccharomyces cerevisiae*, an important model and industrial organism, has benefited from a wide range of toolkits. In particular, the MoClo Yeast Toolkit (YTK) enables the fast and straightforward construction of multigene plasmids from a library of highly characterized parts for programming new cellular behavior in a more predictable manner. While YTK has cultivated a strong parts ecosystem and excels in plasmid construction, it is limited in the extent and flexibility with which it can create new strains of yeast. Here, we describe a new and improved toolkit, the Multiplex Yeast Toolkit (MYT), that extends the capabilities of YTK and addresses strain engineering limitations. MYT provides a set of new integration vectors and selectable markers usable across common laboratory strains, as well as additional assembly cassettes to increase the number of transcriptional units in multigene constructs, CRISPR-Cas9 tools for highly efficient multiplexed vector integration, and three orthogonal and inducible promoter systems for conditional programming of gene expression. With these tools, we provide yeast synthetic biologists with a powerful platform to take their engineering ambitions to exciting new levels.

## Introduction

Synthetic biology aims to apply engineering principles to redesign cells and organisms, thus creating biotechnologies with the potential to address pressing global issues like human health, sustainability, and climate change, as well as new tools to interrogate complex biological processes.^1^ Standardized toolkits that enable the assembly of complex genetic constructs from a set of basic parts, such as promoters, protein coding sequences, and terminators, have been instrumental in achieving these goals.^2,3^ By using well-characterized modular parts with predictable behavior, researchers can abstract the inherent complexity of biological systems and instead focus on the higher-level aspects of experimental design.^4^ This has led to faster design-build-test-learn (DBTL) cycles, improved predictability in engineered biological systems, and has simplified the sharing of new genetic designs, which is crucial for advancing the field.

As one of the main organisms used in synthetic biology, engineering of the yeast *Saccharomyces cerevisiae* has been greatly facilitated by a range of toolkits, including YeastFab,^5^ MoClo Yeast Toolkit,^4^ Yeast Golden Gate,^6^ and EasyClone-MarkerFree.^7^ Among these, the MoClo Yeast Toolkit (YTK), developed by Lee and co-workers,^4^ has gained widespread adoption due to its ease of use, flexibility, and comprehensive library of highly characterized parts.^3,8^ YTK broadly follows the Modular Cloning (MoClo) standard^9^ that allows for the hierarchical construction of multiple genes using Golden Gate cloning, which relies on Type IIS restriction enzymes that cut outside of their recognition sequence to achieve quasi-scarless assembly.^10,11^

YTK categorizes plasmids into three distinct levels: Level 0 contains a library of individual modular parts such as promoters, coding sequences, peptide tags, and terminators. These parts are assembled at Level 1 to create transcriptional units (TUs), which can then be combined at Level 2 to create multigene constructs. Each Level 0 part has unique BsaI-generated overhangs that define their order within the Level 1 cassette, and Level 2 multigene constructs are organized using unique BsmBI-generated overhangs within the connector sequences.

The main YTK system is available in plate format from Addgene (Kit #1000000061) and consists of 96 plasmids, 94 of which are standardized parts. The toolkit defines eight types of Level 0 parts: promoters (Type 2), coding sequences (Type 3), terminators (Type 4), selection markers (Type 6), yeast origins of replication (Type 7), connectors (Type 1 and 5), and plasmid backbones for cloning in *E. coli* (Type 8). Parts can be further subdivided, for example, into Types 3a and 3b if a protein tag is required. One key feature of YTK is the inclusion of well-characterized promoters and terminators that have a wide range of relative strengths, providing researchers with unprecedented flexibility for tuning and optimizing the outputs of their genetic constructs. Vectors can be integrated into the genome by including 5′ and 3′ homology arms, which reduces variability between strains and allows for flexibility of growth conditions. The toolkit also includes detailed documentation to help users design their own parts, which can be introduced into the system by PCR or DNA synthesis using a universal Level 0 entry vector.

Since its debut in 2015, YTK has empowered numerous studies^12,13^ and inspired the development of a range of new yeast toolkits based on its framework for optogenetics,^14^ polycistronic-like gene expression,^15^ G protein-coupled receptor-based sensors,^16^ CRISPRai,^17^ and most recently, an extension of the original toolkit for CRISPR applications, genomic integrations, and combinatorial libraries.^8^ YTK has simplified the construction of yeast plasmids, and the interchangeability of parts and hierarchical workflow allow for rapid iterations of the DBTL cycle. Moreover, MoClo-compatible software makes YTK automatable, enabling biofoundries to streamline the assembly process.^18,19^ However, while YTK has established a robust parts ecosystem and excels in multigene plasmid construction, it has limitations regarding the extent to which strains can be engineered in a single transformation (multiplexing capabilities) and compatibility across laboratory strains of yeast (flexibility).

Here, we introduce the Multiplex Yeast Toolkit (MYT), a new platform for yeast synthetic biologists that extends the capabilities of YTK and enables more extensive and flexible engineering. MYT comprises 96 plasmids, including 10 markerless integration vectors that target newly defined genomic loci that are highly conserved among common laboratory strains. These can be combined with 10 selectable markers for strain and application flexibility and can accommodate an expanded number of up to 10 TUs per multigene construct. Assembly can be either by Golden Gate in vitro or by gap repair in yeast, and the toolkit also includes a highly efficient and easy-to-use CRISPR-Cas9 toolkit that can markerlessly integrate all 10 integration vectors simultaneously with >60% efficiency. Finally, we also include three highly tuned and orthogonal inducible promoter systems designed to be used in concert and without the need for nutritional perturbations. Crucially, MYT is fully backward compatible with YTK, and MYT assembly cassettes are provided preassembled to simplify Level 1 assembly and make the kit immediately usable to all current YTK users.

## Results and Discussion

### Highly Characterized Set of Genome Integration Vectors

The cornerstone of MYT is a set of 10 versatile genomic integration vectors that target intergenic regions in the *S. cerevisiae* genome. While previous studies have developed and characterized integration sites in yeast, these are often not truly intergenic (i.e., found within a few hundred base pairs of an open reading frame) and they typically have low multiplexing capacity or require the preinstallation of landing pad sequences to achieve higher integration efficiencies.^4,7,20−24^

**Figure 1:**
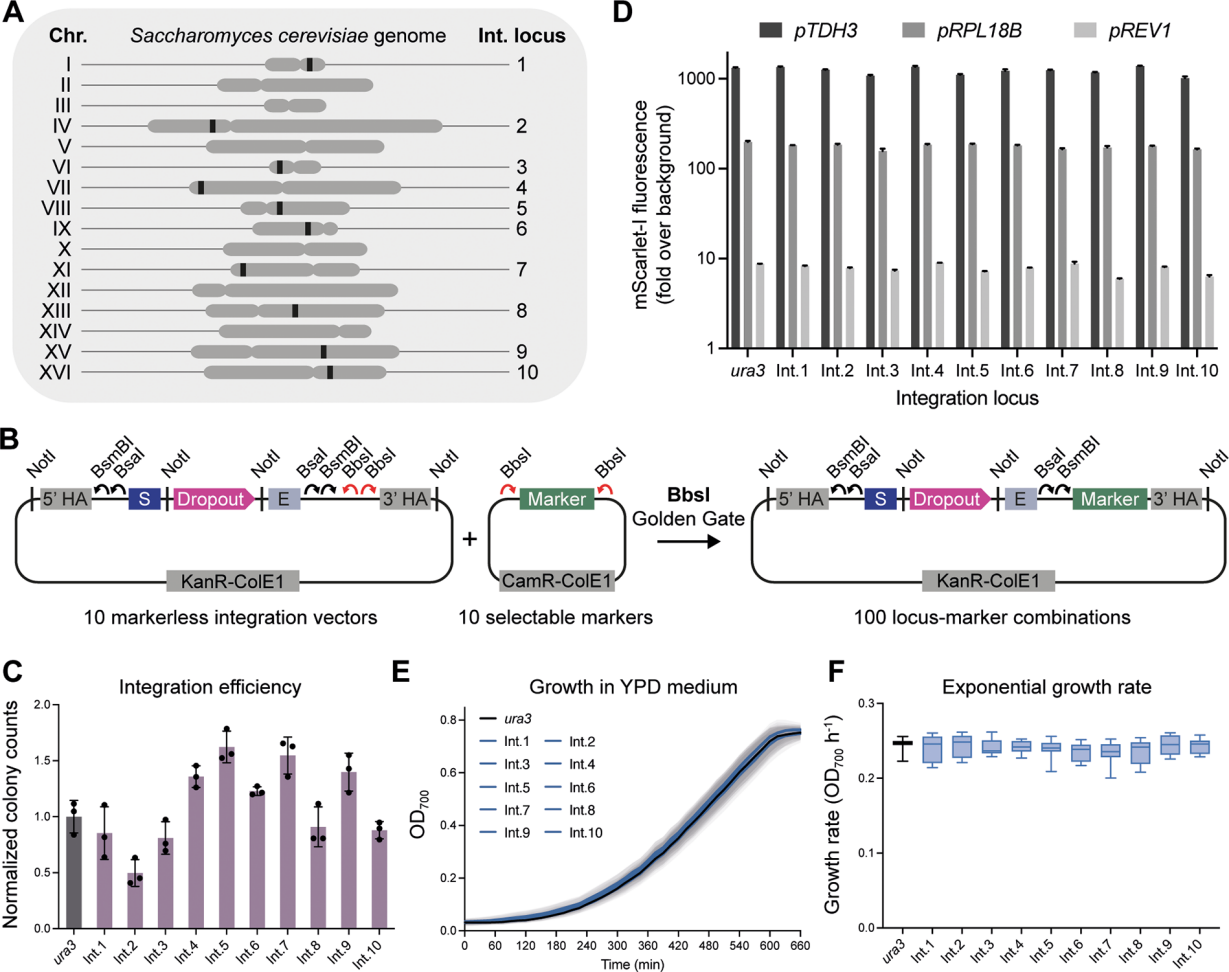
*Saccharomyces cerevisiae* genome integration vectors. (A) Chromosomal loci of the 10 integration vector landing sites across the *S. cerevisiae* genome. (B) Simplified architecture of a markerless yeast genome integration vector, highlighting the main restriction sites and demonstrating the insertion of a selection marker to generate a selectable integration vector in a BbsI Golden Gate assembly. (C) Integration efficiency of the 10 integration vectors. Experimental measurements are total colony counts normalized to the pYTK096 vector from YTK (*ura3*) and shown as the mean ± SD from three independent transformations. (D) mScarlet-I expression levels across the 10 chromosomal loci under the control of a strong (*pTDH3*), moderate (*pRPL18B*), and weak (*pREV1*) promoter from YTK, using the pYTK096 *ura3* locus as a comparison. Experimental measurements are mScarlet-I levels per cell as determined by flow cytometry and shown as the mean ± SD from three biological replicates and normalized to an untransformed control (wildtype BY4741). (E) Growth curves in YPD medium for strains containing *pREV1*-mScarlet-I-*tTDH1* at the 10 MYT integration loci and the *ura3* locus. Experimental measurements are OD_700_ over time as determined by a plate reader and shown as the mean (line) ± SD (shaded) from eight biological replicates. (F) Maximum growth rate of the strains from panel E. Data are maximum growth rates calculated from growth at the exponential phase in YPD medium and shown as mean ± SD from eight biological replicates. No statistically significant differences were observed between the maximum growth rates of all strains tested by one-way ANOVA.

To improve on these properties, we chose to identify a new set of integration loci based on a set of four guiding principles: (i) minimize the effects of plasmid integration on the host cell, (ii) maximize the chances of efficient and stable plasmid integration, (iii) be highly conserved across common laboratory stains of yeast, and (iv) be directly addressable with CRISPR-Cas9 (Supporting Table 1). Using these principles, 10 integration loci were identified across different chromosomes, all located >1 and 0.5 kb from the respective start and stop codon of any given gene (Figure 1A and Supporting Figures 1 and 2). The selection of conserved regions that can be directly targeted by CRISPR-Cas9 means that these sites do not require the preinstallation of landing pad sequences for markerless integration, and constructs can be easily ported between common laboratory strains (Supporting Table 2 for strain compatibility).

For these 10 selected genomic loci, we built 10 markerless integration vectors based on the YTK integration vector architecture (pMYT075–084), designing these for immediate use in the MYT starter kit (Figure 1B). Modifications to the architecture were made to enable yeast gap repair to be used for assembly of multigene cassettes (described further below) and to facilitate a dual mScarlet dropout marker for the red-white screening of assemblies in *E. coli* and yeast. Importantly, all MYT vectors use the same MoClo formatting as YTK and are fully compatible with any previously made Level 0 parts or Level 1 cassettes. Full details on the design and use of the integration vectors, and all other plasmids in MYT, are provided in the Supporting Text.

To add a selectable marker to an integration vector for applications when selection is preferred, an additional cloning site was introduced to the integration vectors for inserting a selectable marker using BbsI Golden Gate assembly (Figure 1B). MYT provides 10 selectable markers for use in these vectors (*URA3*, *LEU2*, *HIS3*, *TRP1*, *LYS2*, *MET17*, KanR, NatR, HygR, and ZeoR), allowing up to 100 different integration locus-marker combinations for strain and application flexibility.

To determine the integration efficiency of the individual vectors when using a selection marker, we first cloned the *URA3* gene into each integration vector followed by the assembly of a mScarlet-I TU, driven by the weak *pREV1* promoter. We then transformed 50 fmol (∼200 ng) of each NotI-digested vector into yeast, plated the cells on Synthetic Complete (SC) medium without uracil, and counted the total number of resulting colonies (Figure 1C). We observed minor variability in integration efficiency among the vectors, with colony counts ranging between 0.5× and 1.5× of those obtained using the *URA3* integration vector from YTK (pYTK096).

Expression levels at each of the 10 loci were then determined by expressing mScarlet-I from strong (*pTDH3*), moderate (*pRPL18B*), and weak (*pREV1*) YTK promoters and comparing their expression to insertion of the equivalent TUs at the *ura3* locus in BY4741 yeast, using pYTK096 (Figure 1D). The expression profiles at all 10 loci were remarkably similar, suggesting expression from these intergenic regions is highly comparable, in contrast to the large differences exhibited between different integration sites in previous toolkits.^7,21^ Similarity in expression between loci is a valuable characteristic, as genetic circuits and pathways can be expected to have similar behavior independent of their location, providing greater predictability. Differences in expression can instead be designed rationally using the large repertoire of characterized promoters available for yeast.^5,25^ However, we note that this has not yet been tested for more complex genetic constructs.

Finally, to determine if there were any fitness costs associated with integrating vectors at these sites, we picked eight colonies for each locus (*pREV1*-mScarlet-I) and performed growth curves in YPD medium (Figure 1E). Identical growth characteristics were seen between all loci, and no significant difference was observed between maximum growth rates (Figure 1F). These results suggest that there were no locus-specific effects on the fitness of the yeast, indicating that our integration vectors are not detrimental to the host cell.

### CRISPR-Cas9 for Multiplexed Markerless Vector Integration

**Figure 2:**
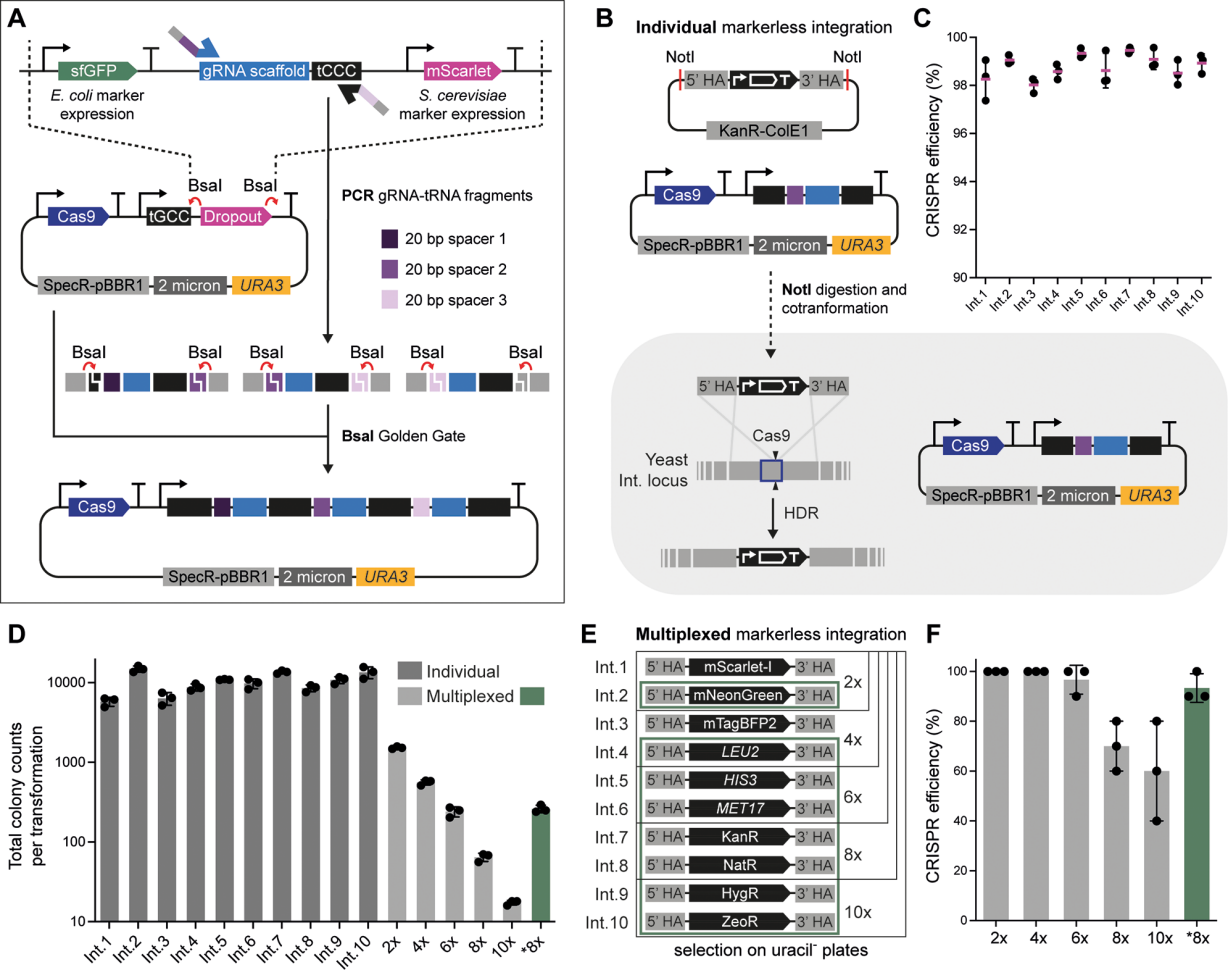
Markerless vector integration using CRISPR-Cas9. (A) Simplified CRISPR-Cas9 plasmid architecture, demonstrating the generation of gRNA-tRNA fragments by PCR and subsequent gRNA array assembly by BsaI Golden Gate cloning. (B) Schematic of markerless CRISPR-Cas9-mediated integration of a single vector containing *pTDH3*-mScarlet-I-*tTDH1*. (C) Efficiency of individual vector integration using CRISPR-Cas9. Experimental measurements are percent of transformants expressing mScarlet-I from three independent transformations, manually counted on a blue light transilluminator. (D) Total colony counts from CRISPR-Cas9 transformations, showing individual integration (dark gray), multiplexed integration (light gray), and selected 8x multiplexed integration (green). Experimental measurements are manual colony counts and shown as the mean ± SD from three independent transformations. (E) Combinations of integration vectors and reporters used in the multiplexed CRISPR-Cas9 experiments. Black boxes indicate the combinations used in the standard 2–10× conditions and the green boxes show the integration vectors used in the selected 8× condition. (F) Efficiency of multiplexed vector integration using CRISPR-Cas9. Experimental measurements are the percentage of strains validated to contain all expected reporters from 10 randomly picked colonies and shown as the mean ± SD from three independent transformations.

The second major component of MYT is a new CRISPR-Cas9 system for highly multiplexed markerless vector integration. The system is composed of a single plasmid (pMYT095) for the expression of Cas9 and a gRNA-tRNA array based on the GTR-CRISPR method from Zhang and co-workers.^26^ The arrays are generated from PCR-generated fragments that are then assembled directly into pMYT095 using a single-step BsaI Golden Gate assembly (Figure 2A and Supporting Table 3). The template DNA for creating the gRNA-tRNA fragments is located in the middle of a 3-part dropout section in the plasmid, flanked by sfGFP and mScarlet expression cassettes for green/red-white screening of assembled arrays in *E. coli* and *S. cerevisiae*, respectively. Detailed descriptions of pMYT095 and how to design and build arrays and perform CRISPR-Cas9 experiments are provided in the Supporting Text.

The pMYT095 plasmid comes with a *URA3* selection marker, which can be exchanged for any of the other nine markers in MYT using the same BbsI Golden Gate method used for inserting a marker into an integration vector. To prevent unwanted recombination between the CRISPR-Cas9 plasmid and integration vectors used during transformation, we designed pMYT095 with DNA sequences that will not be present in markerless integration vectors when using parts from YTK or MYT.

We first characterized the efficiency of the CRISPR-Cas9 system by individually integrating each of the markerless integration vectors. This was done by assembling arrays containing a single gRNA targeting each integration locus and cotransforming the digested vector and CRISPR-Cas9 plasmid into yeast (Figure 2B). Integration efficiency was determined using a *pTDH3-*mScarlet-I*-tTDH1* reporter and screening all colonies for red fluorescence (Figure 2C). For all individual targets, integration efficiencies were greater than 98%, demonstrating that our CRISPR-Cas9 system and design principles for selecting the MYT integration loci facilitate high efficiency markerless integration. We also performed total colony counts for each integration vector when using 500 fmol (∼1.5 μg) of the integration vector and 50 fmol (∼350 ng) of the CRISPR-Cas9 plasmid and measured around 10^4^ colonies for each site (Figure 2D**; dark gray**).

To determine the multiplexed CRISPR-Cas9 efficiency, we first assembled a unique reporter gene into each of the 10 markerless integration vectors, choosing genes where their successful installation into the genome could be screened using fluorescence or growth in selective media (Figure 2E). We then created CRISPR-Cas9 plasmids with corresponding gRNA arrays that target the 2, 4, 6, 8, and 10 sites simultaneously. These were then combined with the individually NotI-digested and column-purified integration vectors (Figure 2E**; black boxes**) and transformed into yeast, using the same DNA amounts as before (50 fmol CRISPR-Cas9 plasmid and 500 fmol of each integration vector). Transformants were plated on SC medium without uracil; after growth, we randomly selected 10 colonies from three independent transformation plates for each condition and assessed them by fluorescence and growth in selective media. Results showed 100% successful markerless integration in cases in which up to four vectors were used simultaneously. Despite decreasing efficiency as the multiplexing increased, simultaneous integration of all 10 vectors was observed to have a 60% success rate (Figure 2F**; light gray,**Supporting Figure 3). Precise insertion of all 10 vectors at their correct integration loci was confirmed by colony PCR of a representative strain (Supporting Figure 4).

In line with the efficiency decrease, the total colony counts for each transformation also declined as multiplexing increased. For instance, when integrating two vectors, just over 1500 colonies were obtained, whereas an average of only 17 colonies per plate were seen when all 10 vectors were used (Figure 2D**; light gray**). As vectors integrated with varying efficiencies, we selected the eight best performing integration vectors from the individual characterization data (Figure 2E**; green boxes**) and created a CRISPR-Cas9 plasmid targeting all eight loci and transformed into yeast (*8×). This combination of eight vectors showed high integration efficiency (87%) and led to a 4-fold increase in the number of colonies per plate compared to the earlier combination of eight loci (Figure 2F**; green,**Supporting Figure 5).

To discount the possibility that the reporters were contributing to the high efficiency of markerless integration, we reperformed the *8× integration using markerless vectors without any reporters and assessed their successful integration using colony PCR (Supporting Figure 6). After screening three randomly picked colonies from three independent transformations, we identified only a single colony where all eight vectors were not present. This success rate (89%) matches the previous experiments with reporters, suggesting the reporters did not introduce bias and the results represent the true markerless vector integration efficiency of the MYT CRISPR-Cas9 system.

### Transient Expression of CRISPR-Cas9 for Boosting Routine Vector Integration Efficiency

**Figure 3:**
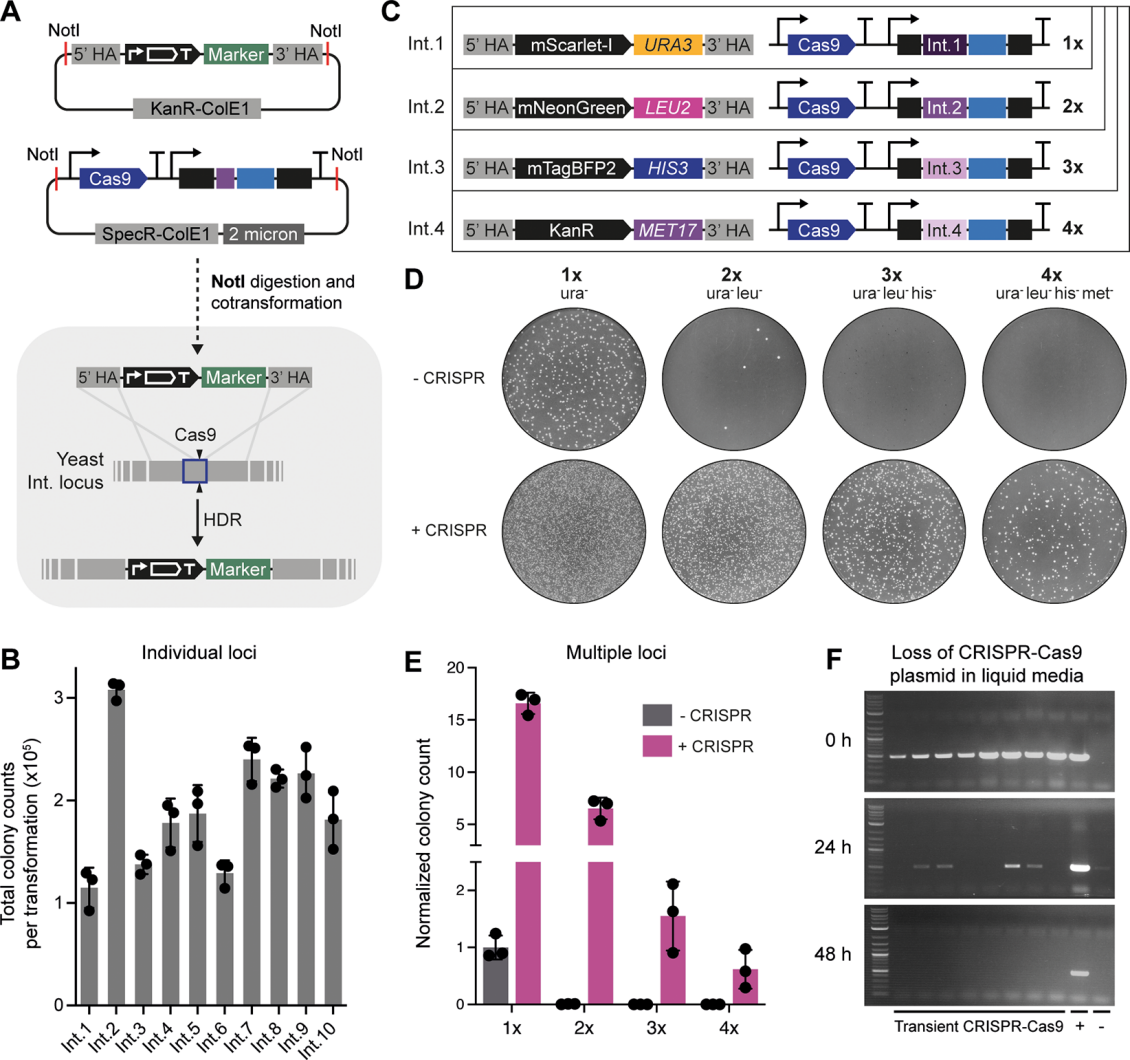
Improved integration of selectable vectors using transient CRISPR-Cas9 (A) Simplified schematic of a single vector integration using transient CRISPR-Cas9. The integration vector and transient CRISPR-Cas9 plasmid are combined in a one-pot NotI digestion and directly transformed into yeast. Transient CRISPR-Cas9 activity cuts the target locus, increasing the efficiency of integration, which is then selected using the chosen marker on the integration vector. (B) Total colony counts from the integration of individual vectors containing the *URA3* selection marker using transient CRISPR-Cas9. Experimental measurements are total colony counts and shown as the mean ± SD from three independent transformations. (C) Combinations of integration vectors, the reporters used, and the transient CRISPR-Cas9 plasmids for targeting their respective loci to assess multiplexed vector integration efficiency. Black boxes indicate the combinations used in the 1–4× conditions. (D) Transformation plates from the integration of the vectors in C, with (+ CRISPR) and without (− CRISPR) transient CRISPR-Cas9. (E) Total colony counts from multiplexed CRISPR-aided integration of the vectors in C. Experimental measurements are total colony counts normalized to the single vector integration without transient CRISPR-Cas9 (1×, – CRISPR) and shown as the mean ± SD from three independent transformations. (F) Loss of the linearized transient CRISPR-Cas9 plasmid DNA over time. PCRs were performed on 10 individual transformants from the 1×, + CRISPR condition and analyzed directly from colonies (0 h), after 24 h in liquid YPD medium (24 h) and 48 h in liquid culture following a 1:100 back dilution at 24 h (48 h). The positive control (+) is yeast containing a maintained CRISPR-Cas9 plasmid and the negative control (−) is wildtype BY4741 yeast. PCR amplicons were 500 bp of the Cas9 ORF.

As markerless integration is not always practical, we also developed an alternative method that uses transient CRISPR-Cas9 expression to boost the integration rate of vectors containing selection markers, enabling highly efficient multiplex integrations. We created a transient CRISPR-Cas9 plasmid (pMYT096) without a selection marker, designed to be linearized using NotI (Figure 3A). Individual gRNAs are generated and assembled into this plasmid using the same method as for pMYT095. However, we recommend creating individual transient CRISPR-Cas9 plasmids for each target, rather than gRNA arrays containing combinations of targets. Individual plasmids can then simply be mixed in desired combinations to target multiple loci in a single transformation. Using this alternative approach, vector integration at the 10 MYT loci were first tested separately, demonstrating colony counts above 10^6^ when using 50 fmol (∼200 ng) of the integration vector and the transient CRISPR-Cas9 plasmid (Figure 3B).

Multiplexed integration using the transient CRISPR-Cas9 system was then tested by creating four integration vectors with unique reporters and selection markers that complement the BY4741 auxotrophies (Figure 3C). Total colony counts revealed a 16.5-fold increase in transformation efficiency when using transient CRISPR-Cas9 for a single integration vector, with colony counts reducing as multiplexing increased (Figure 3D,E). As a comparison, only a few colonies were recorded for a double integration in the absence of transient CRISPR-Cas9, with no colonies reported for triple integrations or above. In contrast, a substantial number of colonies were still achievable when using transient CRISPR-Cas9 with up to four integration vectors, with numbers comparable to a single unaided integration. In all cases, the reporter output was as expected, supporting the use of selectable markers to avoid laborious genotyping (Supporting Figure 7).

Another advantage of the transient CRISPR-Cas9 method is that low quantities of DNA are required (50 fmol of each integration vector and companion transient CRISPR-Cas9 plasmid), eliminating the need for column purification. All DNA can be added to a one-pot NotI digestion and directly transformed into yeast after heat inactivation. To track the loss of the transient CRISPR-Cas9 DNA, we performed a population PCR of the Cas9 CDS from transformed colonies (0 h), and after 24 and 48 h in liquid media, with a single 1:100 back dilution at 24 h (Figure 3F). As expected, this demonstrated a reduction in Cas9 amplification after 24 h, with no detectable amounts thereafter, showing that the CRISPR-Cas9 DNA is indeed lost over time.

As the ability to complement all auxotrophies in a single transformation is an attractive prospect,^27^ integration vectors containing spacers (pMYT085–094) have been included in MYT to allow the introduction of markers without transcriptional units (see Supporting Text). This provides a rapid method to reverse autotrophies for growth in minimal medium or when there is a need to ensure auxotrophic markers are consistent between strains, for example, in growth assays. The designed spacer sequences also contain a unique CRISPR-Cas9 targeting sequence, so they can themselves be easily targeted for further downstream insertions.

### Assembly of Large Multigene Constructs In Yeast and In Vitro

One of the major draws of YTK is the ability to build complex multigene constructs from a library of well-characterized parts in just two straightforward rounds of cloning.^3,4^ However, the maximum number of TUs has been limited to six, hindering more extensive synthetic circuit design. Additionally, the Level 1 assembly requires eight or more parts, which can be inefficient and challenging to clone. To address these issues, we have increased the maximum number of TUs to 10, with each position within a multigene construct supported by a prebuilt assembly cassette, thus reducing the number of parts required in a standard Level 1 assembly by half.

**Figure 4:**
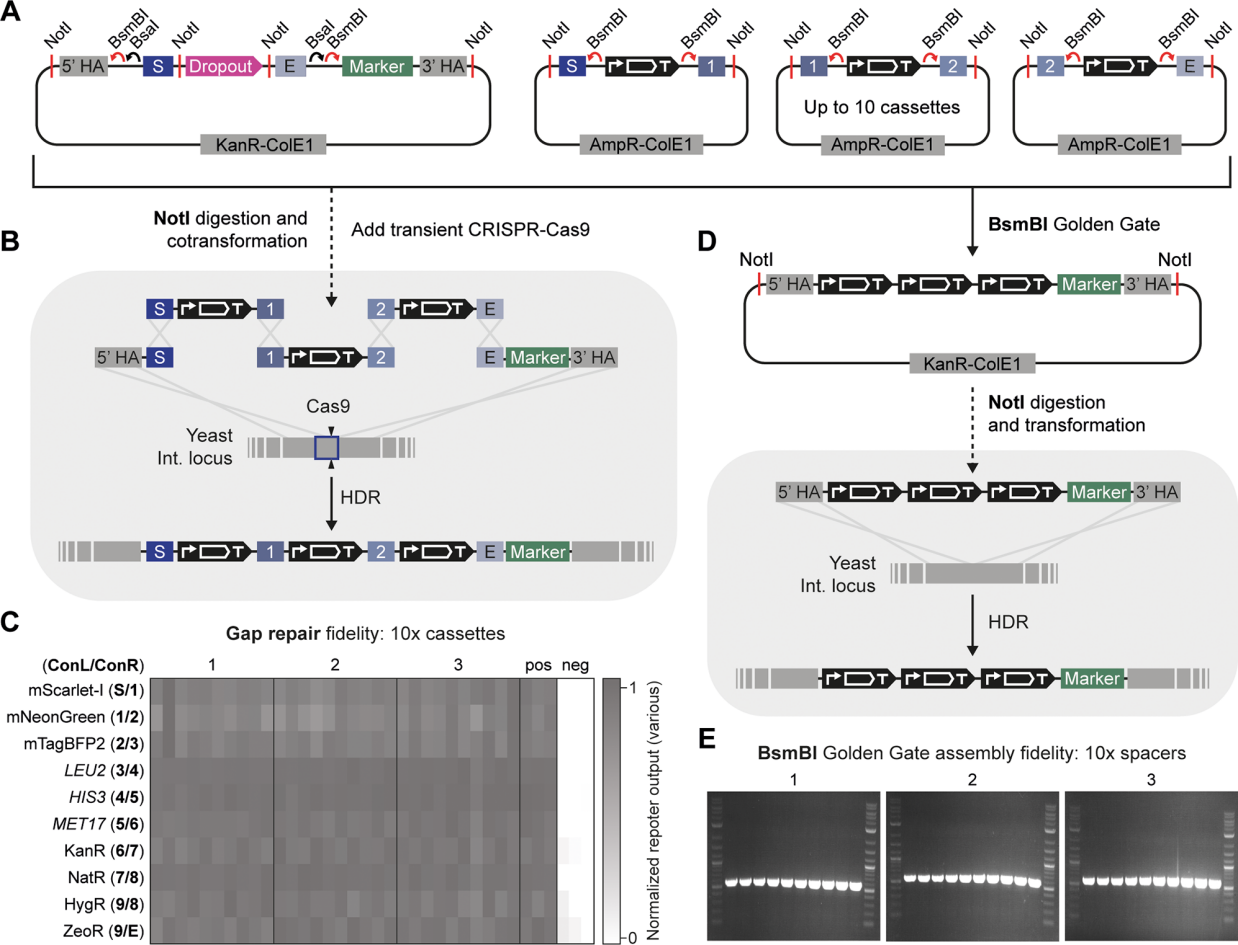
Yeast gap repair and Golden Gate assembly of up to 10 transcriptional units. (A) Simplified architecture of an unassembled integration vector and three Level 1 cassette plasmids with assembled transcriptional units, highlighting the NotI sites used for the gap repair assembly in yeast and BsmBI sites used for the Golden Gate assembly in vitro. (B) Gap repair of Level 1 cassettes directly into the yeast genome. The integration vector, cassettes, and a transient CRISPR-Cas9 plasmid targeting the appropriate locus are digested in a one-pot reaction with NotI, and directly transformed into yeast. Gap repair in yeast then assembles the final multigene construct at the genomic locus according to the overlapping homology between the integration vector and cassettes, with the transient CRISPR-Cas9 increasing the overall efficiency of the process. (C) Fidelity of a 10× Level 1 cassette gap repair assembly into the yeast genome. Ten colonies from three independent transformations were assessed for the presence of 10 distinct reporters, showing their presence in all instances. Experimental measurements are various reporter outputs normalized to the minimum (negative) and maximum (positive) response of each reporter and shown as individual values from each biological replicate. (D) BsmBI Golden Gate assembly of Level 1 cassettes into an integration vector and subsequent transformation into yeast, following plasmid preparation and validation. (E) Fidelity of BsmBI Golden Gate assembly of 10 spacers into an integration vector. Three independent BsmBI Golden Gate assemblies were performed using an integration vector and 10 assembly spacers to assess the assembly fidelity of the designed overhangs. Ten isolates from each *E. coli* transformation were then analyzed by PCR to determine correct assembly by product size (580 bp). All colonies were correct and additionally confirmed by Sanger sequencing.

Moreover, we have modified the Level 1 cassettes to simplify using gap repair assembly in yeast, which provides users with an option to bypass the Level 2 Golden Gate assembly step altogether, shortening the time to integrate multigene plasmids by 1–2 days (Figure 4A,B). This modification was achieved by including additional NotI sites in the assembly cassettes and unassembled integration vectors, which release homology arms after digestion, as seen in previous gap repair methods.^28^ This improves on the original YTK workflow which uses PCR amplicons,^4^ as digestion is quick, requires no purification, and does not introduce mutations. The resulting linear DNA fragments can then be directly transformed into yeast, where overlapping homology is gap repaired in vivo in a defined order at the desired locus, using the transient CRISPR-Cas9 method to boost efficiencies. Successful installation of the cassettes into the genome is then screened by picking white colonies, due to the loss of the mScarlet dropout in the integration vector.

MYT provides 18 Level 1 assembly cassettes (pMYT039–056) that encompass all necessary combinations of connector sequences to achieve between 2 and 10 TUs in the Level 2 assembly (Supporting Table 4). Furthermore, 18 spacers (pMYT057–074) are included to provide flexibility in the multigene assembly by substituting for any TU (Supporting Table 5). To avoid the reuse of terminators in multigene constructs, we have also added four new terminators to supplement the six included in YTK, all of which have undergone full characterization (Supporting Figure 8). As with any other part, we strongly recommended using unique sequences at each position within a multigene construct to avoid stability issues in yeast due to recombination.

To evaluate the accuracy of the gap repair assembly method, we reused the 10 reporters described in Figure 2E to now fill all 10 TU positions in a multigene assembly. In a one-pot reaction, we combined 200 fmol of each cassette (∼500 ng), 50 fmol (∼50 ng) of the Int.1 integration vector (containing the *URA3* marker), and 50 fmol (∼200 ng) of a transient CRISPR-Cas9 plasmid targeting the Int.1 locus. We then digested the DNA with NotI and transformed the DNA directly into yeast. The reporter output of the 10 genes was then measured from 10 randomly chosen colonies from three independent transformations. Our results confirmed that all genes were present in each instance, demonstrating the high fidelity of the gap repair assembly method (Figure 4C). The successful assembly of a representative clone was also validated by colony PCR, using designed priming sites that amplify across the assembly junctions (Supporting Figure 9).

Although we did not see any mis-assemblies with the gap repair method, it is sometimes favored to assemble multigene constructs in vitro so they can be validated before transformation (e.g., by long-read sequencing), or when integrating vectors at multiple locations, when gap repair should not be used (Figure 4A,D). To accommodate the four additional TUs, we extended the original YTK Level 2 Golden Gate overhangs using the NEBridge GetSet tool to predict four further high-fidelity 4-bp sequences (Supporting Figure 10). We then confirmed the accuracy of the overhangs by assembling 10 Level 1 spacer cassettes into an integration vector and screened for the correct assembly length by colony PCR. Again, this work showed a 100% success rate from 10 random colonies from three independent assemblies (Figure 4E) and was further confirmed by direct sequencing.

### Set of Three Orthogonal Inducible Promoter Systems

**Figure 5:**
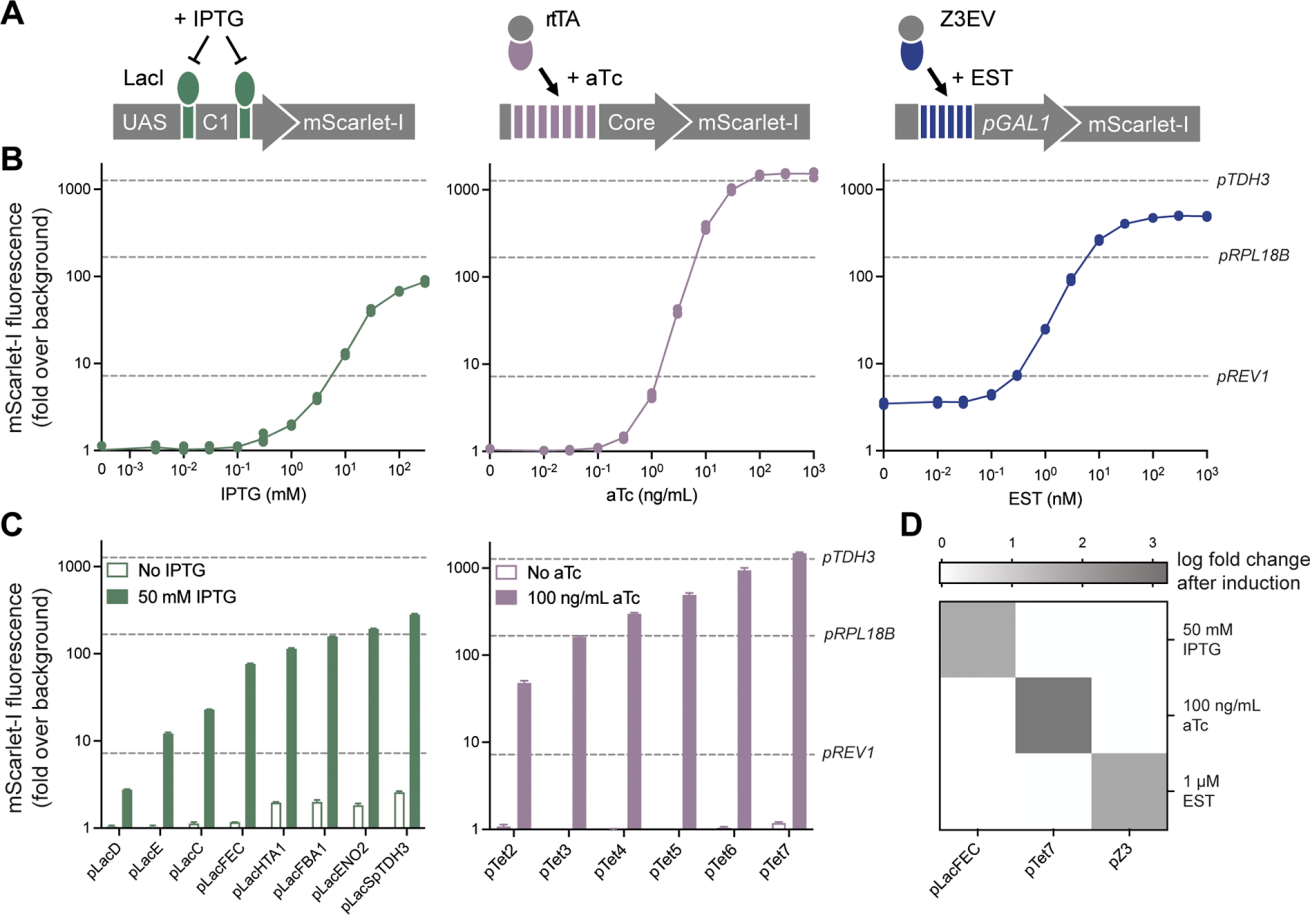
Orthogonal inducible promoters. (A) Inducible promoter designs of three inducible systems, using the IPTG repressible LacI protein (green), the aTc inducible rtTA protein (purple), and EST inducible Z3EV protein (blue), highlighting the mechanism of action and promoter architecture. (B) Dose–response curves of the three inducible systems using the pLacFEC (green), pTet7 (purple), and pZ3 (blue) promoters driving the expression of mScarlet-I, over a range of respective IPTG, aTc, and EST concentrations. (C) LacI and rtTA promoter libraries, showing the on and off response to 50 mM IPTG and 100 ng/mL aTc, respectively. Experimental measurements are mScarlet-I levels per cell as determined by flow cytometry and shown as the mean ± SD from three biological replicates and normalized to an untransformed control. mScarlet-I fluorescence from the *TDH3*, *RPL18B*, and *REV1* promoters from YTK are shown as a reference of relative expression. (D) Orthogonality between inducible systems. Expression of LacI, rtTA, and Z3EV were present in all conditions, while varying the promoter driving the expression of mScarlet-I and separately inducing with each ligand. Experimental measurements are fold change in mScarlet-I levels per cell determined by flow cytometry and shown as the mean from three biological replicates.

The original YTK plasmid collection included two inducible promoters, *pGAL1* and *pCUP1*. However, their use requires modifying the growth medium by switching to a galactose carbon source or by adding copper(II) sulfate.^4^ Therefore, to increase the versatility of conditional gene expression in yeast, MYT provides three more inducible promoter systems that are responsive to common, inexpensive, and physiologically inert ligands that can be added to any growth medium (Figure 5A).

The three inducible systems were based on the LacI,^29^ rtTA,^30^ and Z3EV^31^ transcription factors, which are responsive to isopropyl β-D-1-thiogalactopyranoside (IPTG), anhydrotetracycline (aTc), and β-estradiol (EST), respectively (Figure 5B, Supporting Figure 11). The MYT promoter systems induced by these ligands were tuned to achieve a high dynamic range, while expression of the regulating transcription factors was kept to the weakest available promoters in YTK to minimize their burden on the host cell (*pPSP2*-LacI; *pRAD27*-rtTA; *pREV1*-Z3EV). Many rounds of development were performed to reduce the basal (uninduced) level of expression while maintaining a high level of maximum signaling when fully induced.

The LacI and rtTA systems were built from the bottom up, using previously established design rules^17,25,32^ and introducing additional principles to allow for promoter diversification by changing the upstream activating sequence (UAS) or the number of transcription factor binding sites, respectively. Using this approach, we created a library of eight LacI and six rtTA promoters with different maximum expression levels to facilitate fine-tuning of the on/off response (Figure 5C). Taken together, these two inducible systems span an expression range beyond that of the weakest (*pREV1*) to strongest (*pTDH3*) promoters included in YTK. As the LacI promoters rely on repression, they can also be used as constitutive promoters by omitting LacI from the system (Supporting Figure 12). The third inducible system, controlled by Z3EV, consists of a single promoter, ported directly from McIsaac et al.,^31^ and works over a wide operational range of input concentrations (0.1–100 nM). Small changes were made to remove nonpermissible restriction enzyme sites and the additional cloning sites for use in the YTK ecosystem.

Orthogonality is an important characteristic for inducible systems, as this allows the discrete programming of multiple genes within a single cell.^33,34^ To demonstrate the orthogonality of the three inducible systems, we combined the three transcription factors and a representative promoter from each system in all configurations and stimulated the cells with saturating concentrations of each inducer, independently (Figure 5D). Our results revealed no detectable levels of crosstalk, thereby confirming that the systems are indeed orthogonal to each other. Furthermore, the sequences for all 15 promoters included here are distinct, sharing no more than 24 bp of repeat sequences. This design minimizes the risk of their recombination when used on the same genetic construct in yeast. Finally, to accommodate the simultaneous use of all three inducible systems, we have included with MYT the parts encoding three popular fluorescent proteins, mScarlet-I,^35^ mNeonGreen,^36^ and mTagBFP2,^37^ that have minimal spectral overlaps and favorable expression characteristics for yeast.^38^

## Summary

We have described a new synthetic biology toolkit for the yeast *Saccharomyces cerevisiae*, termed the Multiplex Yeast Toolkit (MYT). MYT builds upon the highly popular YTK system and introduces numerous new features to extend its capabilities for multiplexed engineering.

Specifically, we have included integration vectors that target 10 newly defined intergenic loci across the *S. cerevisiae* genome. The loci are conserved between common laboratory strains, and we include 10 yeast selectable markers to provide a high level of application flexibility and simplify the sharing of constructs between laboratories.

In addition to the integration vectors, we developed a user-friendly CRISPR-Cas9 system that allows for markerless integration of all 10 vectors with high efficiency. This system has also been adapted for transient expression to boost the integration efficiency of vectors containing a selectable marker, thus avoiding laborious genotyping during routine transformations.

To further expand the toolkit’s capabilities, we have increased the number of transcriptional units to 10 and introduced a simple gap repair method that saves time by allowing for the installation of a multigene construct from Level 1 cassettes directly into the yeast genome. We also developed three highly tuned and orthogonal promoter systems that enable the conditional expression of multiple genes within the same system, free from crosstalk.

Finally, to help scientists fully realize the capabilities of MYT, we have included detailed documentation that provides step-by-step guidance on how to use the toolkit. In summary, the Multiplex Yeast Toolkit represents a significant advancement in the yeast synthetic biologist’s toolbox, all while being backward compatible to the widely used YTK system, enabling new and exciting possibilities for yeast engineering.

## Methods

### Bacterial Strains and Growth Media

NEB Turbo *E. coli* was used for all cloning experiments. Selection and growth of *E. coli* were performed in Lysogeny Broth (LB) medium at 37 °C with aeration. Except for generating competent cells, the LB medium was supplemented with the appropriate antibiotics for plasmid maintenance (chloramphenicol 34 μg/mL, carbenicillin 100 μg/mL, kanamycin 50 μg/mL, or spectinomycin 100 μg/mL). Note: as NEB Turbo cells are RecA^+^, plasmid multimers are often seen during cloning using long-read sequencing. However, as DNA is digested during the Golden Gate assembly workflow and prior to transformation, these issues are mitigated and the benefits of the fast growth can be exploited.

### Bacterial Transformations

Chemically competent *E. coli* cells were created following the TSS protocol for KCM transformations.^39^ A colony of *E. coli* was grown to saturation overnight in 10 mL of LB and then split into two 2 L baffled flasks with 500 mL of LB. The culture was grown for 2–3 h to OD_600_ ∼ 1.0, chilled on ice to stop growth, and centrifuged at 4000*g* at 4 °C for 10 min. The supernatant was then discarded, and the cell pellets were resuspended by aspiration in 100 mL of ice-cold TSS (85 mL of LB, 10 g of PEG-3350, 5 mL of DMSO, and 2 mL of 1 M MgCl_2_). 200 μL of the cell suspension was then aliquoted into 0.2 mL PCR strip tubes, flash frozen in liquid nitrogen (or dry ice), and put into a −80 °C freezer for long-term storage. To transform the DNA, 50 μL of 5× KCM (500 mM KCl, 150 mM CaCl_2_, 250 mM MgCl_2_) was added to 200 μL of the competent cells after 10 min of thawing on ice. 50 μL of the competent cell-KCM cocktail was then added to 1–10 μL of DNA and kept on ice for 5 min. The cells were then heat-shocked in a water bath at 42 °C for 1 min, transferred back to ice for 1 min, and then recovered at 37 °C for 1 h (shaking not necessary). Cells were then plated on solid LB medium supplemented with the appropriate antibiotics.

### Yeast Strains and Growth Media

*Saccharomyces cerevisiae* strain BY4741 (*MAT*a *his3*Δ*1 leu2*Δ*0 met17*Δ*0 ura3*Δ*0*) was used for all experiments. Yeast extract peptone dextrose (YPD) was used for culturing cells in preparation for transformation and growth curve characterization: 1% (w/v) Bacto Yeast Extract (Merck), 2% (w/v) Bacto Peptone (Merck), 2% (w/v) glucose (VWR). All flow cytometry experiments were performed in Synthetic Complete (SC) medium with 2% (w/v) glucose (VWR), 0.67% (w/v) Yeast Nitrogen Base without amino acids (Sunrise Science Products), and 0.8 g/L complete supplement mix (CSM; Sunrise Science Products). For auxotrophic selection, SC medium minus the appropriate supplement was used instead of CSM (SC-Ura, SC-Leu, SC-His, and SC-Met; Sunrise Science Products). For antibiotic selection, the appropriate antibiotic was added to the SC medium (200 μg/mL G418, 100 μg/mL nourseothricin, 400 μg/mL hygromycin, or 200 μg/mL zeocin). Solid medium was prepared with 2% (w/v) agar (VWR).

### Yeast Transformations

Yeast cells were transformed using the lithium acetate protocol, adapted from Gietz and Woods.^40^ A yeast colony was picked from a plate and grown to saturation overnight in 2 mL of YPD medium. The following morning the cells were diluted 1:100 in YPD (OD_600_ ∼ 0.175) and grown for 4–6 h to OD_600_ 0.6–0.8 (1 mL of culture corresponds to a single transformation). Cells were pelleted at 3000*g* for 3 min in a large benchtop centrifuge at room temperature and washed once with half the starting volume of 0.1 M lithium acetate (LiOAc) (Sigma) and repelleted. Cells were then resuspended in 0.1 M LiOAc to a total volume of 100 μL/transformation. 100 μL of cell suspension was then distributed into 1.5 mL reaction tubes and pelleted at 8000 rpm for 3 min on a small benchtop centrifuge at room temperature. Cells were resuspended in 64 μL of DNA-salmon sperm DNA mixture (10 μL of boiled salmon sperm DNA (Invitrogen) + DNA + ddH_2_O) and left to incubate at room temperature for 30 min. 294 μL of PEG-LiOAc mixture (260 μL 50% (w/v) PEG-3350 (Sigma) + 36 μL 1 M LiOAc) was then added, gently vortexed for five seconds, and incubated at room temperature for 30 min. The yeast transformation mixture was then transferred to a water bath at 42 °C for 15 min. Cells were pelleted at 8000 rpm in a small benchtop centrifuge for 20 s at room temperature and resuspended in 0.1–1 mL of sterile water (for auxothrophic selection) or YPD (for antibiotic selection), and plated onto the appropriate synthetic dropout medium (after 10 min at room temperature) or antibiotic medium (after 3 h recovery at 30 °C). See Supporting Table 6 for guidelines on the resuspension volume and the volume of cells to the plate for each type of experiment.

DNA was digested with NotI prior to transformation, as follows: plasmid DNA (various amounts), 1 μL of CutSmart buffer (NEB), 0.2 μL of NotI (NotI-HF, NEB), and up to 10 μL of sterile H_2_O. We recommend overnight digestions (16 h at 37 °C) followed by a heat kill (20 min at 65 °C). However, if shorter times are required, more NotI can be added. Additionally, the 10 μL reaction volume can be increased if the combined DNA volumes exceed 10 μL, but we recommend keeping volume as low as possible to maintain high transformation efficiencies. The entire reaction was then combined with water and boiled salmon sperm DNA to 64 μL, as described above, and directly transformed into yeast without a cleanup step. For multiplexed markerless CRISPR-Cas9 experiments using more than four integration vectors, integration vectors should be individually digested and column purified before transformation to achieve high efficiency. Column purification for experiments using four or fewer integration vectors will improve efficiencies but may be unnecessary. See Supporting Table 6 for guidelines on the amount of DNA to use for each type of experiment.

### Design of Integration Loci

After establishing the design principles, described in Supporting Table 1, we identified potential integration loci by manually searching through the *S. cerevisiae* reference genome (S288C - PRJNA43747) using the Genome Browser (JBrowse 1.16.9) on the Saccharomyces Genome Database (yeastgenome.org). Candidate sites were first identified by searching for multi-kilobase gaps between genes. These sites were then imported and analyzed in Benchling (Benchling.com) to determine their suitability according to the remaining design criteria. BLAST (blast.ncbi.nlm.nih.gov) was used to search for sequence similarity between common laboratory strains of yeast and identify repeated sequences within the BY4741 genome. Predicted high on- and off-target CRISPR-Cas9 gRNAs targets were then identified using the CRISPR tool in Benchling, searching in a window that would position the integration site >1 and 0.5 kb from the start and stop codon of neighboring genes, respectively. For a list of CRISPR-Cas9 gRNA spacers, see Supporting Table 7. The homology arms for the integration vectors were then designed around the CRISPR-Cas9 target site, extending ∼500 bp on either side. See Supporting Figure 2 for a full description of the homology arm design.

### Level 1 and Level 2 Golden Gate Assembly Protocol

All DNA insert and plasmid concentrations were set to 50 fmol/μL and plasmid backbone concentrations were set to 25 fmol/μL prior to assembly. Golden Gate reaction mixtures were prepared as follows: 0.5 μL of each DNA insert or plasmid, 1 μL of T4 DNA Ligase buffer (NEB), 0.5 μL of T4 DNA Ligase (400 U/μL, NEB), 0.5 μL of restriction enzyme, and water to bring the final volume to 10 μL. The restriction enzymes used were either BsaI (20 U/μL BsaI-HF v2, NEB) or BsmBI (10 U/μL BsmBI v2, NEB). Reaction mixtures were incubated in a thermocycler according to the following program: 25 cycles of digestion (BsaI, 37 °C for 2 min or BsmBI, 42 °C for 2 min) and ligation (16 °C for 5 min), followed by a final digestion step (55 °C for 10 min) and a heat inactivation step (80 °C for 10 min). For reactions with >6 inserts, we increased the digestion step to 5 min. For a list of plasmids included in the MYT Addgene plate, see Supporting Table 8. The entire reaction was then transformed into *E. coli* and plated on LB medium plus the appropriate antibiotic. Bacterial colonies were screened for the loss of sfGFP or mScarlet using a blue light transilluminator and the plasmid DNA was prepped from nonfluorescent clones and validated using restriction digestion (NotI-HF, NEB).

### Marker Assembly

DNA concentrations were as described above. For the assembly of a marker (pMYT029–038) into an integration vector/spacer (pMYT075–094) or the CRISPR-Cas9 plasmid (pMYT095), the Golden Gate reaction was prepared as follows: 1 μL of marker plasmid, 0.5 μL of plasmid backbone, 1 μL of T4 DNA Ligase buffer (NEB), 0.5 μL of T4 DNA Ligase (400 U/μL, NEB), 0.5 μL of BbsI (10 U/μL BpiI, Thermo Scientific), and 6.5 μL H_2_O. Reaction mixtures were incubated in a thermocycler according to the following program: 10 cycles of digestion and ligation (37 °C for 2 min, 16 °C for 5 min) followed by a final digestion step (55 °C for 10 min), and a heat inactivation step (80 °C for 10 min). The entire reaction was then transformed into *E. coli* and plated on LB medium plus the appropriate antibiotic. As there is no change to fluorescent marker expression, plasmid DNA from a random colony was prepped and validated using restriction digestion (NotI-HF, NEB).

### gRNA-tRNA Array Assembly

Individual gRNA-tRNA fragment PCRs were set up in 20 μL volume reactions as follows: 1 μL of diluted pMYT095/96 plasmid (∼2 ng/μL), 1 μL of each primer (10 μM), 7 μL of H_2_O, and 10 μL of Q5 High-Fidelity 2× Master Mix (NEB). Reactions were then transferred to a thermocycler under the following conditions: 30 s at 98 °C, (10 s at 98 °C, 20 s at 57 °C, 30 s at 72 °C) × 30 cycles, 30 s at 72 °C. To purify gRNA fragments after PCR, 4 μL of 6× loading dye (NEB) was added to the completed reaction and run on a 1% agarose gel until total separation of DNA bands. After gel electrophoresis, gel bands were excised and DNA was extracted using Zymoclean Gel DNA Recovery kit (Zymo Research), following manufacturer instruction, and the DNA concentration was measured (NanoDrop One). Golden Gate reaction mixtures were prepared as follows: 10 ng of each gRNA-tRNA fragment, 100 ng of pMYT095/96, 1 μL T4 DNA Ligase buffer (NEB), 0.5 μL T4 DNA Ligase (400 U/μL, NEB), 0.5 μL BsaI (20 U/μL BsaI-HF v2, NEB), and water to bring the final volume to 10 μL. Reaction mixtures were incubated in a thermocycler according to the following program: 25 cycles of digestion and ligation (37 °C for 2 min, 16 °C for 5 min) followed by a final digestion step (55 °C for 10 min), and a heat inactivation step (80 °C for 10 min). For reactions with >6 gRNA-tRNA fragments, we increased the digestion step to 5 min. The entire reaction was then transformed into *E. coli*, and plasmid DNA from nonfluorescent colonies was prepped and sent for Sanger sequencing. For a list of primers used for sequencing and to create the gRNA-tRNA fragments used in this study, see Supporting Table 9.

### Flow Cytometry

On day 1, strains were picked into 500 μL of synthetic complete (SC) medium and grown in a 2.2 mL 96 deep-well plate at 30 °C in an Infors HT Multitron, shaking at 900 rpm overnight. On day 2, saturated strains were then diluted 1:100 into 500 μL of fresh SC media and incubated for a further 6 h before measurement. Cell fluorescence was measured using an Attune NxT Flow Cytometer (Thermo Scientific) using the following settings: FSC 300 V, SSC 350 V, BL1 500 V. Fluorescence data was collected from 10,000 cells for each experiment and analyzed using FlowJo software, gating for singlets using FSC-A vs FSC-H. No further gating was performed on yeast populations. For the inducible promoter experiments, strains were back diluted 1:100 into fresh medium containing the inducer on day 2 and grown for 16 h overnight. On day 3, saturated strains were then diluted 1:100 into 500 μL of fresh SC medium containing the inducer and incubated for a further 6 h before measurement.

### Optical Density Measurements in Reporter Assays

Single colonies of each strain were grown to saturation overnight in 500 μL of YPD medium in a 2.2 mL 96 deep-well plate at 30 °C in an Infors HT Multitron, shaking at 900 rpm. The next day, the yeast cultures were back diluted 1:100 in 500 μL of selective medium and incubated in the same conditions. After 16 h, 200 μL was transferred to a 96-well clear bottom plate and the OD_700_ was measured using a SpectraMax plate reader (Molecular Devices) using SoftMax Pro (v7) software.

### Growth Curves

Single colonies of each strain were grown to saturation overnight in 500 μL of YPD medium in a 2.2 mL 96 deep-well plate at 30 °C in an Infors HT Multitron, shaking at 900 rpm. The next day, the yeast cultures were back diluted 1:100 to an OD_700_ of ∼0.175 in 100 μL of fresh YPD medium in a 96-well clear, flat-bottom microplate (Corning). OD_700_ was then measured over 24 h using a SpectraMax plate reader (Molecular Devices) using SoftMax Pro (v7) software, taking measurements every 15 min with shaking at 30 °C in between readings. Maximum growth rate was then calculated in Microsoft Excel according to the equation (ln(OD_600_(*t* + 3h)/OD_600_(*t*))/3 over the linear range, where *t* is the time in hours.

### Colony PCR

Genotyping of yeast was performed directly on yeast suspensions using Phire Plant Direct PCR Master Mix (Thermo Scientific). For analysis of yeast on solid medium, colonies were picked and resuspended in 50 μL of sterile water and 1 μL of the suspension was used in the PCR. For analysis of yeast in liquid medium, 1 μL of saturated culture was used in the PCR. Reactions were set up as follows: 1 μL yeast suspension, 0.5 μL of each primer (10 μM), 3 μL of H_2_O, and 5 μL of 2× Phire Plant Direct PCR Master Mix (Thermo Scientific). Reactions were then transferred to a thermocycler under the following conditions: 5 min at 98 °C, (5 s at 98 °C, 5 s at 60 °C, 30 s at 72 °C) × 30 cycles, 30 s at 72 °C. All colony PCR primers were designed for annealing at 60 °C. For a list of colony PCR primers used in this study see Supporting Tables 10 and 11.

### Colony Counts

Colony counting was performed by hand. For individual markerless CRISPR-Cas9 efficiency calculations, red-fluorescent colonies were counted by using a blue light transilluminator.

### Statistics and Reproducibility

All data were analyzed in Excel (Microsoft) and Prism 9 (GraphPad). Error bars represent the standard deviation as noted in the figure legend, and ANOVA was used for statistical analyses with Prism 9 (GraphPad). The respective numbers of replicates are given in the figure legend, and all replicates are included in the manuscript.

## Data Availability

All plasmids described in this toolkit are available from Addgene (http://www.addgene.org), Kit 1000000229: Multiplex Yeast Toolkit (MYT). A project folder containing annotated MYT plasmid files, genomic integration loci, oligonucleotides, example CRISPR-Cas9 gRNA assemblies, and relevant protocols can be found at https://benchling.com/will_shaw/f_/gxQ8Vznl-multiplex-yeast-toolkit-shaw-2023/.

## Supporting Information Available

Supporting figures that include additional data from experiments described in the text, (Supporting Figure 1) Position of the MYT integration loci on the *Saccharomyces cerevisiae* S288C genome; (Supporting Figure 2) MYT integration loci homology arm design; (Supporting Figure 3) Individual reporter responses from multiplexed vector integration using markerless CRISPR-Cas9; (Supporting Figure 4) Colony PCR validation of a representative 10x markerless CRISPR-Cas9 strain; (Supporting Figure 5) Individual reporter responses for select 8x vector integration using markerless CRISPR-Cas9; (Supporting Figure 6) Colony PCR screening of select 8x spacer integration using markerless CRISPR-Cas9; (Supporting Figure 7) Fidelity of multiplexed vector integration using transient CRISPR-Cas9; (Supporting Figure 8) Extended terminator library characterization; (Supporting Figure 9) Colony PCR validation of a representative 10x gap repair assembly strain; (Supporting Figure 10) Estimated ligation fidelity of MYT Level 2 assembly overhangs; (Supporting Figure 11) Flow cytometry histograms of the inducible promoter systems; (Supporting Figure 12) LacI promoter expression without LacI. Supporting tables that list additional information described in the text; (Supporting Table 1) MYT integration loci design criteria; (Supporting Table 2) Integration vector yeast strain compatibility; (Supporting Table 3) gRNA-tRNA array primer design; (Supporting Table 4) Organization of Level 1 assembly cassettes in a Level 2 multigene assembly; (Supporting Table 5) Organization of Level 1 assembly spacers in a Level 2 multigene assembly; (Supporting Table 6) Yeast transformation and plating guidelines; (Supporting Table 7) CRISPR-Cas9 spacers; (Supporting Table 8) Multiplex Yeast Toolkit plasmids; (Supporting Table 9) CRISPR-Cas9 primers used in this study; (Supporting Table 10) Colony PCR primers for verification of vector integration; (Supporting Table 11) Colony PCR primers for verification of gap repair assembly; Supporting Text that describe and illustrate the architecture of the MYT plasmids, how they are assembled, and various genome integration methods (PDF)
MYT plasmid files (ZIP)
Characterization data for expression at the MYT integration loci, the extended terminator library, and the inducible promoters (XLSX)

## References

[ref1] Khalil A. S.; Collins J. J. Synthetic biology: applications come of age. Nat. Rev. Genet. 2010, 11, 367–379. 10.1038/nrg2775. 20395970 PMC2896386

[ref2] Chen B.; Lee H. L.; Heng Y. C.; Chua N.; Teo W. S.; Choi W. J.; Leong S. S. J.; Foo J. L.; Chang M. W. Synthetic biology toolkits and applications in Saccharomyces cerevisiae. Biotechnol. Adv. 2018, 36, 1870–1881. 10.1016/j.biotechadv.2018.07.005. 30031049

[ref3] Malcı K.; Watts E.; Roberts T. M.; Auxillos J. Y.; Nowrouzi B.; Boll H. O.; Nascimento C. Z. S.; Do Andreou A.; Vegh P.; Donovan S.; Fragkoudis R.; Panke S.; Wallace E.; Elfick A.; Rios-Solis L. Standardization of Synthetic Biology Tools and Assembly Methods for Saccharomyces cerevisiae and Emerging Yeast Species. ACS Synth. Biol. 2022, 11, 2527–2547. 10.1021/acssynbio.1c00442. 35939789 PMC9396660

[ref4] Lee M. E.; DeLoache W. C.; Cervantes B.; Dueber J. E. A Highly Characterized Yeast Toolkit for Modular Multipart Assembly. ACS Synth. Biol. 2015, 4, 975–986. 10.1021/sb500366v. 25871405

[ref5] Guo Y.; Dong J.; Zhou T.; Auxillos J.; Li T.; Zhang W.; Wang L.; Shen Y.; Luo Y.; Zheng Y.; Lin J.; Chen G.-Q.; Wu Q.; Cai Y.; Dai J. YeastFab: the design and construction of standard biological parts for metabolic engineering in Saccharomyces cerevisiae. Nucleic Acids Res. 2015, 43, e88–e88. 10.1093/nar/gkv464. 25956650 PMC4513847

[ref6] Agmon N.; Mitchell L. A.; Cai Y.; Ikushima S.; Chuang J.; Zheng A.; Choi W.-J.; Martin J. A.; Caravelli K.; Stracquadanio G.; Boeke J. D. Yeast Golden Gate (yGG) for the Efficient Assembly of S. cerevisiae Transcription Units. ACS Synth. Biol. 2015, 4, 853–859. 10.1021/sb500372z. 25756291

[ref7] Jessop-Fabre M. M.; Jakočiu̅nas T.; Stovicek V.; Dai Z.; Jensen M. K.; Keasling J. D.; Borodina I. EasyClone-MarkerFree: A vector toolkit for marker-less integration of genes into Saccharomyces cerevisiae via CRISPR-Cas9. Biotechnol. J. 2016, 11, 1110–1117. 10.1002/biot.201600147. 27166612 PMC5094547

[ref8] Otto M.; Skrekas C.; Gossing M.; Gustafsson J.; Siewers V.; David F. Expansion of the Yeast Modular Cloning Toolkit for CRISPR-Based Applications, Genomic Integrations and Combinatorial Libraries. ACS Synth. Biol. 2021, 10, 3461–3474. 10.1021/acssynbio.1c00408. 34860007 PMC8689691

[ref9] Weber E.; Engler C.; Gruetzner R.; Werner S.; Marillonnet S. A Modular Cloning System for Standardized Assembly of MultigeneConstructs PLoS One 2011, 6, e16765, 10.1371/journal.pone.0016765 21364738 PMC3041749

[ref10] Engler C., Kandzia R., Marillonnet S. A One Pot, One Step, Precision Cloning Method with High ThroughputCapability PLoS One 2008, 3, e3647, 10.1001/archinternmed.2008.513. 18985154 PMC2574415

[ref11] Bird J. E.; Marles-Wright J.; Giachino A. A User’s Guide to Golden Gate Cloning Methods and Standards. ACS Synth. Biol. 2022, 11, 3551 10.1021/acssynbio.2c00355. 36322003 PMC9680027

[ref12] Bashor C. J.; Patel N.; Choubey S.; Beyzavi A.; Kondev J.; Collins J. J.; Khalil A. S. Complex signal processing in synthetic gene circuits using cooperative regulatory assemblies. Science 2019, 364, 593–597. 10.1126/science.aau8287. 31000590 PMC6650298

[ref13] Bragdon M. D. J.; Patel N.; Chuang J.; Levien E.; Bashor C. J.; Khalil A. S. Cooperative assembly confers regulatory specificity and long-term genetic circuit stability. Cell 2023, 186, 3810–3825.e18. 10.1016/j.cell.2023.07.012. 37552983 PMC10528910

[ref14] An-adirekkun J. My; Stewart C. J.; Geller S. H.; Patel M. T.; Melendez J.; Oakes B. L.; Noyes M. B.; McClean M. N. A yeast optogenetic toolkit (yOTK) for gene expression control in Saccharomyces cerevisiae. Biotechnol. Bioeng. 2020, 117, 886–893. 10.1002/bit.27234. 31788779 PMC7015765

[ref15] Mukherjee M.; Wang Z. Q. A well-characterized polycistronic-like gene expression system in yeast. Biotechnol. Bioeng. 2023, 120, 260–271. 10.1002/bit.28247. 36168285

[ref16] Shaw W. M.; Yamauchi H.; Mead J.; Gowers G.-O. F.; Bell D. J.; Öling D.; Larsson N.; Wigglesworth M.; Ladds G.; Ellis T. Engineering a Model Cell for Rational Tuning of GPCR Signaling. Cell 2019, 177, 782–796.e27. 10.1016/j.cell.2019.02.023. 30955892 PMC6476273

[ref17] Shaw W. M.; Studená L.; Roy K.; Hapeta P.; McCarty N. S.; Graham A. E.; Ellis T.; Ledesma-Amaro R. Inducible expression of large gRNA arrays for multiplexed CRISPRai applications. Nat. Commun. 2022, 13, 4984 10.1038/s41467-022-32603-7. 36008396 PMC9411621

[ref18] Ortiz L.; Pavan M.; McCarthy L.; Timmons J.; Densmore D. M. Automated Robotic Liquid Handling Assembly of Modular DNA Devices. J. Vis. Exp. 2017, 2017, 54703 10.3791/54703. PMC5755516 29286379

[ref19] Chao R.; Mishra S.; Si T.; Zhao H. Engineering biological systems using automated biofoundries. Metab. Eng. 2017, 42, 98–108. 10.1016/j.ymben.2017.06.003. 28602523 PMC5544601

[ref20] Baek S.; Utomo J. C.; Lee J. Y.; Dalal K.; Yoon Y. J.; Ro D.-K. The yeast platform engineered for synthetic gRNA-landing pads enables multiple gene integrations by a single gRNA/Cas9 system. Metab. Eng. 2021, 64, 111–121. 10.1016/j.ymben.2021.01.011. 33549837

[ref21] Bourgeois L.; Pyne M. E.; Martin V. J. J. A Highly Characterized Synthetic Landing Pad System for Precise Multicopy Gene Integration in Yeast. ACS Synth. Biol. 2018, 7, 2675–2685. 10.1021/acssynbio.8b00339. 30372609

[ref22] Babaei M.; Sartori L.; Karpukhin A.; Abashkin D.; Matrosova E.; Borodina I. Expansion of EasyClone-MarkerFree toolkit for Saccharomyces cerevisiae genome with new integration sites. FEMS Yeast Res. 2021, 21, foab027 10.1093/femsyr/foab027. 33893795 PMC8112480

[ref23] Apel A. R.; D’Espaux L.; Wehrs M.; Sachs D.; Li R. A.; Tong G. J.; Garber M.; Nnadi O.; Zhuang W.; Hillson N. J.; Keasling J. D.; Mukhopadhyay A. A Cas9-based toolkit to program gene expression in Saccharomyces cerevisiae. Nucleic Acids Res. 2017, 45, 496–508. 10.1093/nar/gkw1023. 27899650 PMC5224472

[ref24] Ronda C.; Maury J.; Jakočiu̅nas T.; Baallal Jacobsen S. A.; Germann S. M.; Harrison S. J.; Borodina I.; Keasling J. D.; Jensen M. K.; Nielsen A. T. CrEdit: CRISPR mediated multi-loci gene integration in Saccharomyces cerevisiae. Microb. Cell Fact. 2015, 14, 97 10.1186/s12934-015-0288-3. 26148499 PMC4492099

[ref25] Chen Y.; Zhang S.; Young E. M.; Jones T. S.; Densmore D.; Voigt C. A. Genetic circuit design automation for yeast. Nat. Microbiol. 2020, 5, 1349–1360. 10.1038/s41564-020-0757-2. 32747797

[ref26] Zhang Y.; Wang J.; Wang Z.; Zhang Y.; Shi S.; Nielsen J.; Liu Z. A gRNA-tRNA array for CRISPR-Cas9 based rapid multiplexed genome editing in Saccharomyces cerevisiae. Nat. Commun. 2019, 10, 1053 10.1038/s41467-019-09005-3. 30837474 PMC6400946

[ref27] Mülleder M.; Campbell K.; Matsarskaia O.; Eckerstorfer F.; Ralser M. Saccharomyces cerevisiae single-copy plasmids for auxotrophy compensation, multiple marker selection, and for designing metabolically cooperating communities. F1000Res. 2016, 5, 2351 10.12688/f1000research.9606.1. 27830062 PMC5081161

[ref28] Mitchell L. A.; Chuang J.; Agmon N.; Khunsriraksakul C.; Phillips N. A.; Cai Y.; Truong D. M.; Veerakumar A.; Wang Y.; Mayorga M.; Blomquist P.; Sadda P.; Trueheart J.; Boeke J. D. Versatile genetic assembly system (VEGAS) to assemble pathways for expression in S. cerevisiae. Nucleic Acids Res. 2015, 43, 6620–6630. 10.1093/nar/gkv466. 25956652 PMC4513848

[ref29] Ellis T.; Wang X.; Collins J. J. Diversity-based, model-guided construction of synthetic gene networks with predicted functions. Nat. Biotechnol. 2009, 27, 465–471. 10.1038/nbt.1536. 19377462 PMC2680460

[ref30] Gossen M.; Freundlieb S.; Bender G.; Müller G.; Hillen W.; Bujard H. Transcriptional activation by tetracyclines in mammalian cells. Science 1995, 268, 1766–9. 10.1126/science.7792603. 7792603

[ref31] McIsaac R. S.; Gibney P. A.; Chandran S. S.; Benjamin K. R.; Botstein D. Synthetic biology tools for programming gene expression without nutritional perturbations in Saccharomyces cerevisiae. Nucleic Acids Res. 2014, 42, e48 10.1093/nar/gkt1402. 24445804 PMC3973312

[ref32] Redden H.; Alper H. S. The development and characterization of synthetic minimal yeast promoters. Nat. Commun. 2015, 6, 7810 10.1038/ncomms8810. 26183606 PMC4518256

[ref33] Meyer A. J.; Segall-Shapiro T. H.; Glassey E.; Zhang J.; Voigt C. A. Escherichia coli “Marionette” strains with 12 highly optimized small-molecule sensors. Nat. Chem. Biol. 2019, 15, 196–204. 10.1038/s41589-018-0168-3. 30478458

[ref34] Sanford A.; Kiriakov S.; Khalil A. S. A Toolkit for Precise, Multigene Control in Saccharomyces cerevisiae. ACS Synth. Biol. 2022, 11, 3912–3920. 10.1021/acssynbio.2c00423. 36367334 PMC9764411

[ref35] Bindels D. S.; Haarbosch L.; van Weeren L.; Postma M.; Wiese K. E.; Mastop M.; Aumonier S.; Gotthard G.; Royant A.; Hink M. A.; Gadella T. W. J. Jr mScarlet: a bright monomeric red fluorescent protein for cellular imaging. Nat. Methods 2017, 14, 53–56. 10.1038/nmeth.4074. 27869816

[ref36] Shaner N. C.; Lambert G. G.; Chammas A.; Ni Y.; Cranfill P. J.; Baird M. A.; Sell B. R.; Allen J. R.; Day R. N.; Israelsson M.; Davidson M. W.; Wang J. A bright monomeric green fluorescent protein derived from Branchiostoma lanceolatum. Nat. Methods 2013, 10, 407–409. 10.1038/nmeth.2413. 23524392 PMC3811051

[ref37] Subach O. M., Cranfill P. J., Davidson M. W., Verkhusha V. V. An Enhanced Monomeric Blue Fluorescent Protein with the High Chemical Stability of the Chromophore PLoS One 2011 6 e28674, 10.1371/journal.pone.0028674. 22174863 PMC3234270

[ref38] Botman D.; de Groot D. H.; Schmidt P.; Goedhart J.; Teusink B. In vivo characterisation of fluorescent proteins in budding yeast. Sci. Rep. 2019, 9, 2234 10.1038/s41598-019-38913-z. 30783202 PMC6381139

[ref39] Chung C. T.; Niemela S. L.; Miller R. H. One-step preparation of competent Escherichia coli: transformation and storage of bacterial cells in the same solution. Proc. Natl. Acad. Sci. U. S. A. 1989, 86, 2172–2175. 10.1073/pnas.86.7.2172. 2648393 PMC286873

[ref40] Daniel Gietz R.; Woods R. A. Transformation of yeast by lithium acetate/single-stranded carrier DNA/polyethylene glycol method. Methods Enzymol. 2002, 350, 87–96. 10.1016/S0076-6879(02)50957-5. 12073338

