# Supplementary material for: A Multiplex MoClo Toolkit for Extensive and Flexible Engineering of Saccharomyces cerevisiae
Source: ACS Synth Biol. 2023 Nov 6;12(11):3393–405. doi: 10.1021/acssynbio.3c00423 (PMC10661031; doi:10.1021/acssynbio.3c00423)
Supplement: Supplementary file 1 — sb3c00423_si_001.pdf [file sb3c00423_si_001.pdf]

Supporting Information for

A Multiplex MoClo Toolkit for Extensive and Flexible Engineering of *Saccharomyces cerevisiae*

William M. Shaw[1,2,3,4*], Ahmad S. Khalil[1,2,5], and Tom Ellis[3,4]

[1]Biological Design Center, Boston University, Boston, MA 02215, USA
[2]Department of Biomedical Engineering, Boston University, Boston, MA 02215, USA
[3]Department of Bioengineering Imperial College London, London, SW7 2AZ, UK
[4]Imperial College Centre for Synthetic Biology, Imperial College London, London, SW7 2AZ, UK
[5]Wyss Institute for Biologically Inspired Engineering, Harvard University, Boston, MA 02115, USA
*Corresponding author willshaw@bu.edu

Supporting Figures

Supporting Figure 1. Position of the MYT integration loci on the *Saccharomyces cerevisiae* S288C genome.
Supporting Figure 2. MYT integration loci homology arm design.
Supporting Figure 3. Individual reporter responses from multiplexed vector integration using markerless CRISPR-Cas9.
Supporting Figure 4. Colony PCR validation of a representative 10x markerless CRISPR-Cas9 strain.
Supporting Figure 5. Individual reporter responses for select 8x vector integration using markerless CRISPR-Cas9.
Supporting Figure 6. Colony PCR screening of select 8x spacer integration using markerless CRISPR-Cas9.
Supporting Figure 7. Fidelity of multiplexed vector integration using transient CRISPR-Cas9.
Supporting Figure 8. Extended terminator library characterization.
Supporting Figure 9. Colony PCR validation of a representative 10x gap repair assembly strain.
Supporting Figure 10. Estimated ligation fidelity of MYT Level 2 assembly overhangs.
Supporting Figure 11. Flow cytometry histograms of the inducible promoter systems.
Supporting Figure 12. LacI promoter expression without LacI.

Supporting Tables

Supporting Table 1. MYT integration loci design criteria.
Supporting Table 2. Integration vector yeast strain compatibility.
Supporting Table 3. gRNA-tRNA array primer design.
Supporting Table 4. Organization of Level 1 assembly cassettes in a Level 2 multigene assembly.
Supporting Table 5. Organization of Level 1 assembly spacers in a Level 2 multigene assembly.
Supporting Table 6. Yeast transformation and plating guidelines.
Supporting Table 7. CRISPR-Cas9 spacers.
Supporting Table 8. Multiplex Yeast Toolkit plasmids.
Supporting Table 9. CRISPR-Cas9 primers used in this study.
Supporting Table 10. Colony PCR primers for verification of vector integration.
Supporting Table 11. Colony PCR primers for verification of gap repair assembly.

Supporting Text

Multiplex Yeast Toolkit plasmid architecture.
Multiplex Yeast Toolkit plasmid assembly methods.
Multiplex Yeast Toolkit plasmid genome integration methods.

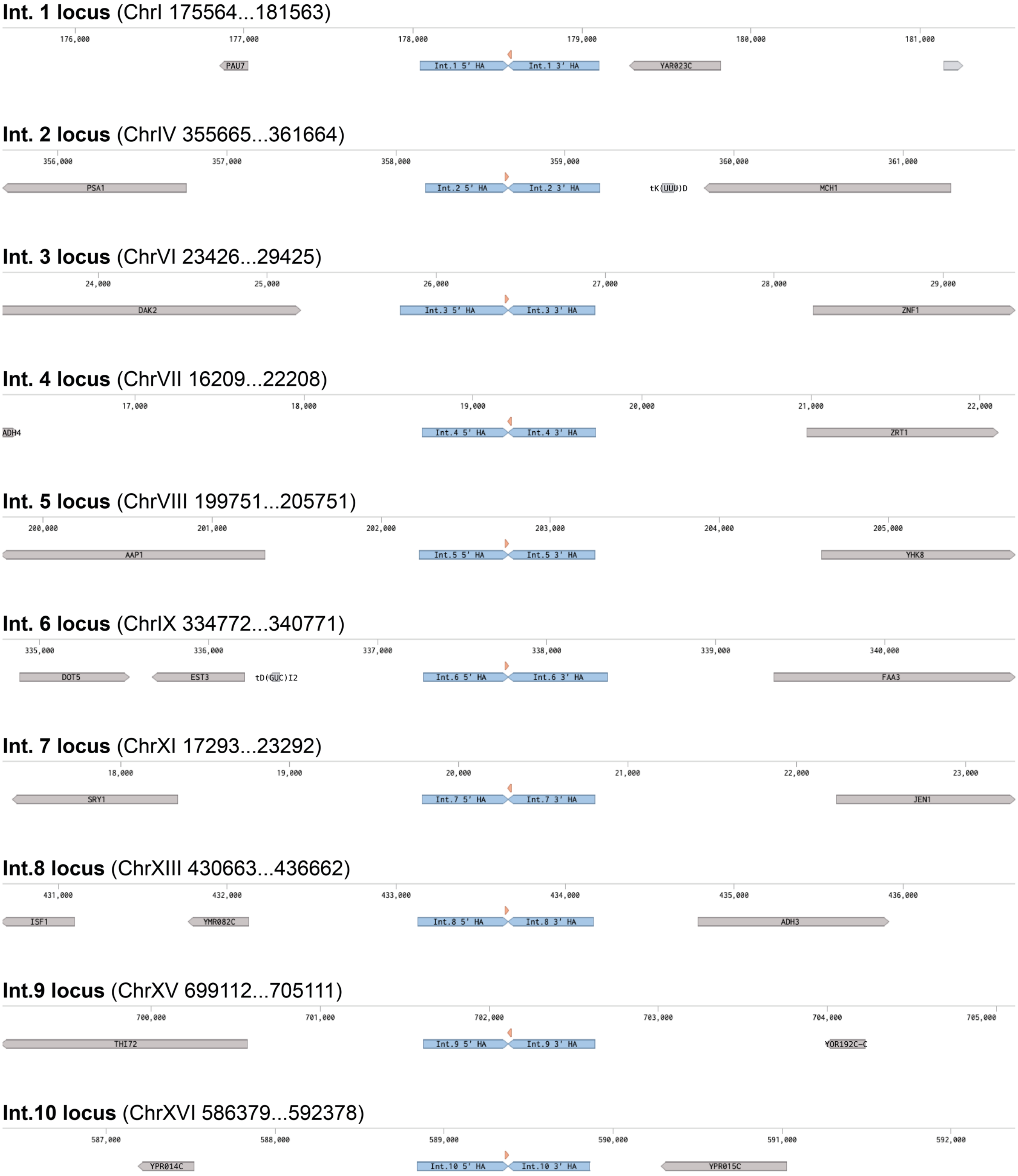

Supporting Figure 1. Position of the MYT integration loci on the *Saccharomyces cerevisiae* S288C genome. Homology arms of the integration vectors are in blue and the CRISPR-Cas9 target is in red. Native genes are in grey. Positional indexes are in 1000 bp increments. Genomic regions are the S288C reference genome, downloaded directly to Benchling using the "Select Chromosomal Region", selecting the "R64-1-1" genome, and inputting the coordinates shown above. Homology arms and gRNA targets were added as annotations and screenshots were adapted for presentation in Illustrator (Adobe).

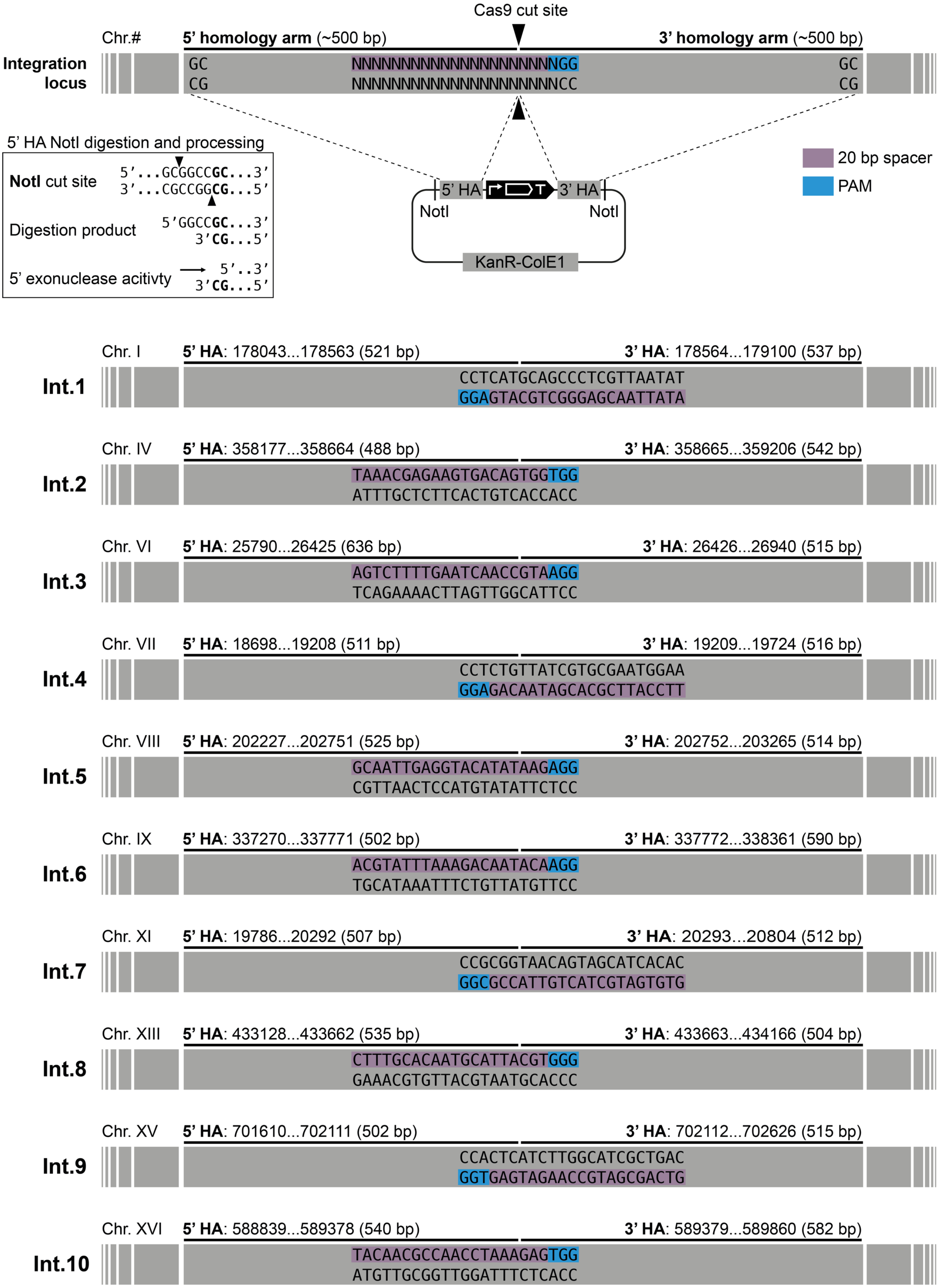
Cas9 cut site
Chr.#
5’ homology arm (~500 bp)
3’ homology arm (~500 bp)
Integration locus
GC
CG
NNNNNNNNNNNNNNNNNNNNNGG
NNNNNNNNNNNNNNNNNNNNNCC
GC
CG
5’ HA NotI digestion and processing
NotI cut site
5’...GCGGCCGC...3’
3’...CGCCGGCG...5’
Digestion product
5’GGCCGC...3’
3’CG...5’
5’ exonuclease acitivty
5’..3’
3’CG...5’
5’ HA
3’ HA
NotI
NotI
KanR-ColE1
20 bp spacer
PAM
Chr. I
5’ HA: 178043...178563 (521 bp)
3’ HA: 178564...179100 (537 bp)
Int.1
CCTCATGCAGCCCTCGTTAATAT
GGAGTACGTCGGGAGCAATTATA
Chr. IV
5’ HA: 358177...358664 (488 bp)
3’ HA: 358665...359206 (542 bp)
Int.2
TAAACGAGAAGTGACAGTGGTGG
ATTTGCTCTTCACTGTCACCACC
Chr. VI
5’ HA: 25790...26425 (636 bp)
3’ HA: 26426...26940 (515 bp)
Int.3
AGTCTTTTGAATCAACCGTAAGG
TCAGAAAACTTAGTTGGCATTCC
Chr. VII
5’ HA: 18698...19208 (511 bp)
3’ HA: 19209...19724 (516 bp)
Int.4
CCTCTGTTATCGTGCGAATGGAA
GGAGACAATAGCACGCTTACCTT
Chr. VIII
5’ HA: 202227...202751 (525 bp)
3’ HA: 202752...203265 (514 bp)
Int.5
GCAATTGAGGTACATATAAGAGG
CGTTAACTCCATGTATATTCTCC
Chr. IX
5’ HA: 337270...337771 (502 bp)
3’ HA: 337772...338361 (590 bp)
Int.6
ACGTATTTAAAGACAATACAAGG
TGCATAAATTTCTGTTATGTTCC
Chr. XI
5’ HA: 19786...20292 (507 bp)
3’ HA: 20293...20804 (512 bp)
Int.7
CCGCGGTAACAGTAGCATCACAC
GGCGCCATTGTCATCGTAGTGTG
Chr. XIII
5’ HA: 433128...433662 (535 bp)
3’ HA: 433663...434166 (504 bp)
Int.8
CTTTGCACAATGCATTACGTGGG
GAAACGTGTTACGTAATGCACCC
Chr. XV
5’ HA: 701610...702111 (502 bp)
3’ HA: 702112...702626 (515 bp)
Int.9
CCACTCATCTTGGCATCGCTGAC
GGTGAGTAGAACCGTAGCGACTG
Chr. XVI
5’ HA: 588839...589378 (540 bp)
3’ HA: 589379...589860 (582 bp)
Int.10
TACAACGCCAACCTAAAGAGTGG
ATGTTGCGGTTGGATTTCTCACC

Supporting Figure 2. MYT integration loci homology arm design. The MYT integration vector homology arms were designed around the gRNA target site at each locus (20 bp spacer; purple, PAM; blue), with the inside of each homology arm starting at the precise location that Cas9 is designed to cut. This design principle was chosen to promote efficient strand invasion of the chromosomal DNA into the integration cassette at the double strand break (DSB) and disrupt the gRNA target once the vector has integrated, thus preventing further cutting. The outside of the homology arms were designed to extend ~500 bp from the Cas9 cut site and terminate with a GC on the genome. The GC is then incorporated as part of a NotI site in the integration vector, so that after vector digestion with NotI, and transformation into yeast, the 5' ends with be chewed back by endogenous exonuclease activity, leaving only a residual 3' GC from the NotI site. As this sequence is also present on the genome, the homology arms will have perfect sequence complementarity during strand invasion to maximize recombination efficiency. Internal BsaI, BsmBI, BbsI, NotI, EcoRI, XbaI, SpeI, PstI, BglII, and XhoI sites were removed from the homology arms for compatibility with YTK and MYT cloning methods. These sites were removed using a single C-to-T point mutation to facilitate G-T base pairing during strand invasion to improve DNA pairing at the mismatch. Finally, the sequences inside of the homology arms were differentiated to prevent unwanted recombination between the vectors during multiplexed integration. gRNA spacers for all integration loci are listed in **Supporting Table 7**.

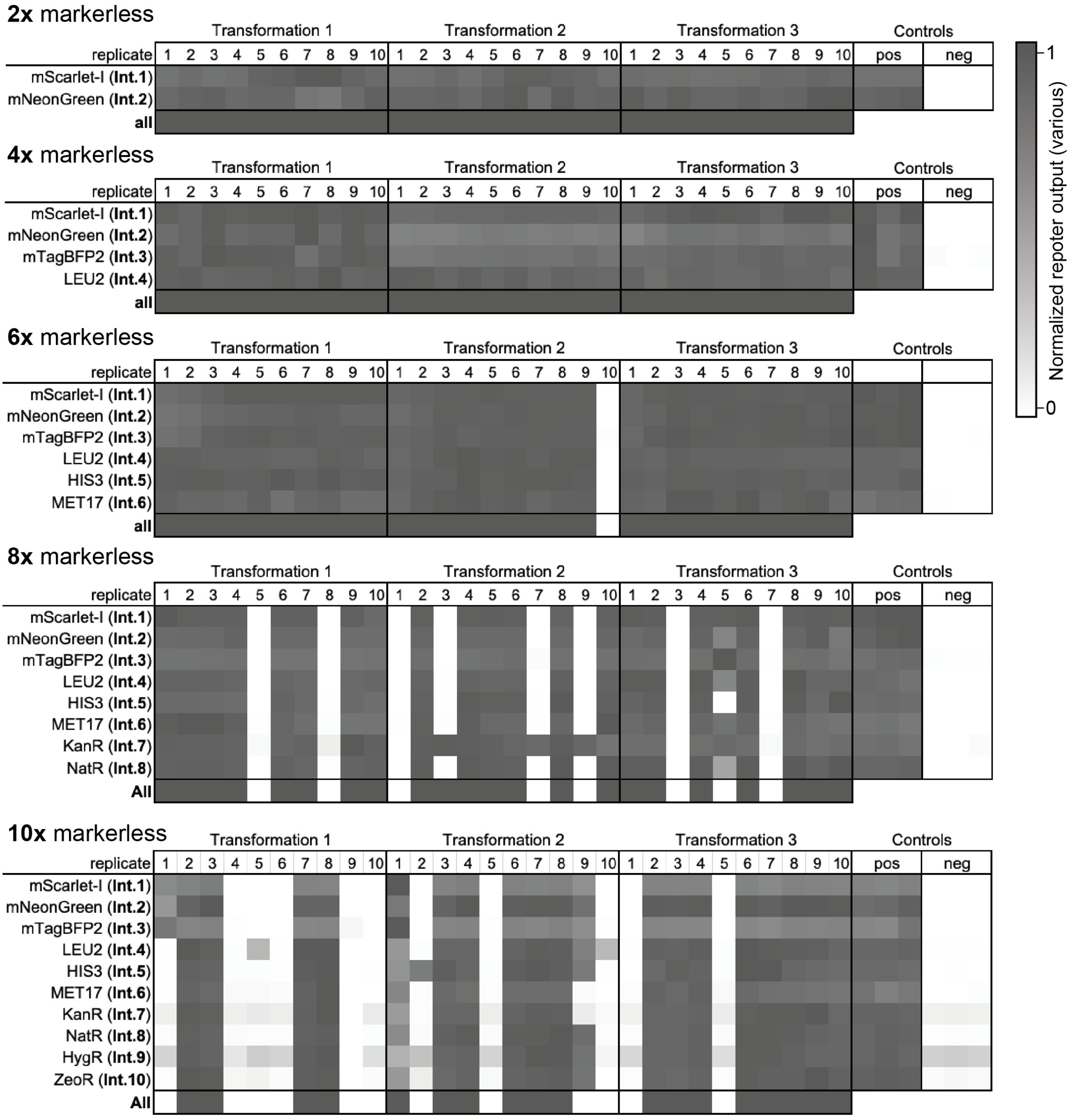

Supporting Figure 3. Individual reporter responses from multiplexed vector integration using markerless CRISPR-Cas9. Various outputs of between two and 10 reporters, showing individual reporter measurements of 10 colonies randomly selected from three independent transformations. Experimental measurements are either fluorescence as determined by flow cytometry or optical density at 700 nM as determined on a plate reader after 16 h growth in selective media. Data are normalized to the average maximum (1) and minimum (0) reporter responses for each reporter measurement. Reporter measurements are aggregated in "All" showing gray when all reporters are present and white when at least one reporter is absent.

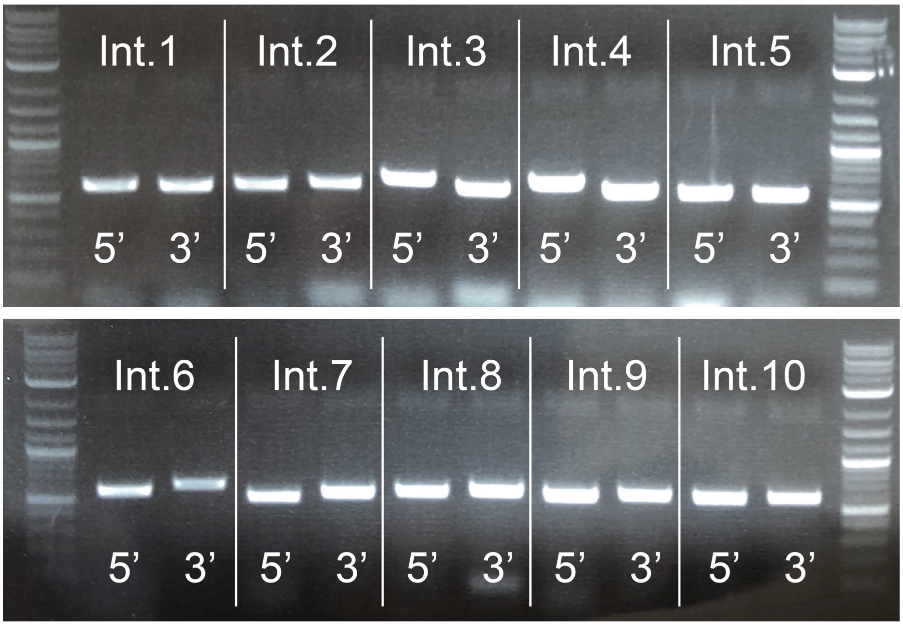

Supporting Figure 4. Colony PCR validation of a representative 10x markerless CRISPR-Cas9 strain. PCR amplification was performed across the 5’ and 3’ homology arms of each integration loci in a randomly chosen strain from the 10x reporter integration which showed all reporters were present. Positive bands, around 600 bp, are only possible with precise integration of the plasmid at their respective locus using a primer pair that targets the genome and a barcode sequence unique to the integration vector. For a list of primers used to validate plasmid integrations, see **Supporting Table 10**.

***8x** markerless

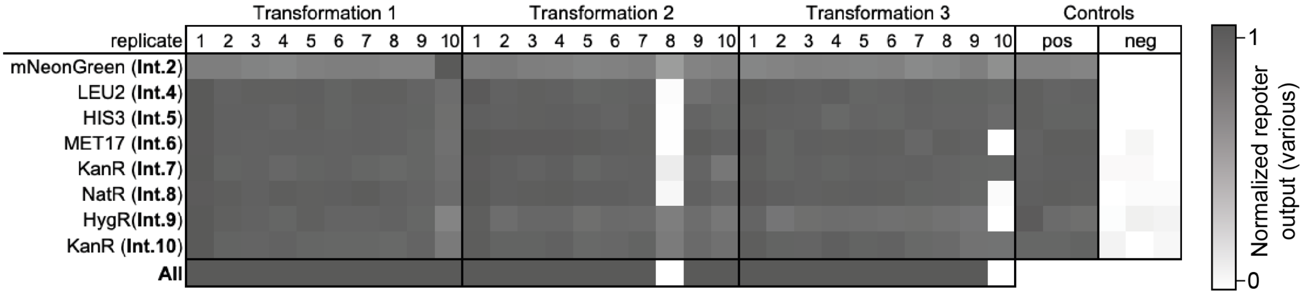

Supporting Figure 5. Individual reporter responses for select 8x vector integration using markerless CRISPR-Cas9. Various outputs of eight distinct reporters showing individual reporter measurements of 10 colonies randomly selected from three independent transformations. Experimental measurements are either fluorescence as determined by flow cytometry or optical density at 700 nM as determined on a plate reader after 16 h growth in selective media. Data are normalized to the average maximum (1) and minimum (0) reporter responses for each reporter measurement. Reporter measurements are aggregated in “All” showing gray when all reporters are present and white when at least one reporter is absent.

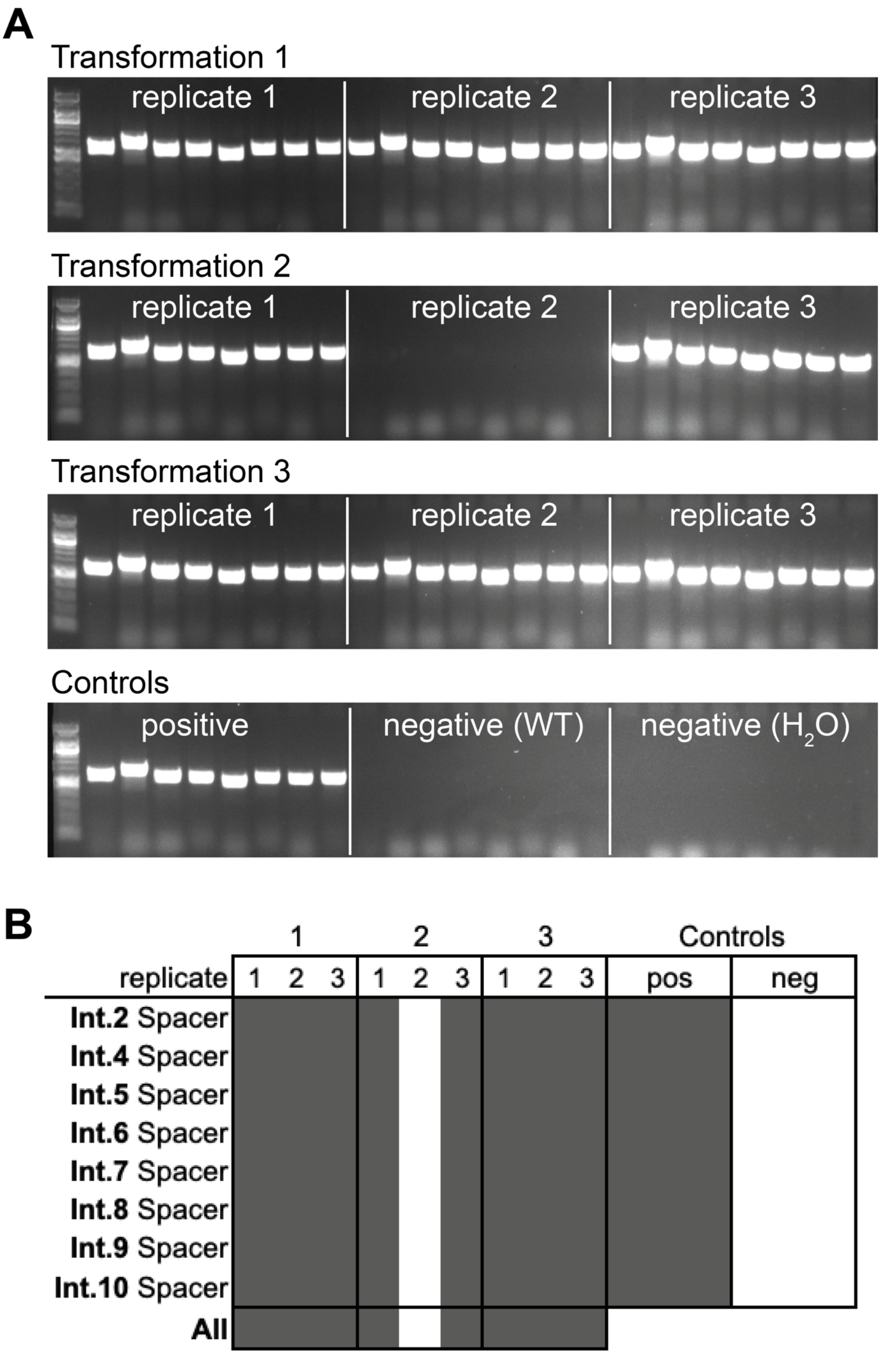

Supporting Figure 6. Colony PCR screening of select 8x spacer integration using markerless CRISPR-Cas9. (A) PCR amplification was performed across the 5’ homology arm of each integration loci in three randomly selection colonies from three independent transformations. Positive bands, around 600 bp, are only possible with precise integration of the vector at the respective locus using a primer pair that targets the genome and a barcode sequence unique to the integration vector. The positive condition is the previously validated strain containing the successful integration of all 10 vectors from Supporting Figure 4. The negative (WT) condition is unmodified BY4741 yeast. (B) Summary of the colony PCR data, showing gray for successful integration and white for unsuccessful integration. Data are aggregated in “All” showing gray when all integration plasmids are present and white when at least one plasmid is absent.

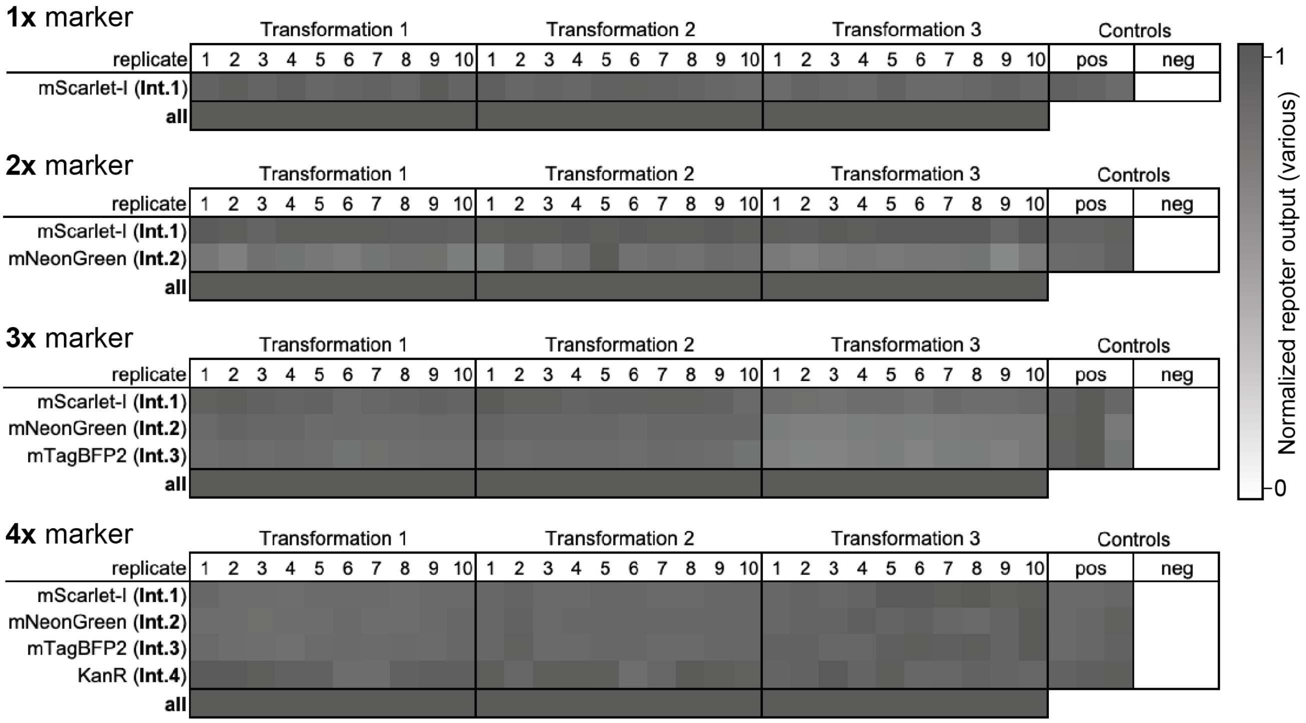

Supporting Figure 7. Fidelity of multiplexed vector integration using transient CRISPR-Cas9. Various outputs of between one and four reporters, showing individual reporter measurements of 10 colonies randomly selected from three independent transformations. Experimental measurements are either fluorescence as determined by flow cytometry or optical density at 700 nM as determined on a plate reader after 16 h growth in selective media. Data are normalized to the average maximum (1) and minimum (0) reporter responses for each reporter measurement. Reporter measurements are aggregated in “All” showing gray when all reporters are present and white when at least one reporter is absent.

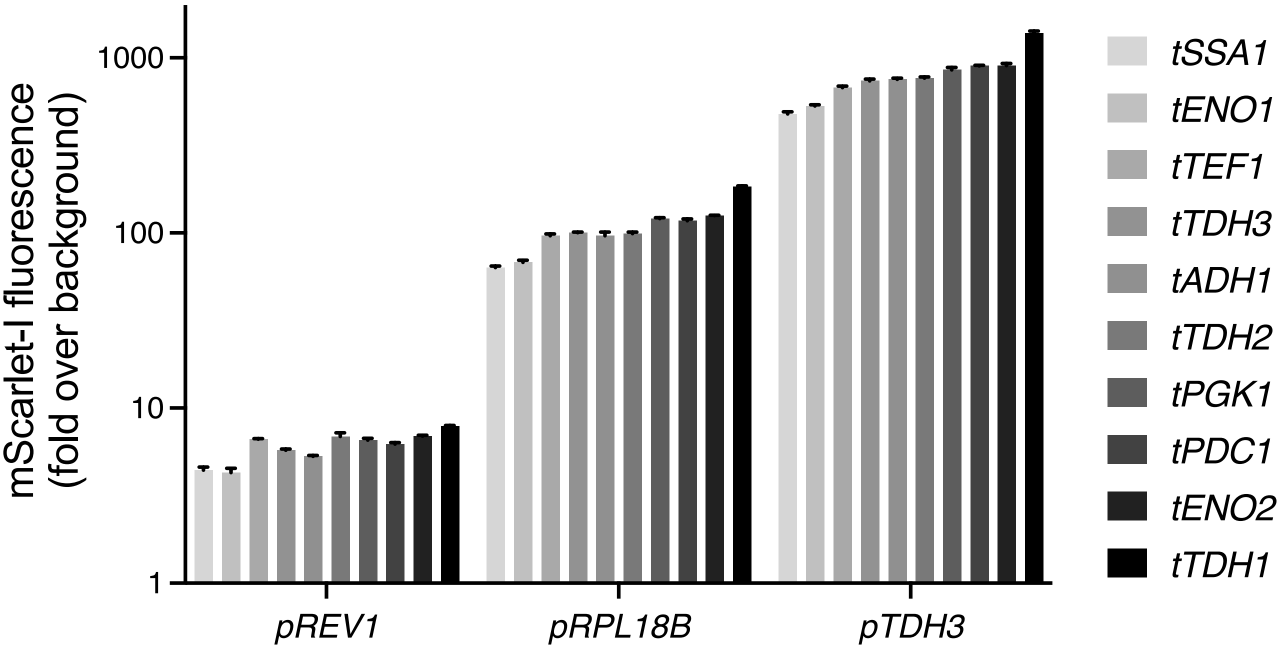

Supporting Figure 8. Extended terminator library characterization. The extended terminator library, consisting of 6 terminators from the original YTK and 4 additional promoters from MYT, were cloned behind mScarlet-I, each being driven by weak (*pREV1*), moderate (*pRPL18B*), and strong (*pTDH3*) promoter from YTK. Experimental measurements are mScarlet-I levels per cell determined by flow cytometry and shown as the mean ± SD from three biological replicates.

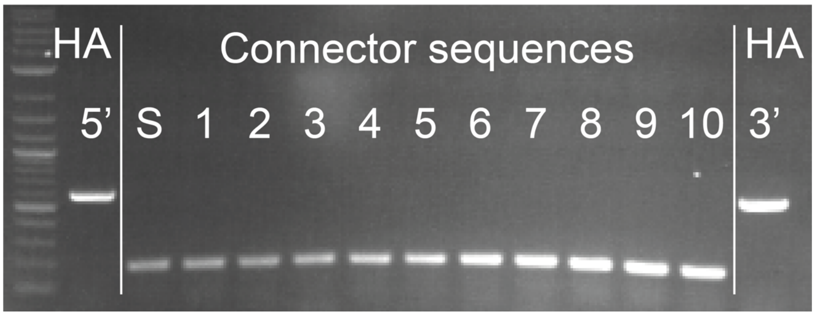

Supporting Figure 9. Colony PCR validation of a representative 10x gap repair assembly strain. PCR amplification was performed across the connector sequences and across the 5’ and 3’ homology arms of each integration loci in a randomly chosen strain from the 10x reporter gap repair which showed all reporters were present. Positive bands are only possible with precise gap repair assembly of each cassette into the genome at the desired locus (Int.1) using unique barcodes on each Level 1 assembly cassette and integration vector. For a list of primers used to validate gap repair assembly, see **Supporting Table 11**.

CTGA, CCAA, GATG, GTTC, GGTA, AAGT, **TAGA**, **ACGA**, **CTCC**, **AGGG**, AGCA
Estimated ligation fidelity: **99 %**

	CTGA	TCAG	CCAA	TTGG	GATG	CATC	GTTC	GAAC	GGTA	TACC	AAGT	ACTT	TAGA	TCTA	ACGA	TCGT	CTCC	GGAG	AGGG	CCCT	AGCA	TGCT
CTGA	0	349	0	0	0	0	0	0	0	0	0	0	0	0	0	0	0	0	0	0	0	0
TCAG	349	0	0	0	0	0	0	0	0	0	0	0	0	0	0	0	0	0	0	0	0	0
CCAA	0	0	0	194	0	0	0	0	0	0	0	0	0	0	0	0	0	0	0	0	0	0
TTGG	0	0	194	0	0	0	0	0	0	0	0	0	0	0	0	0	0	0	0	0	0	0
GATG	0	0	0	0	0	193	0	0	0	0	0	0	0	0	0	0	0	0	0	0	0	0
CATC	0	0	0	0	193	0	0	0	0	0	0	0	0	0	0	0	0	0	0	0	0	0
GTTC	0	0	0	0	0	0	1	270	0	0	0	0	0	0	0	0	0	0	0	0	0	0
GAAC	0	0	0	0	0	0	270	1	0	0	0	0	0	0	0	0	0	0	0	0	0	0
GGTA	0	0	0	0	0	0	0	0	0	232	0	0	0	0	0	0	0	0	0	0	0	0
TACC	0	0	0	0	0	0	0	0	232	0	0	0	0	0	0	0	0	0	0	0	0	0
AAGT	0	0	0	0	0	0	0	0	0	0	0	329	0	0	0	0	0	0	0	0	0	0
ACTT	0	0	0	0	0	0	0	0	0	0	329	0	0	0	0	0	0	0	0	0	0	0
TAGA	0	0	0	0	0	0	0	0	0	0	0	0	0	299	0	0	0	0	0	0	0	0
TCTA	0	0	0	0	0	0	0	0	0	0	0	0	299	0	0	0	0	0	0	0	0	0
ACGA	0	0	0	0	0	0	0	0	0	0	0	0	0	0	0	353	0	0	0	0	0	0
TCGT	0	0	0	0	0	0	0	0	0	0	0	0	0	0	353	0	0	0	0	0	0	0
CTCC	0	0	0	0	0	0	0	0	0	0	0	0	0	0	0	0	0	195	0	0	0	0
GGAG	0	0	0	0	0	0	0	0	0	0	0	0	0	0	0	0	195	0	0	0	0	0
AGGG	0	0	0	0	0	0	0	0	0	0	0	0	0	0	0	0	0	0	0	215	0	0
CCCT	0	0	0	0	0	0	0	0	0	0	0	0	0	0	0	0	0	0	215	0	0	0
AGCA	0	0	0	0	0	0	0	0	0	0	0	0	0	0	0	0	0	0	0	0	0	239
TGCT	0	0	0	0	0	0	0	0	0	0	0	0	0	0	0	0	0	0	0	0	239	1

Legend

- good Watson-Crick pair
- poor Watson-Crick pair
- high-count mismatch
- modest mismatch
- trace mismatch

Supporting Figure 10. Estimated ligation fidelity of MYT Level 2 assembly overhangs. Four new overhangs (bold) were added to the existing YTK overhangs using the NEB GetSet tool with BsmBI-v2 42-16 cycling settings. Estimate ligation fidelity and ligation frequency matrix generated by NEB Ligase Fidelity calculator v1.0.

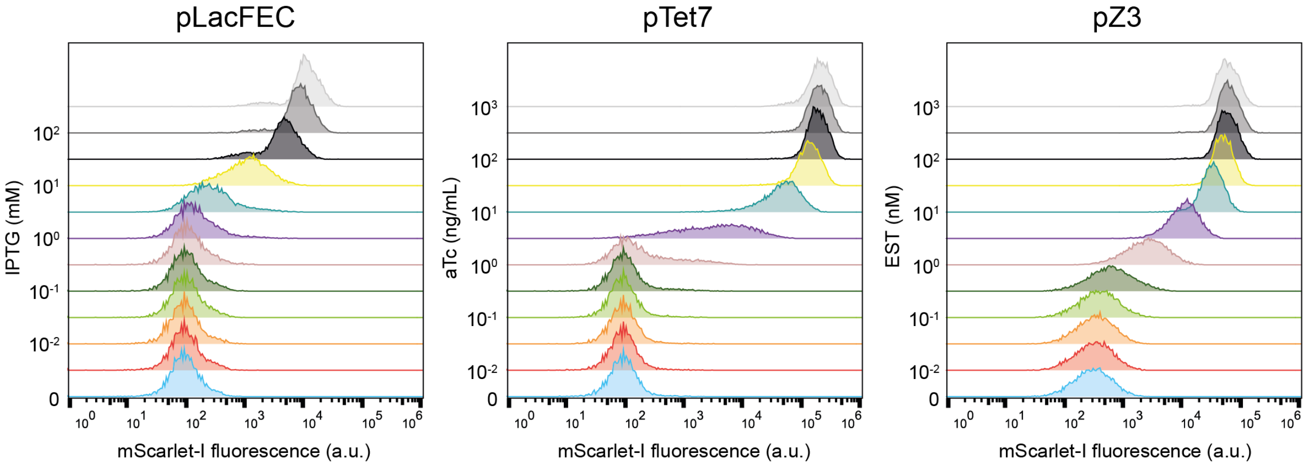

Supporting Figure 11. Flow cytometry histograms of the inducible promoter systems. Representative histograms of the data in Figure 5A, showing population heterogeneity across the varying ligand concentrations. Histograms were generated in FlowJo, showing 10,000 cells from a single replicate for each condition.

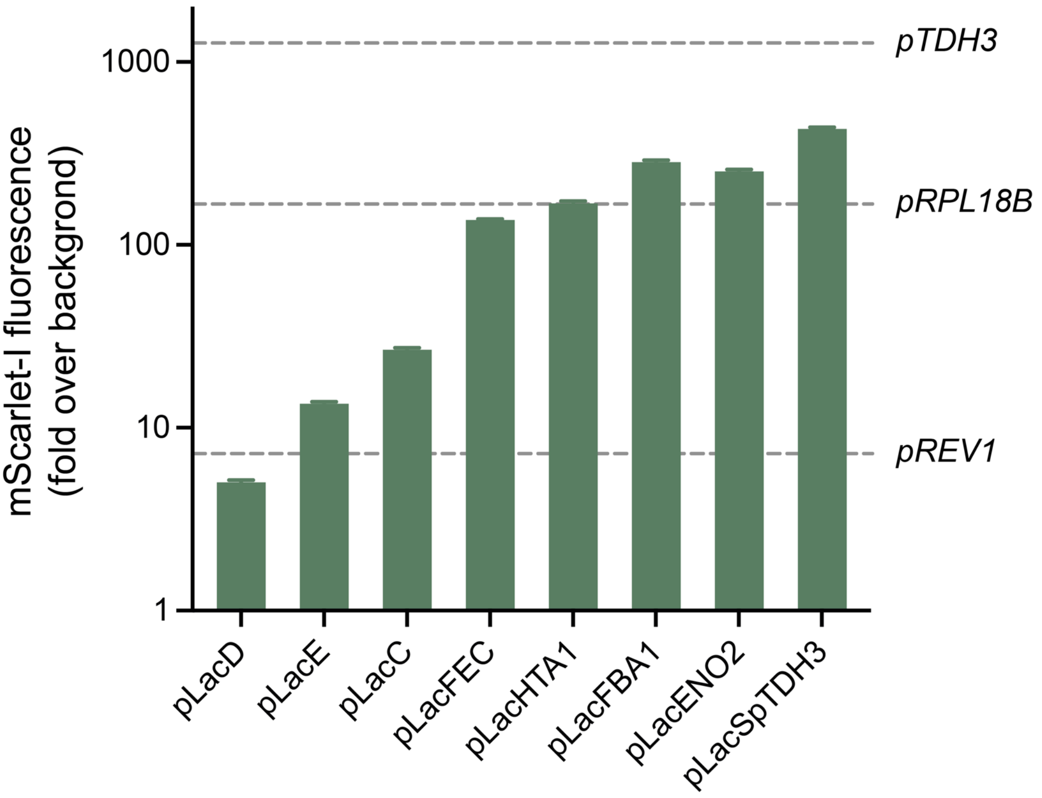

Supporting Figure 12. LacI promoter expression without LacI. Promoters from the LacI promoter library driving the expression of mScarlet-I in the absence of LacI. Experimental measurements are mScarlet-I levels per cell as determined by flow cytometry and shown as the mean ± SD from three biological replicates and normalized to an untransformed control. mScarlet-I fluorescence from the *TDH3*, *RPL18B*, *REV1* promoters from YTK are present as a reference of relative expression.

Supporting Table 1. MYT integration loci design criteria. Design principles were applied to the reference S288C genome, with certain criteria checked against other commonly used laboratory strains of *Saccharomyces cerevisiae* (see **Supporting Table 2**). All exceptions to design principles are in respect to the S288C genome.

**Design principle**	**Exception**
Integration loci are native sites within the *Saccharomyces cerevisiae* genome and do not require the pre-installation of DNA for use	
Integration loci reside in intergenic regions > 1000 bp from the start and > 500 bp from the end of neighboring ORFs (taken from the center of the landing pad)	
Neighboring genes are non-essential	Int.2 locus: 1905 bp from start codon of essential gene *PSA1*.
Homology arms are only seen once in the genome and have no sequence similarity to any other location	
Integration loci have high sequence similarity between common lab strains	
A gRNA target site with a predicted high on-target score and maximum off-target score sits at the core of the landing pad for CRISPR-Cas9 mediated integration	

Supporting Table 2. Integration vector yeast strain compatibility. Sequence similarity of the 5' and 3' homology arms to the genomes of commonly used lab strains of *Saccharomyces cerevisiae*. Number is percentage similarity of combined 5' and 3' homology arms of each integration vector to the yeast strain, highlighting a 100 % match in gray. An Asterix (*) denotes sequences which have an alternate gRNA spacer to that listed in **Supporting Table 7**.

CEN.PK Int.4 locus alternative gRNA spacer sequence = TTCTATTCGCACGATAACAG.

Locus	BY4741	CEN.PK	FL100	W303	YPH499
**Int.1**	100	99	100	100	100
**Int.2**	100	100	100	98	100
**Int.3**	100	100	100	100	100
**Int.4**	100	97*	99	100	100
**Int.5**	100	100	100	100	100
**Int.6**	100	100	99	100	100
**Int.7**	100	100	100	100	100
**Int.8**	100	100	97	100	100
**Int.9**	100	100	100	100	100
**Int.10**	100	100	100	100	100

Supporting Table 3. gRNA-tRNA array primer design. gRNA-tRNA primers can be designed manually using the below primer design template. “N” represents the forward DNA sequence and “R” represents the reverse complement DNA sequence for the 20 bp gRNA spacer sequences. The first spacer is pink, the second spacer is green, and the third spacer is purple. The letters in bold are the nucleotides that bind the template in the pMYT095/096 plasmid for amplifying the gRNA-tRNA fragments in the PCR reaction. The letters highlighted in gray are the BsaI generated overhangs which ligate with the CRISPR-Cas9 plasmid and between gRNA-tRNA fragments. The regular letters at the 5’ of each primer are the BsaI recognition sequences and pre- and post-recognition sequences. For the second and third spacers, the split of the 20 bp does not need to be evenly distributed. The NEBridge SplitSet tool can be used to design optimal over hangs between gRNA-tRNA fragments (ligasefidelity.neb.com).

**Primer**	Sequence 5’ > 3’ (grey highlight = BsaI overhang)
**Frag 1 FP**	TTTGGTCTCACGCANNNNNNNNNNNNNNNNNNNN**GTTTTAGAGCTAGAAATAGCAAGTTA**
**Frag 1 RP**	TTTGGTCTCARRRRRRRRRRRRR**TGCGCAAGCCGGGAATCG**
**Frag 2 FP**	TTTGGTCTCANNNNNNNNNNNNN**GTTTTAGAGCTAGAAATAGCAAGTTA**
**Frag 2 RP**	TTTGGTCTCARRRRRRRRRRRRR**TGCGCAAGCCGGGAATCG**
**Frag 3 FP**	TTTGGTCTCANNNNNNNNNNNNN**GTTTTAGAGCTAGAAATAGCAAGTTA**
**Frag 3 RP**	TTTGGTCTCAGGAT**TGCGCAAGCCGGGAATCG**

Alternatively, and by far the best approach, is to use an online tool, such as Benchling, to design the primers. In the Benchling Golden Gate wizard, set pMYT095/096 as the backbone (using BsaI), and then set the Cas9 scaffold-tRNA template as an insert after highlighting the sequence and choosing “selection”. The 20 bp gRNA spacer sequences can then be introduced by adding a “spacer” before each insert. Benchling will then design the primer sequence automatically. The following settings in the wizard should design primers with the properties in the table above; Pre recognition site length, 3 bp; Pre-recognition site bases, TTT; Min Tm for binding region, 57 °C; Min length for binding region, 18 bp; Max Tm diff for primer pairs, 20 °C; Type IIS restriction enzyme, BsaI. Otherwise, the primers can be edited manually after assembly by setting a binding region of 26 nt and 18 nt for the forward and reverse primer, respectively, and setting the pre-recognition sequence as “TTT” (the post-recognition sequence does not matter).

Here is a link to an example 1x gRNA-tRNA array targeting the Int.1 locus from Figure 2C:
https://benchling.com/s/seq-RTsp7JX0dkYnN781IBHi?m=slm-MWvbxJ8cpdfP1YXfqqYV

Here is a link to an example 8x gRNA-tRNA array targeting the loci in the select *8x array from Figure 2E:
https://benchling.com/s/seq-PsVG8W3dtnAHCL7z1Awr?m=slm-UfRw6xizKjw8i6OuIhP4

Supporting Table 4. Organization of Level 1 assembly cassettes in a Level 2 multigene assembly.

No. of TUs/ position	1	2	3	4	5	6	7	8	9	10
2x	**pMYT 039**	**pMYT 040**								
3x	**pMYT 039**	**pMYT 041**	**pMYT 042**							
4x	**pMYT 039**	**pMYT 041**	**pMYT 043**	**pMYT 044**						
5x	**pMYT 039**	**pMYT 041**	**pMYT 043**	**pMYT 045**	**pMYT 046**					
6x	**pMYT 039**	**pMYT 041**	**pMYT 043**	**pMYT 045**	**pMYT 047**	**pMYT 048**				
7x	**pMYT 039**	**pMYT 041**	**pMYT 043**	**pMYT 045**	**pMYT 047**	**pMYT 049**	**pMYT 050**			
8x	**pMYT 039**	**pMYT 041**	**pMYT 043**	**pMYT 045**	**pMYT 047**	**pMYT 049**	**pMYT 051**	**pMYT 052**		
9x	**pMYT 039**	**pMYT 041**	**pMYT 043**	**pMYT 045**	**pMYT 047**	**pMYT 049**	**pMYT 051**	**pMYT 053**	**pMYT 054**	
10x	**pMYT 039**	**pMYT 041**	**pMYT 043**	**pMYT 045**	**pMYT 047**	**pMYT 049**	**pMYT 051**	**pMYT 053**	**pMYT 055**	**pMYT 056**

Supporting Table 5. Organization of Level 1 assembly spacers in a Level 2 multigene assembly.

No. of TUs/ position	1	2	3	4	5	6	7	8	9	10
2x	**pMYT 057**	**pMYT 058**								
3x	**pMYT 057**	**pMYT 059**	**pMYT 060**							
4x	**pMYT 057**	**pMYT 059**	**pMYT 061**	**pMYT 062**						
5x	**pMYT 057**	**pMYT 059**	**pMYT 061**	**pMYT 063**	**pMYT 064**					
6x	**pMYT 057**	**pMYT 059**	**pMYT 061**	**pMYT 063**	**pMYT 065**	**pMYT 066**				
7x	**pMYT 057**	**pMYT 059**	**pMYT 061**	**pMYT 063**	**pMYT 065**	**pMYT 067**	**pMYT 068**			
8x	**pMYT 057**	**pMYT 059**	**pMYT 061**	**pMYT 063**	**pMYT 065**	**pMYT 067**	**pMYT 069**	**pMYT 070**		
9x	**pMYT 057**	**pMYT 059**	**pMYT 061**	**pMYT 063**	**pMYT 065**	**pMYT 067**	**pMYT 069**	**pMYT 071**	**pMYT 072**	
10x	**pMYT 057**	**pMYT 059**	**pMYT 061**	**pMYT 063**	**pMYT 065**	**pMYT 067**	**pMYT 069**	**pMYT 071**	**pMYT 073**	**pMYT 074**

Supporting Table 6. Yeast transformation and plating guidelines. This is a rough guide for transformations and will vary between individuals, labs, and experiments. We recommend following these guidelines to begin, and then optimize from there. The DNA amounts here are in fmol to account for differences in plasmid size. For quick reference, 50 fmol of an integration plasmid with a single gene is ~200 ng, 200 fmol of an assembly cassette is ~500 ng, 50 fmol of transient CRISPR-Cas9 plasmid is ~200 ng, 500 fmol of CRISPR-Cas9 vector is ~400 ng. For routine transformations of single integration vectors (with or without CRISPR-Cas9) or multiplexed integration using transient CRISPR-Cas9, the quick reference amounts shown above can be used. For gap repair and multiplexed markerless integration using CRISPR-Cas9, we recommend using the amounts shown in the table below to achieve high efficiency/fidelity. Resuspension volume is the amount of sterile H_2O to resuspend the cells in the final step of the yeast transformation. Plating 100 µL of the resuspended cells will lead to around ~100 colonies per plate (lower for multiplexed experiments >6 integration vectors).

**Condition**	**Integration vector (each)**	**Assembly cassette (each)**	**CRISPR-Cas9 plasmid (each)**	**Resuspension volume**	**Cells to plate**
**Integration using marker without CRISPR-Cas9**					
1x int. vector	50 fmol	N/A	N/A	1000 µL	100 µL
**Gap repair using marker and transient CRISPR-Cas9**					
1x cassette	50 fmol	200 fmol	50 fmol	1000 µL	100 µL
2x cassette	50 fmol	200 fmol	50 fmol	500 µL	100 µL
3x cassette	50 fmol	200 fmol	50 fmol	400 µL	100 µL
4x cassette	50 fmol	200 fmol	50 fmol	300 µL	100 µL
5x cassette	50 fmol	200 fmol	50 fmol	200 µL	100 µL
6x cassette	50 fmol	200 fmol	50 fmol	100 µL	100 µL
7x cassette	50 fmol	200 fmol	50 fmol	100 µL	100 µL
8x cassette	50 fmol	200 fmol	50 fmol	100 µL	100 µL
9x cassette	50 fmol	200 fmol	50 fmol	100 µL	100 µL
10x cassette	50 fmol	200 fmol	50 fmol	100 µL	100 µL
**Integration using transient CRISPR-Cas9**					
1x int. vector	50 fmol	N/A	50 fmol	1000 µL	dilute 1:10 and plate 100 µL
2x int. vector	50 fmol	N/A	50 fmol	1000 µL	dilute 1:2 and plate 100 µL
3x int. vector	50 fmol	N/A	50 fmol	1000 µL	100 µL
4x int. vector	50 fmol	N/A	50 fmol	500 µL	100 µL
**Markerless integration using CRISPR-Cas9**					
1x int. vector	500 fmol	N/A	50 fmol	1000 µL	100 µL
2x int. vector	500 fmol	N/A	50 fmol	500 µL	100 µL
3x int. vector	500 fmol	N/A	50 fmol	400 µL	100 µL
4x int. vector	500 fmol	N/A	50 fmol	300 µL	100 µL
5x int. vector	500 fmol	N/A	50 fmol	200 µL	100 µL
6x int. vector	500 fmol	N/A	50 fmol	100 µL	100 µL
7x int. vector	500 fmol	N/A	50 fmol	100 µL	100 µL
8x int. vector	500 fmol	N/A	50 fmol	100 µL	100 µL
9x int. vector	500 fmol	N/A	50 fmol	100 µL	100 µL
10x int. vector	500 fmol	N/A	50 fmol	100 µL	100 µL

Supporting Table 7. CRISPR-Cas9 spacers.

Spacer description	Spacer sequence 5’ > 3’
Int.1 locus	ATATTAACGAGGGCTGCATG
Int.2 locus	TAAACGAGAAGTGACAGTGG
Int.3 locus	AGTCTTTTGAATCAACCGTA
Int.4 locus	TTCCATTCGCACGATAACAG
Int.5 locus	GCAATTGAGGTACATATAAG
Int.6 locus	ACGTATTTAAAGACAATACA
Int.7 locus	GTGTGATGCTACTGTTACCG
Int.8 locus	CTTTGCACAATGCATTACGT
Int.9 locus	GTCAGCGATGCCAAGATGAG
Int.10 locus	TACAACGCCAACCTAAAGAG
Int.1 spacer	GTCTCAATATGTTAACCCGT
Int.2 spacer	AGATGAGCGCAGGGACACCG
Int.3 spacer	AGGTCAAGTACATTACGCTA
Int.4 spacer	CGAGCATGACACTATCTGCG
Int.5 spacer	ACTTGGCTGGGTCTGCTAGG
Int.6 spacer	CACTTGAGGATACCCCGACC
Int.7 spacer	CACCGTAAAGTCCTCCGCCG
Int.8 spacer	TATGAACAACGCAAGGATTG
Int.9 spacer	GCGATATAAACAGAGTAACG
Int.10 spacer	GCTGATTACACTTGATCGTG
Spacer cassette 1	CGCATCTGATGCTACCGTGG
Spacer cassette 2	TGGTGTTAAGGGGTCCCCTG
Spacer cassette 3	CGGACCTGAGTCGACCAAGG
Spacer cassette 4	CCGACCGCAGAACTTACGAG
Spacer cassette 5	TCCCCGGTTACCTCTCCACG
Spacer cassette 6	ATGGTCGTAAAAGCCTCCCA
Spacer cassette 7	ATGCTCACTACTCCAATCGG
Spacer cassette 8	ACGGATACGTAGAATTATCG
Spacer cassette 9	CGTATTTGGTGGTAGCATGG
Spacer cassette 10	CCGTTCAGTCTCGTCCAAGG

Supporting Table 8. Multiplex Yeast Toolkit plasmids. All plasmids are available from Addgene (addgene.org) individually and as a kit containing all plasmids, supplied in a 96-well format (Kit 1000000229).

Plasmid name	Part Type	Plasmid Description	Antibiotic resistance	Addgene ID	Plate position
**pMYT001**	2	pZ3	Chloramphenicol	180654	A1
**pMYT002**	2	pLacD	Chloramphenicol	180655	A2
**pMYT003**	2	pLacE	Chloramphenicol	180656	A3
**pMYT004**	2	pLacC	Chloramphenicol	180657	A4
**pMYT005**	2	pLacFEC	Chloramphenicol	180658	A5
**pMYT006**	2	pLacHTA1	Chloramphenicol	203322	A6
**pMYT007**	2	pLacFBA1	Chloramphenicol	180660	A7
**pMYT008**	2	pLacENO2	Chloramphenicol	180661	A8
**pMYT009**	2	pLacSpTDH3	Chloramphenicol	180662	A9
**pMYT010**	2	pTet2	Chloramphenicol	180663	A10
**pMYT011**	2	pTet3	Chloramphenicol	180664	A11
**pMYT012**	2	pTet4	Chloramphenicol	180665	A12
**pMYT013**	2	pTet5	Chloramphenicol	180666	B1
**pMYT014**	2	pTet6	Chloramphenicol	180667	B2
**pMYT015**	2	pTet7	Chloramphenicol	180668	B3
**pMYT016**	3	Z3EV	Chloramphenicol	180669	B4
**pMYT017**	3	LacI	Chloramphenicol	180670	B5
**pMYT018**	3	rtTA	Chloramphenicol	180671	B6
**pMYT019**	3	mScarlet-I	Chloramphenicol	180672	B7
**pMYT020**	3	mNeonGreen	Chloramphenicol	180673	B8
**pMYT021**	3	mTagBFP2	Chloramphenicol	180674	B9
**pMYT022**	4a	mScarlet-I	Chloramphenicol	180675	B10
**pMYT023**	4a	mNeonGreen	Chloramphenicol	180676	B11
**pMYT024**	4a	mTagBFP2	Chloramphenicol	180677	B12
**pMYT025**	4	*tTDH3*	Chloramphenicol	180678	C1
**pMYT026**	4	*tTEF1*	Chloramphenicol	180679	C2
**pMYT027**	4	*tTDH2*	Chloramphenicol	180680	C3
**pMYT028**	4	*tPDC1*	Chloramphenicol	180681	C4
**pMYT029**	N/A	*URA3*	Chloramphenicol	180682	C5
**pMYT030**	N/A	*LEU2*	Chloramphenicol	180683	C6
**pMYT031**	N/A	*HIS3*	Chloramphenicol	180684	C7
**pMYT032**	N/A	*TRP1*	Chloramphenicol	180685	C8
**pMYT033**	N/A	*MET17*	Chloramphenicol	180686	C9
**pMYT034**	N/A	*LYS2*	Chloramphenicol	180687	C10
**pMYT035**	N/A	KanR	Chloramphenicol	180688	C11
**pMYT036**	N/A	NatR	Chloramphenicol	180689	C12
**pMYT037**	N/A	HygR	Chloramphenicol	180690	D1
**pMYT038**	N/A	ZeoR	Chloramphenicol	180691	D2
**pMYT039**	N/A	S/1 Assembly Cassette	Ampicillin	180692	D3
**pMYT040**	N/A	1/E Assembly Cassette	Ampicillin	180693	D4
**pMYT041**	N/A	1/2 Assembly Cassette	Ampicillin	180694	D5
**pMYT042**	N/A	2/E Assembly Cassette	Ampicillin	180695	D6
**pMYT043**	N/A	2/3 Assembly Cassette	Ampicillin	180696	D7
**pMYT044**	N/A	3/E Assembly Cassette	Ampicillin	180697	D8
**pMYT045**	N/A	3/4 Assembly Cassette	Ampicillin	180698	D9
**pMYT046**	N/A	4/E Assembly Cassette	Ampicillin	180699	D10
**pMYT047**	N/A	4/5 Assembly Cassette	Ampicillin	180700	D11

**pMYT048**	N/A	5/E Assembly Cassette	Ampicillin	180701	D12
**pMYT049**	N/A	5/6 Assembly Cassette	Ampicillin	180702	E1
**pMYT050**	N/A	6/E Assembly Cassette	Ampicillin	180703	E2
**pMYT051**	N/A	6/7 Assembly Cassette	Ampicillin	180704	E3
**pMYT052**	N/A	7/E Assembly Cassette	Ampicillin	180705	E4
**pMYT053**	N/A	7/8 Assembly Cassette	Ampicillin	180706	E5
**pMYT054**	N/A	8/E Assembly Cassette	Ampicillin	180707	E6
**pMYT055**	N/A	8/9 Assembly Cassette	Ampicillin	180708	E7
**pMYT056**	N/A	9/E Assembly Cassette	Ampicillin	180709	E8
**pMYT057**	N/A	S/1 Spacer Cassette	Ampicillin	180710	E9
**pMYT058**	N/A	1/E Spacer Cassette	Ampicillin	180711	E10
**pMYT059**	N/A	1/2 Spacer Cassette	Ampicillin	180712	E11
**pMYT060**	N/A	2/E Spacer Cassette	Ampicillin	180713	E12
**pMYT061**	N/A	2/3 Spacer Cassette	Ampicillin	180714	F1
**pMYT062**	N/A	3/E Spacer Cassette	Ampicillin	180715	F2
**pMYT063**	N/A	3/4 Spacer Cassette	Ampicillin	180716	F3
**pMYT064**	N/A	4/E Spacer Cassette	Ampicillin	180717	F4
**pMYT065**	N/A	4/5 Spacer Cassette	Ampicillin	180718	F5
**pMYT066**	N/A	5/E Spacer Cassette	Ampicillin	180719	F6
**pMYT067**	N/A	5/6 Spacer Cassette	Ampicillin	180720	F7
**pMYT068**	N/A	6/E Spacer Cassette	Ampicillin	180721	F8
**pMYT069**	N/A	6/7 Spacer Cassette	Ampicillin	180722	F9
**pMYT070**	N/A	7/E Spacer Cassette	Ampicillin	180723	F10
**pMYT071**	N/A	7/8 Spacer Cassette	Ampicillin	180724	F11
**pMYT072**	N/A	8/E Spacer Cassette	Ampicillin	180725	F12
**pMYT073**	N/A	8/9 Spacer Cassette	Ampicillin	180726	G1
**pMYT074**	N/A	9/E Spacer Cassette	Ampicillin	180727	G2
**pMYT075**	N/A	Int.1 Vector	Kanamycin	180728	G3
**pMYT076**	N/A	Int.2 Vector	Kanamycin	180729	G4
**pMYT077**	N/A	Int.3 Vector	Kanamycin	180730	G5
**pMYT078**	N/A	Int.4 Vector	Kanamycin	180731	G6
**pMYT079**	N/A	Int.5 Vector	Kanamycin	180732	G7
**pMYT080**	N/A	Int.6 Vector	Kanamycin	180733	G8
**pMYT081**	N/A	Int.7 Vector	Kanamycin	180734	G9
**pMYT082**	N/A	Int.8 Vector	Kanamycin	198864	G10
**pMYT083**	N/A	Int.9 Vector	Kanamycin	180736	G11
**pMYT084**	N/A	Int.10 Vector	Kanamycin	180737	G12
**pMYT085**	N/A	Int.1 Spacer	Kanamycin	180738	H1
**pMYT086**	N/A	Int.2 Spacer	Kanamycin	180739	H2
**pMYT087**	N/A	Int.3 Spacer	Kanamycin	180740	H3
**pMYT088**	N/A	Int.4 Spacer	Kanamycin	180741	H4
**pMYT089**	N/A	Int.5 Spacer	Kanamycin	180742	H5
**pMYT090**	N/A	Int.6 Spacer	Kanamycin	180743	H6
**pMYT091**	N/A	Int.7 Spacer	Kanamycin	180744	H7
**pMYT092**	N/A	Int.8 Spacer	Kanamycin	198865	H8
**pMYT093**	N/A	Int.9 Spacer	Kanamycin	180746	H9
**pMYT094**	N/A	Int.10 Spacer	Kanamycin	180747	H10
**pMYT095**	N/A	URA3 CRISPR Vector	Spectinomycin	198866	H11
**pMYT096**	N/A	Transient CRISPR Vector	Spectinomycin	198867	H12

Supporting Table 9. CRISPR-Cas9 primers used in this study.

Oligo description	Oligo sequence 5' > 3'
**gRNA array sequencing FP**	AAACAACTGTGTGATCCTTGAG
**gRNA array sequencing RP**	ACACATGTATCTCAGATATCTCATTATATC
**1x Int.1 gRNA frag FP**	TTTGGTCTCCCGCAATATTAACGAGGGCTGCATGGTTTTAGAGCTAGAAATAGCAAGTTA
**1x Int.2 gRNA frag FP**	TTTGGTCTCCCGCATAAACGAGAAGTGACAGTGGGTTTTAGAGCTAGAAATAGCAAGTTA
**1x Int.3 gRNA frag FP**	TTTGGTCTCGCGCAAGTCTTTTGAATCAACCGTAGTTTTAGAGCTAGAAATAGCAAGTTA
**1x Int.4 gRNA frag FP**	TTTGGTCTCGCGCATTCCATTCGCACGATAACAGGTTTTAGAGCTAGAAATAGCAAGTTA
**1x Int.5 gRNA frag FP**	TTTGGTCTCGCGCAGCAATTGAGGTACATATAAGGTTTTAGAGCTAGAAATAGCAAGTTA
**1x Int.6 gRNA frag FP**	TTTGGTCTCCCGCAACGTATTTAAAGACAATACAGTTTTAGAGCTAGAAATAGCAAGTTA
**1x Int.7 gRNA frag FP**	TTTGGTCTCCCGCAGTGTGATGCTACTGTTACCGGTTTTAGAGCTAGAAATAGCAAGTTA
**1x Int.8 gRNA frag FP**	TTTGGTCTCCCGCACTTTGCACAATGCATTACGTGTTTTAGAGCTAGAAATAGCAAGTTA
**1x Int.9 gRNA frag FP**	TTTGGTCTCGCGCAGTCAGCGATGCCAAGATGAGGTTTTAGAGCTAGAAATAGCAAGTTA
**1x Int.10 gRNA frag FP**	TTTGGTCTCCCGCATACAACGCCAACCTAAAGAGGTTTTAGAGCTAGAAATAGCAAGTTA
**All 1x gRNA frag RP**	TTTGGTCTCCGGATTGCGCAAGCCGGGAATCG
**10x gRNA array frag 1 FP**	TTTGGTCTCCCGCAATATTAACGAGGGCTGCATGGTTTTAGAGCTAGAAATAGCAAGTTA
**10x gRNA array frag 1 RP**	TTTGGTCTCCCTCGTTTATGCGCAAGCCGGGAATCG
**10x gRNA array frag 2 FP**	TTTGGTCTCCCGAGAAGTGACAGTGGGTTTTAGAGCTAGAAATAGCAAGTTA
**10x gRNA array frag 2 RP**	TTTGGTCTCCAAAAGACTTGCGCAAGCCGGGAATCG
**10x gRNA array frag 3 FP**	TTTGGTCTCGTTTTGAATCAACCGTAGTTTTAGAGCTAGAAATAGCAAGTTA
**10x gRNA array frag 3 RP**	TTTGGTCTCGGAATGGAATGCGCAAGCCGGGAATCG
**10x gRNA array frag 4 FP**	TTTGGTCTCCATTCGCACGATAACAGGTTTTAGAGCTAGAAATAGCAAGTTA
**10x gRNA array frag 4 RP**	TTTGGTCTCCTATGTACCTCAATTGCTGCGCAAGCCGGGAATCG
**10x gRNA array frag 5 FP**	TTTGGTCTCCCATATAAGGTTTTAGAGCTAGAAATAGCAAGTTA
**10x gRNA array frag 5 RP**	TTTGGTCTCCGTCTTTAAATACGTTGCGCAAGCCGGGAATCG
**10x gRNA array frag 6 FP**	TTTGGTCTCGAGACAATACAGTTTTAGAGCTAGAAATAGCAAGTTA
**10x gRNA array frag 6 RP**	TTTGGTCTCGAGTAGCATCACACTGCGCAAGCCGGGAATCG
**10x gRNA array frag 7 FP**	TTTGGTCTCGTACTGTTACCGGTTTTAGAGCTAGAAATAGCAAGTTA
**10x gRNA array frag 7 RP**	TTTGGTCTCGTTGTGCAAAGTGCGCAAGCCGGGAATCG
**10x gRNA array frag 8 FP**	TTTGGTCTCCACAATGCATTACGTGTTTTAGAGCTAGAAATAGCAAGTTA
**10x gRNA array frag 8 RP**	TTTGGTCTCCTGGCATCGCTGACTGCGCAAGCCGGGAATCG
**10x gRNA array frag 9 FP**	TTTGGTCTCCGCCAAGATGAGGTTTTAGAGCTAGAAATAGCAAGTTA
**10x gRNA array frag 9 RP**	TTTGGTCTCCAGGTTGGCGTTGTATGCGCAAGCCGGGAATCG
**10x gRNA array frag 10 FP**	TTTGGTCTCCACCTAAAGAGGTTTTAGAGCTAGAAATAGCAAGTTA
**10x gRNA array frag 10 RP**	TTTGGTCTCCGGATTGCGCAAGCCGGGAATCG
***8x gRNA array frag 1 FP**	TTTGGTCTCCCGCATAAACGAGAAGTGACAGTGGGTTTTAGAGCTAGAAATAGCAAGTTA
***8x gRNA array frag 1 RP**	TTTGGTCTCCCGTGCGAATGGAATGCGCAAGCCGGGAATCG
***8x gRNA array frag 2 FP**	TTTGGTCTCGCACGATAACAGGTTTTAGAGCTAGAAATAGCAAGTTA
***8x gRNA array frag 2 RP**	TTTGGTCTCGACCTCAATTGCTGCGCAAGCCGGGAATCG
***8x gRNA array frag 3 FP**	TTTGGTCTCGAGGTACATATAAGGTTTTAGAGCTAGAAATAGCAAGTTA
***8x gRNA array frag 3 RP**	TTTGGTCTCGCTTTAAATACGTTGCGCAAGCCGGGAATCG
***8x gRNA array frag 4 FP**	TTTGGTCTCGAAAGACAATACAGTTTTAGAGCTAGAAATAGCAAGTTA
***8x gRNA array frag 4 RP**	TTTGGTCTCGAGTAGCATCACACTGCGCAAGCCGGGAATCG
***8x gRNA array frag 5 FP**	TTTGGTCTCCTACTGTTACCGGTTTTAGAGCTAGAAATAGCAAGTTA
***8x gRNA array frag 5 RP**	TTTGGTCTCCCATTGTGCAAAGTGCGCAAGCCGGGAATCG
***8x gRNA array frag 6 FP**	TTTGGTCTCGAATGCATTACGTGTTTTAGAGCTAGAAATAGCAAGTTA
***8x gRNA array frag 6 RP**	TTTGGTCTCGTGGCATCGCTGACTGCGCAAGCCGGGAATCG
***8x gRNA array frag 7 FP**	TTTGGTCTCGGCCAAGATGAGGTTTTAGAGCTAGAAATAGCAAGTTA
***8x gRNA array frag 7 RP**	TTTGGTCTCGGTTGGCGTTGTATGCGCAAGCCGGGAATCG

***8x gRNA array frag 8 FP**	TTTGGTCTCGCAACCTAAAGAGGTTTTAGAGCTAGAAATAGCAAGTTA
***8x gRNA array frag 8 RP**	TTTGGTCTCGGGATTGCGCAAGCCGGGAATCG
**Transient CRISPR check FP**	TTCGAGGATAGGGAAATGATC
**Transient CRISPR check RP**	GCCCAATTCTTTGATACCCTC

Supporting Table 10. Colony PCR primers for verification of vector integration.

Primer description	Sequence 5’ > 3’	Tm (˚C)	Product length (bp)
Int.1 5' HA FP	CCGAAGTTCATATACGAATGC	60.3	596
Int.1 5' HA RP	AGTCGTGGATGTGATGGGTA	62.2	
Int.1 3' HA FP	GTCTAAGCACGAAGGAAACG	60.8	593
Int.1 3' HA RP	GCATTGCAAGATATTGAATAACTG	60.7	
Int.2 5' HA FP	CATAAATGCAACATAGGCAGC	61.3	600
Int.2 5' HA RP	GGGATAGGGGAGACAAGAAC	60.6	
Int.2 3' HA FP	CCATGCCGTTACTTTGGACT	63.1	623
Int.2 3' HA RP	AACATTGACCCATTTCTTATGC	60.9	
Int.3 5' HA FP	TTATTAATTCATGGAAGGCATTG	60.8	688
Int.3 5' HA RP	GGAGTCGTGAGTTCTGAGCA	62.6	
Int.3 3' HA FP	AGCATCTTGTTACCGACCAA	61.4	622
Int.3 3' HA RP	TGGCATGTATCCATTGGG	62.1	
Int.4 5' HA FP	TTAGATGTAGCTGTAAAGAAACTTGC	60.2	680
Int.4 5' HA RP	TGTCACTCTCACTCCAAGCC	62.4	
Int.4 3' HA FP	CGACACGGACCTGAGTTAGT	61.1	608
Int.4 3' HA RP	GGGAATCGGAGAGCATTTAG	61.8	
Int.5 5' HA FP	ATGATCACTGAGCAAATTAAAGC	60.5	595
Int.5 5' HA RP	AGATGCCCACTTCTGATAGG	60	
Int.5 3' HA FP	GATTCATTCCTCTGTCTGCG	61.7	587
Int.5 3' HA RP	GGAATAGCCCTCTTCGTTG	60.5	
Int.6 5' HA FP	CTGTTTCATCCCTCCCATC	61.2	599
Int.6 5' HA RP	CTACTAGGGATTGGGGTGTG	60.2	
Int.6 3' HA FP	ATCCCCTAATCTCCAAACCG	63.1	689
Int.6 3' HA RP	TCTTATTTATTTAGTTTACGTACCCTTCC	61.1	
Int.7 5' HA FP	TCTTTGTTCGAAGGAGAACAG	60.3	550
Int.7 5' HA RP	GGCACGGTATGTTTTAAGGC	62.4	
Int.7 3' HA FP	CGGGTAAGAGGTGGAGGATT	63.3	586
Int.7 3' HA RP	GTTATGTTTTATCTTTGGTCAGGG	60.8	
Int.8 5' HA FP	GAAAGGCAAACCTAAACTAACTTATG	60.6	601
Int.8 5' HA RP	GTGCCTCTGCCTTCAATCAT	63.4	
Int.8 3' HA FP	CGCTAGGAAAGATACACGGC	62.9	625
Int.8 3' HA RP	GTGTTATAAGTTCATTTCGCCC	60.5	
Int.9 5' HA FP	GGCAACTACTCTCAAAGGTGG	61.9	596
Int.9 5' HA RP	TTTGTTGGACAAGGGAATCG	64	
Int.9 3' HA FP	ACCCGAAGGAAATGTGTAGA	60.3	615
Int.9 3' HA RP	GCATAATATCCTCTTGAGCATTG	60.8	
Int.10 5' HA FP	TGTCAGAAACGAAATGTGGG	62.8	610
Int.10 5' HA RP	CGTAGCGTAGGTGAGGAAGA	61.3	
Int.10 3' HA FP	ATGACCGCTAGAATTACCCC	61	618
Int.10 3' HA RP	CACGGATGTCTTGATGAATCTC	62.7	

Supporting Table 11. Colony PCR primers for verification of gap repair assembly.

**Primer description**	**Sequence 5’ > 3’**	**Tm (˚C)**	**Product length (bp)**
ConS FP	CTGTGGTCAAAAGTGCGTTG	63.6	187
ConS RP	GACCTAGCCACATTTCCAAG	60.4	
Con1 FP	CTAATCGGTCCTCGTTTGCT	62.4	187
Con1 RP	TTCTGTAGTTCATCGGGTCC	60.8	
Con2 FP	GAACCGATTAGGGGCATGTA	62.8	187
Con2 RP	GAGCTGGGAGATTGTGTCTG	61.8	
Con3 FP	CTCATAACCTGGACCGAATG	61.8	187
Con3 RP	GTGGCTTTACCTCGTATTGC	61.2	
Con4 FP	GAATCTTTACTCCCAAGCGA	60.2	187
Con4 RP	GGAATACGAGACACCAAGCA	62	
Con5 FP	ATGGACGAAATGTTTCACGA	62.2	187
Con5 RP	CCTTGATCCTTCGCTACACC	62.8	
Con6 FP	GCAGACACAGTAAGACACGG	60.3	187
Con6 RP	ACATGGTGACTCGTCCTTCT	60.5	
Con7 FP	CGGTCGGACTATTCATTGTG	62.2	187
Con7 RP	AGCACCTTTCCCACGTAAAC	62.1	
Con8 FP	CAGTCCATCCGATCGTACAT	61.7	187
Con8 RP	GTCCGTCATTGCATCTAGGT	60.9	
Con9 FP	TGACTAACTGTGCCAAATCG	60.6	187
Con9 RP	ACTGGGCAGGAAGAAAGTGT	62	
ConE FP	GGACCAGCATATTCATGTCA	60.3	187
ConE RP	CAGACGAGCACCAAATACCC	63.6	

Multiplex Yeast Toolkit plasmid architecture

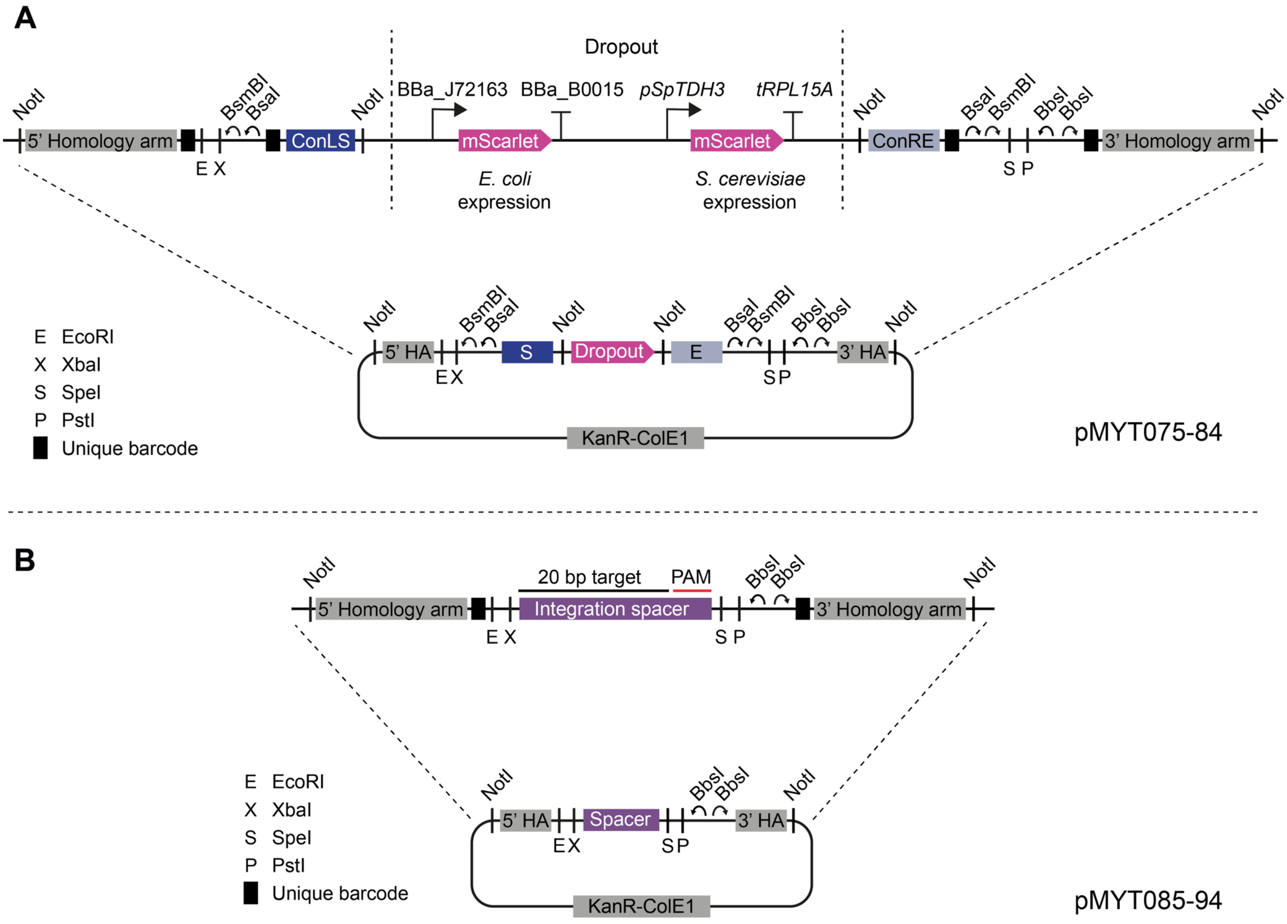

Architecture of integration vectors and integration spacers. (A) Integration vector architecture (pMYT075-084). All integration vectors in the MYT kit are provided without a marker. Selectable markers can be added using a BbsI Golden Gate assembly reaction (see section below). The BsaI and BsmBI sites for assembling Level 1 TUs and Level 2 multigene cassettes are as found in YTK. NotI sites are flanking the genomic integration homology arms and inside the connector (Con) sequences. For assembly of multigene cassettes in yeast using gap repair, the integration vector is digested at all four NotI sites to release the genome homology arms and connector sequence homology. Assembly of Level 1 TUs or Level 2 multigene cassettes by Golden Gate assembly results in the loss of the internal NotI sites, which are then used to linearize the plasmid for integration. The dropout contains a dual cassette for expression of mScarlet in *E. coli* and yeast for screening assembled plasmids using the loss of red fluorescence. Unique barcode sequences are included inside the genome homology arms and outside of the connector sequences for validating correct plasmid integration and gap repair assembly, respectively. BioBrick formatting is included, as in YTK, for additional cloning flexibility. DNA sequences between restriction enzyme sites are unique for each integration vector to avoid of unwanted recombination between integration vectors in yeast. (B) Integration spacer architecture (pMYT085-094). Spacers consists of a unique sequence for each integration locus containing an addressable CRISPR-Cas9 target. For a list of CRISPR-Cas9 spacers, see **Supporting Table 7**. All integration spacers in the MYT kit are provided without a marker. Selectable markers can be added using a BbsI Golden Gate assembly reaction. NotI sites are flanking the genome integration homology arms to linearize the plasmid prior to transformation.

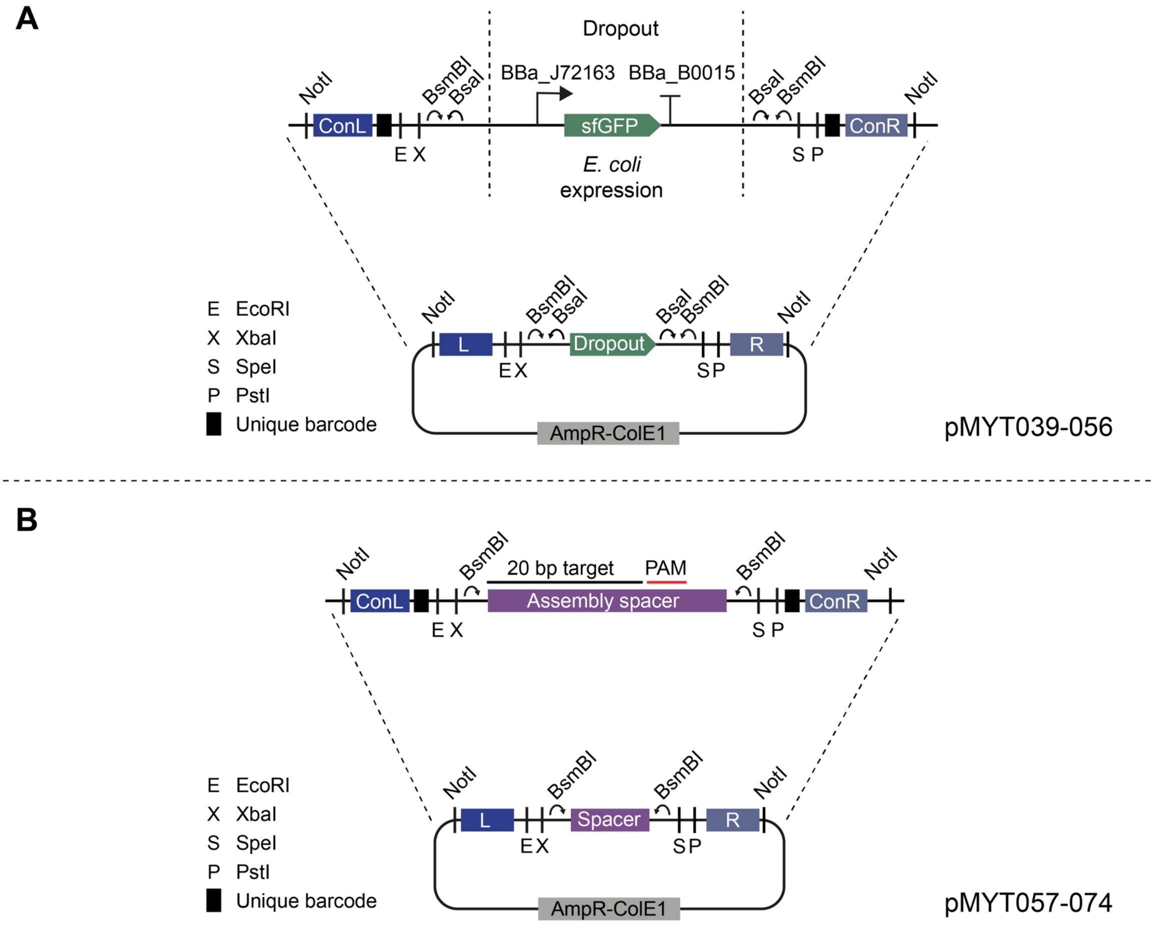

Architecture of assembly cassettes and spacer cassettes. (A) Assembly cassette architecture (pMYT039-056). The BsaI and BsmBI sites for assembling Level 1 TUs and Level 2 multigene cassettes are as found in YTK, with additional BsmBI overhangs designed for assemblies greater than six TUs. The dropout contains a cassette for expression of sfGFP in *E. coli* for screening Level 1 assemblies using the loss of green fluorescence. The entire cassette is flanked by connector (Con) sequences, as found in YTK, with additional NotI sites included for linearizing the cassette for gap repair assembly in yeast (see section below). Unique 20 bp barcodes are included next to the Con sequences for validating gap repair assembly by PCR. These barcodes are lost in multigene cassettes using BsmBI Golden Gate assembly to reduce sequence similarity between multigene cassettes. The BioBrick pre- and suffix were included from YTK, moving their position inside of the connector sequences for compatibility with gap repair assembly. DNA sequences between restriction enzyme sites are unique for each cassette to avoid unwanted recombination between cloning sites in yeast. (B) Spacer cassette architecture (pMYT057-074). Spacer cassettes are a unique sequence for each position in a multigene assembly containing an addressable CRISPR-Cas9 target. For a list of CRISPR-Cas9 spacers, see **Supporting Table 7**. Spacers can be assembled in a Level 2 multigene cassette using a BsmBI Golden Gate assembly or gap repair assembly in yeast.

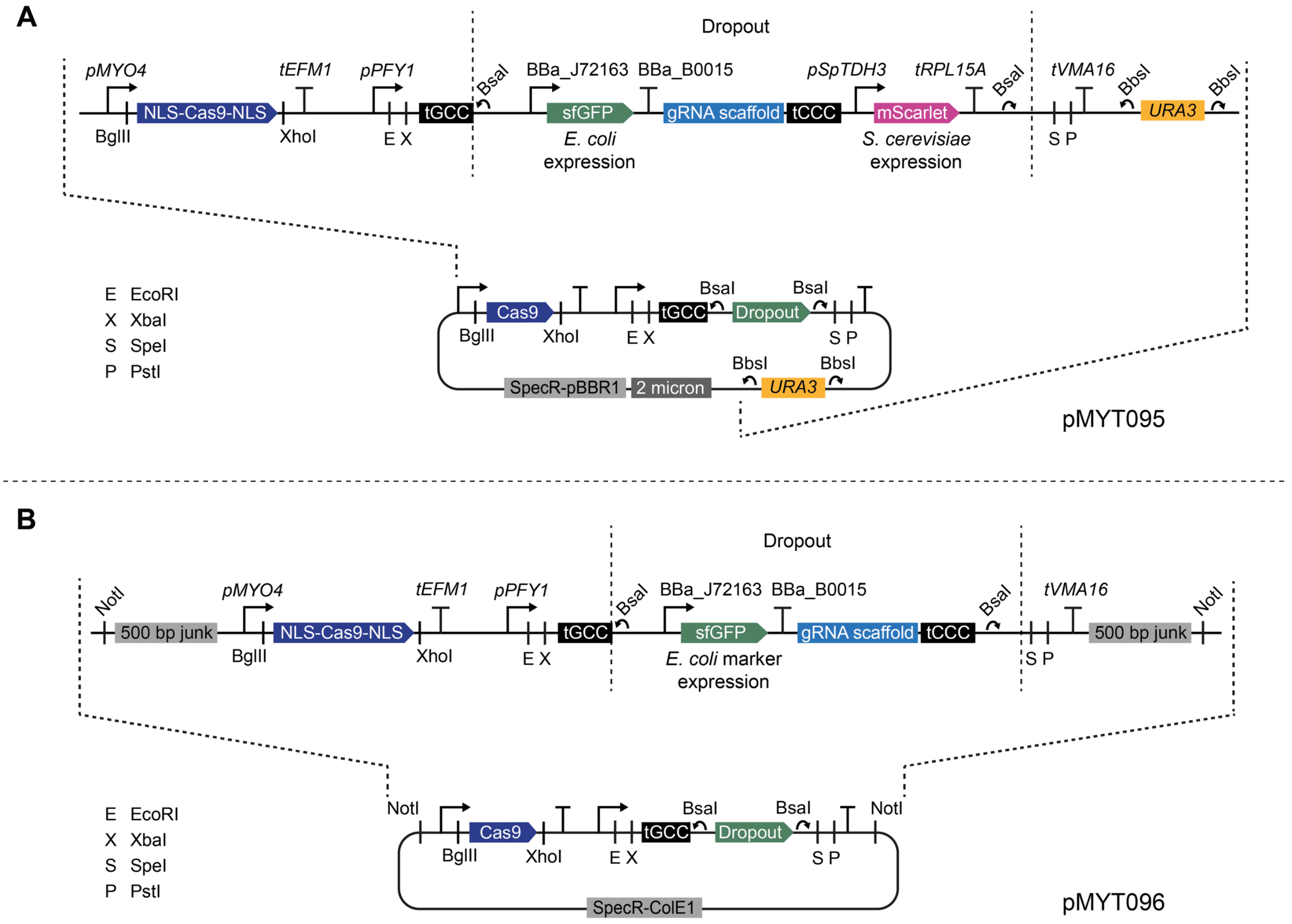

Architecture of the CRISPR-Cas9 plasmids. (A) pMYT095 plasmid architecture. pMYT095 comes pre-installed with the *URA3* selection marker, which can be swapped out for any of the other selectable markers included in MYT using a BbsI Golden Gate reaction (see section below). The dropout contains a dual cassette for expression of sfGFP in *E. coli* and mScarlet in yeast for screening assembled gRNA arrays using the loss of green or red fluorescence, respectively, and the template for creating gRNA fragments by PCR. The dropout is flanked by BsaI sites for cloning gRNA arrays (see section below). The Cas9 CDS is flanked by a BglII and XhoI site for exchanging the Cas protein for alternatives, if desired. A modified BioBrick pre- and suffix was included up and downstream of the gRNA array for manipulating arrays after assembly for additional flexibility. The entire plasmid was designed with DNA sequences (promoters, terminators, replicons etc.) which are not present in markerless integration vectors built with the YTK and MYT systems to prevent unwanted recombination between the CRISPR-Cas9 plasmid and integration vectors during transformation. Promoter and terminator sequences not found in YTK or MYT were taken from Chen et al. (2020). All other orthogonal sequences were designed in this work. (B) pMYT096 plasmid architecture. The entire Cas9 and gRNA expression cassette is flanked by 500 bp of biologically inert DNA and NotI sites to linearize the plasmid before transformation. The dropout contains a cassette for expression of sfGFP in *E. coli* for screening assembled gRNA arrays using the loss of green fluorescence and the template for creating gRNA fragments by PCR. Restriction enzymes sites either side of the Cas9 CDS and gRNA arrays are as seen in pMYT095.

Multiplex Yeast Toolkit plasmid assembly methods

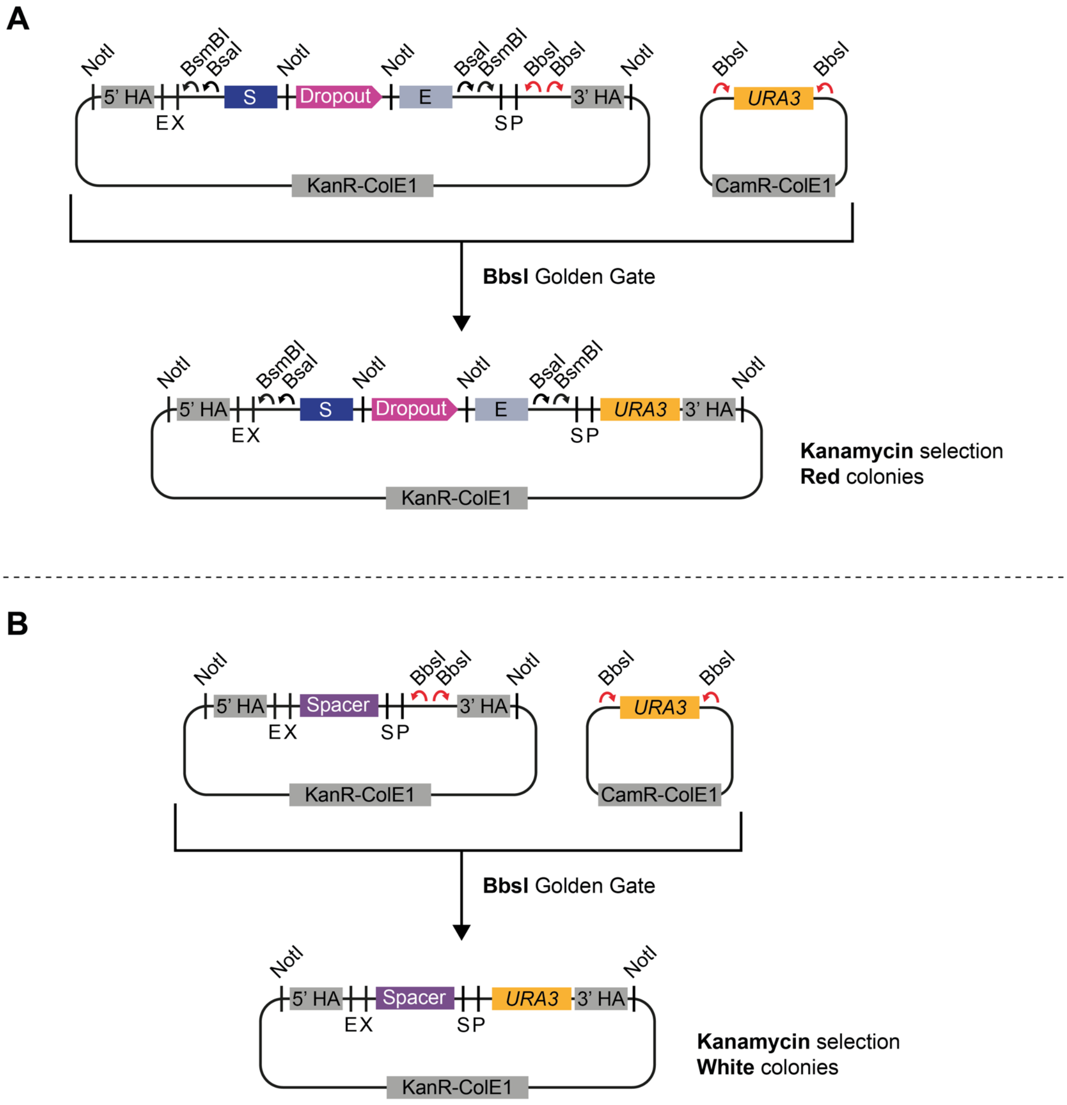

Assembly of a selectable marker into a markerless integration vector and spacer. (A) Addition of a selectable marker (pMYT029-038) into a markerless integration vector (pMYT075-pMYT084). Selectable markers are cloned into markerless integration vectors using a BbsI Golden Gate assembly and transformed into *E. coli*. As the assembly does not change the fluorescence marker on the plasmid, we recommend adding an excess of the selectable marker plasmid to drive the assembly to completion. Using 12.5 fmol of plasmid backbone and 50 fmol of the insert works well for us in a standard Golden Gate reaction and usually leads to all resulting clones being correct. All colonies should have red fluorescence. Use BsaI or BsmBI digestion to validate assembly. Selectable markers can be cloned into markerless integration vectors where TUs have already been assembled, however, BbsI sites cannot be present in any of the assembled sequences (YTK and MYT part sequences do not contain BbsI sites). (B) Addition of a selectable marker into a markerless integration spacer (pMYT085-094). Same assembly method as above. All colonies should be non-fluorescent (white). Use NotI digestion to validate assembly.

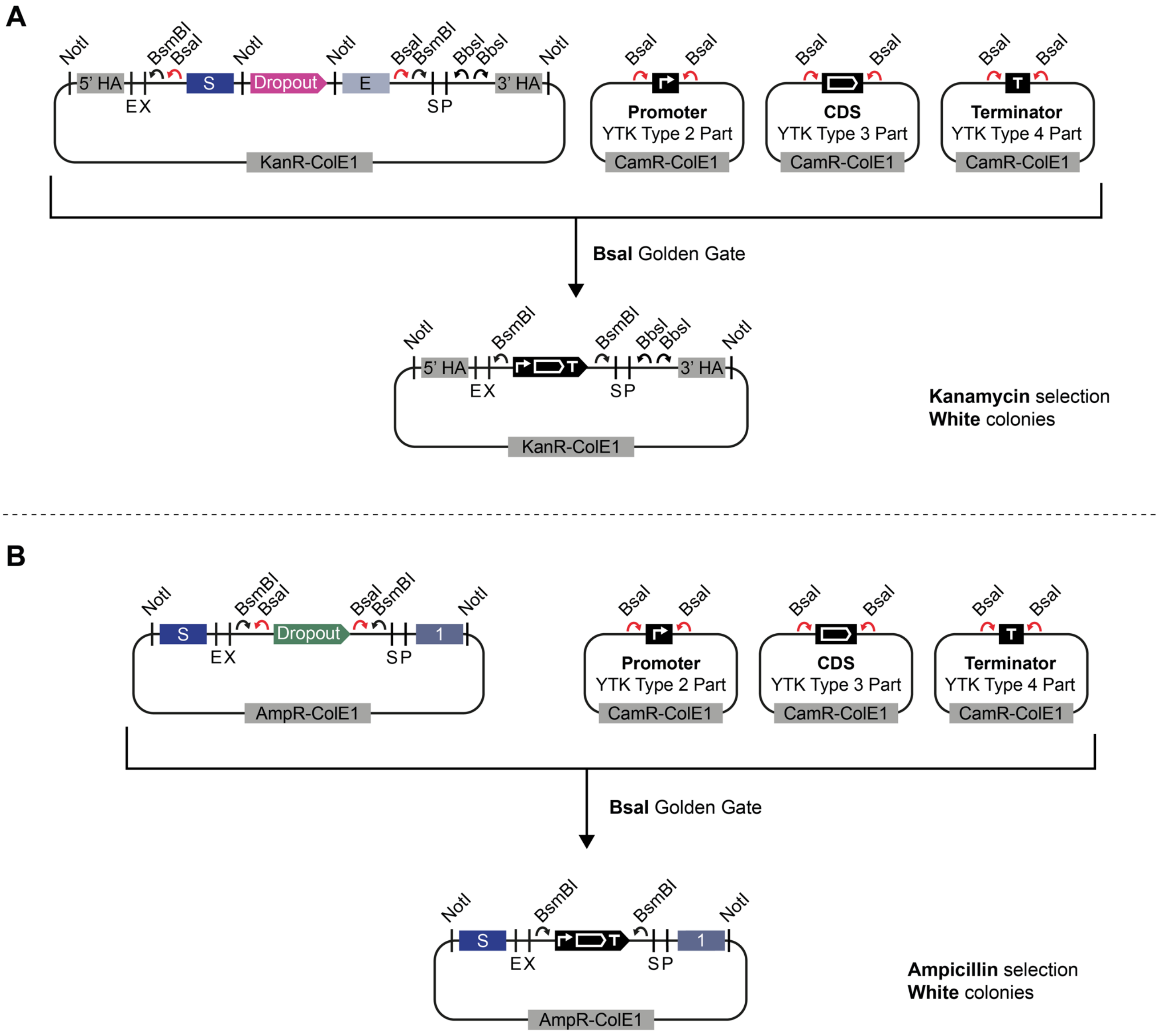

Assembly of transcriptional units. (A) Assembly of a Level 1 transcriptional unit into an integration vector (pMYT075-084 or derivatives). Part plasmids, formatted in the YTK standard (Type 2 through Type 4), are assembled into an integration vector in a BsaI Golden Gate reaction, transformed into *E. coli*, and selected on kanamycin medium. Plasmids are prepped from non-fluorescent, white colonies (loss of red fluorescence) and correct assemblies are validated using a NotI or BsmBI digestion. (B) Assembly of a Level 1 transcriptional unit into an assembly cassette. Part plasmids, formatted in the YTK standard (Type 2 through Type 4), are assembled into an assembly cassette in a BsaI Golden Gate reaction and transformed into *E. coli* and selected on ampicillin medium. Plasmids are prepped from non-fluorescent, white colonies (loss of green fluorescence) and correct assemblies are validated using a NotI or BsmBI digestion.

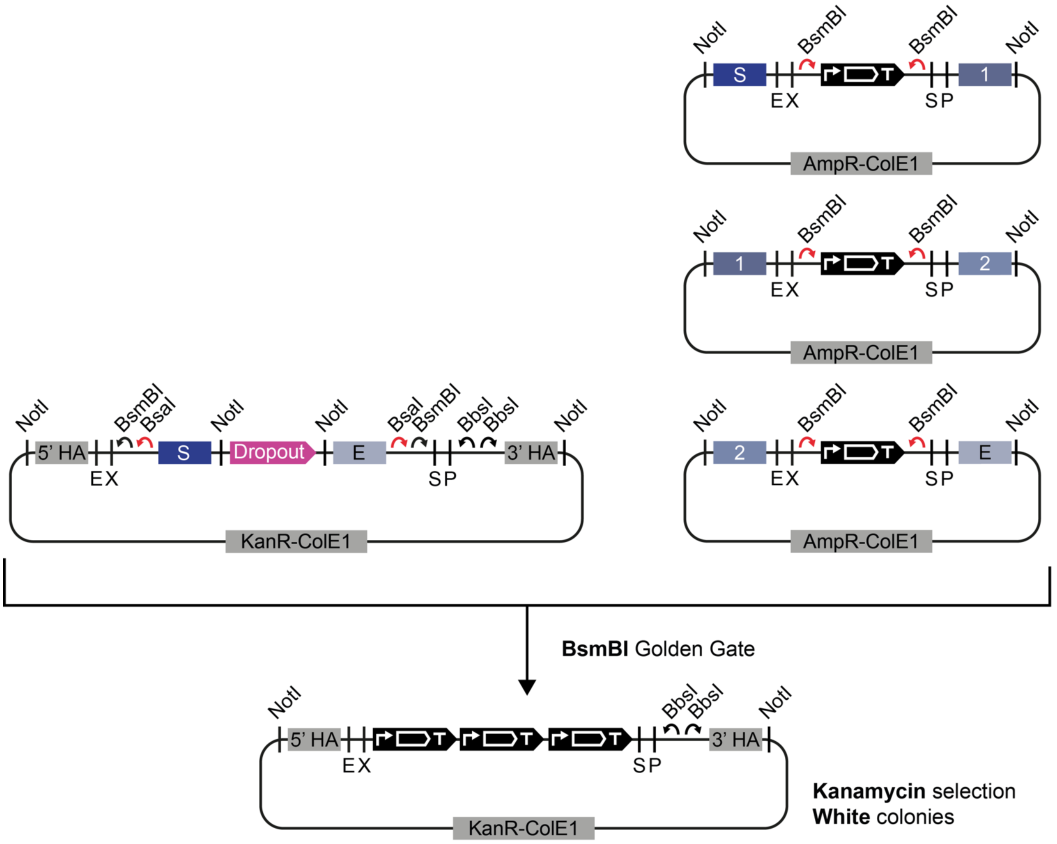

Assembly of Level 1 cassettes into a Level 2 multigene cassette. Level 1 cassettes are assembled into an integration vector using a BsmBI Golden Gate reaction, ensuring the connector sequences included in the Level 1 cassettes begin with ConS and end with ConE with matching connectors in between. See **Supporting Table 4** for an overview of the organization of Level 1 cassettes within a Level 2 multigene assembly. Completed reactions are transformed into *E. coli* and selected on kanamycin medium. Plasmids are prepped from non-fluorescent, white colonies (loss of red fluorescence) and correct assemblies are validated using a NotI digestion. Long-read sequencing can also be used for validating larger assemblies where differences in fragment size are difficult to distinguish using gel electrophoresis.

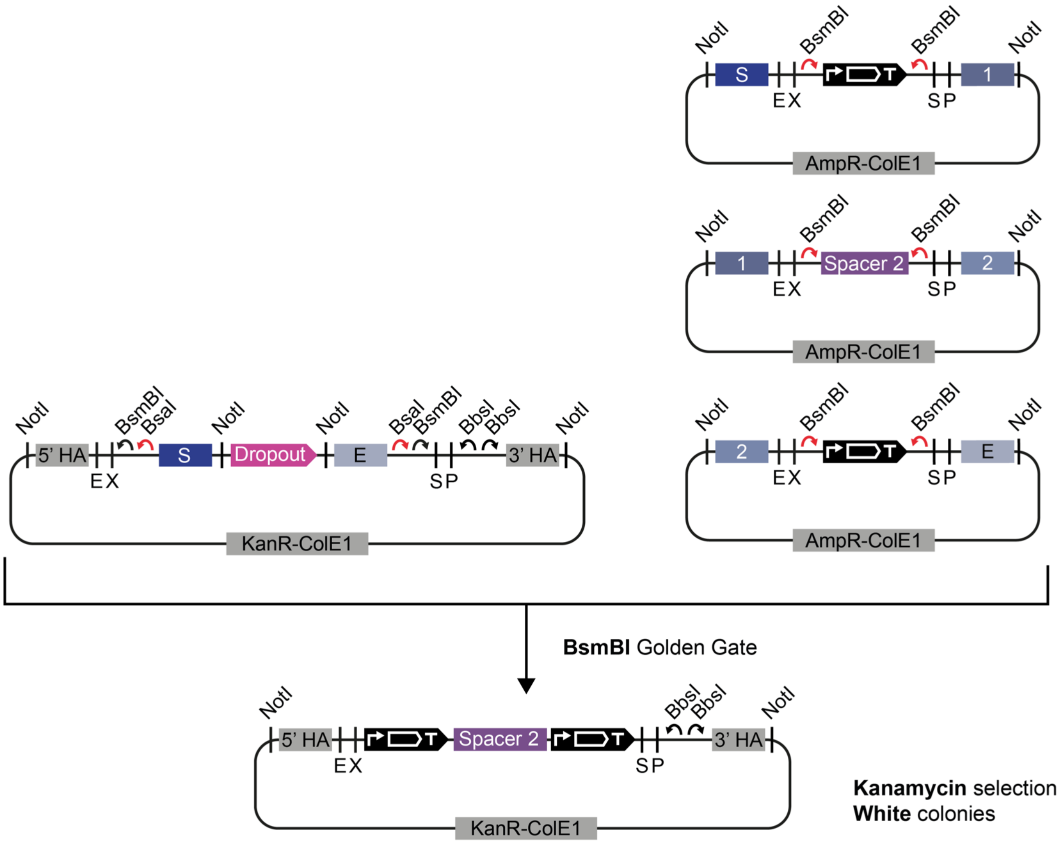

Assembly of Level 1 cassettes and spacers into a Level 2 multigene cassette. Assembly spacers (pMYT057-74) can be used to substitute for a TU to provide flexibility in the Level 2 assembly. Level 1 cassettes and assembly spacers are assembled into an integration vector using a BsmBI Golden Gate reaction, ensuring the connector sequences included in the Level 1 cassettes and assembly spacers begin with ConS and end with ConE with matching connectors in between. See **Supporting Table 4 and 5** for an overview of the organization of Level 1 cassettes and spacers within a Level 2 multigene assembly. Completed reactions are transformed into *E. coli* and selected on kanamycin medium. Plasmids are prepped from non-fluorescent, white colonies (loss of red fluorescence) and correct assemblies are validated using a NotI digestion. Long-read sequencing can also be used for validating larger assemblies where differences in fragment size are difficult to distinguish using gel electrophoresis.

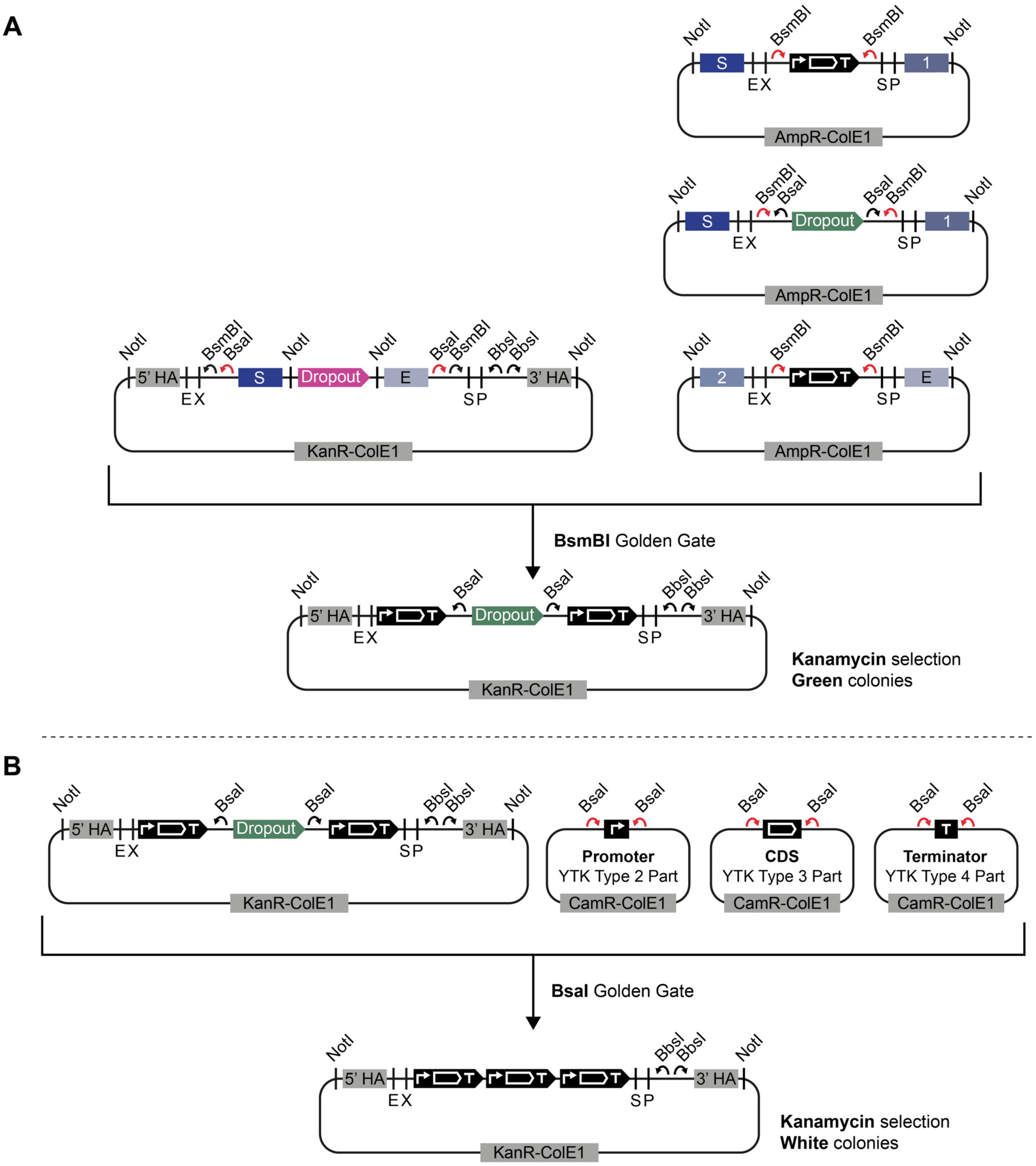

Assembly of multigene cassettes with a dropout. (A) Assembly of a Level 2 multigene cassette with a dropout for the downstream assembly of a Level 1 TU at a chosen position within a Level 2 multigene cassette. Sometimes it is desirable to assemble all but one TU into a multigene assembly, for example, when creating a common plasmid where several TUs are constant but one varies. This would allow the single TU to be directly assembled into a multigene assembly in a single step. To facilitate this, an unassembled Level 1 cassette can be used in a Level 2 multigene assembly instead of an assembled TU. Level 1 cassettes and an unassembled assembly cassette are assembled into an integration vector using a BsmBI Golden Gate reaction, ensuring the connector sequences included in the Level 1 cassettes and assembly spacer begin with ConS and end with ConE with matching connectors in between. See **Supporting Table 4** for an overview of the organization of

Level 1 cassettes within a Level 2 multigene assembly. Completed reactions are transformed into *E. coli* and selected on kanamycin medium. Plasmids are prepped from green fluorescent colonies (and loss of red fluorescence) and correct assemblies are validated using a NotI digestion. Long-read sequencing can also be used for validating larger assemblies where differences in fragment size are difficult to distinguish using gel electrophoresis. (B) Part plasmids, formatted in the YTK standard (Type 2 through Type 4), are assembled into the multigene plasmid containing a dropout in a BsaI Golden Gate reaction and transformed into *E. coli* and selected on kanamycin medium. Plasmids are prepped from non-fluorescent, white colonies (loss of green fluorescence) and correct assemblies are validated using a NotI digestion. Long-read sequencing can also be used for validating larger assemblies where differences in fragment size are difficult to distinguish using gel electrophoresis.

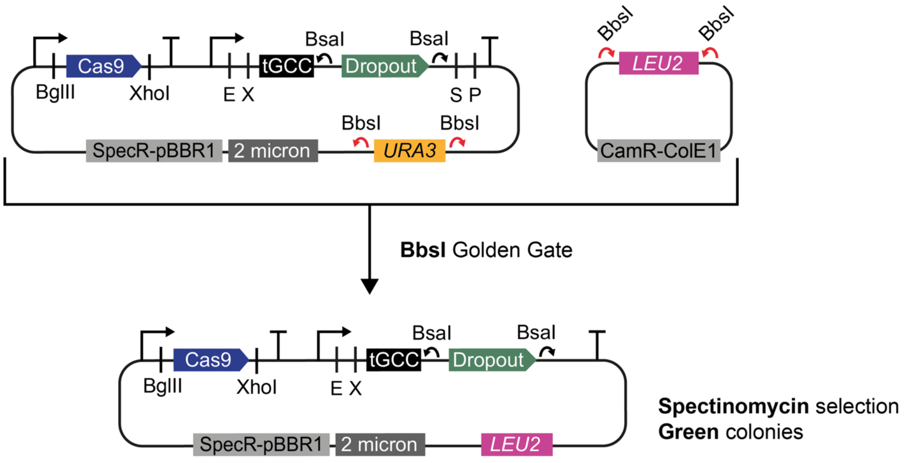

Assembly of a selectable marker into the pMYT095 CRISPR-Cas9 plasmid to replace the *URA3* marker. Selectable markers (pMYT029-038) are cloned into pMYT095 vectors using a BbsI Golden Gate reaction and transformed into *E. coli* and selected on spectinomycin medium. As the assembly does not change the fluorescence marker for screening successful assembly, we recommend adding an excess of the selectable marker plasmid to drive plasmid assembly to completion. Using 12.5 fmol of plasmid backbone and 50 fmol of the insert works well for us in a standard Golden Gate reaction and usually leads to all resulting clones being correct. All colonies should have green fluorescence. Use BsaI digestion to validate assembly. Selectable markers can be cloned into CRISPR-Cas9 plasmids where arrays have already been assembled, however, BbsI sites cannot be present in the gRNA array.

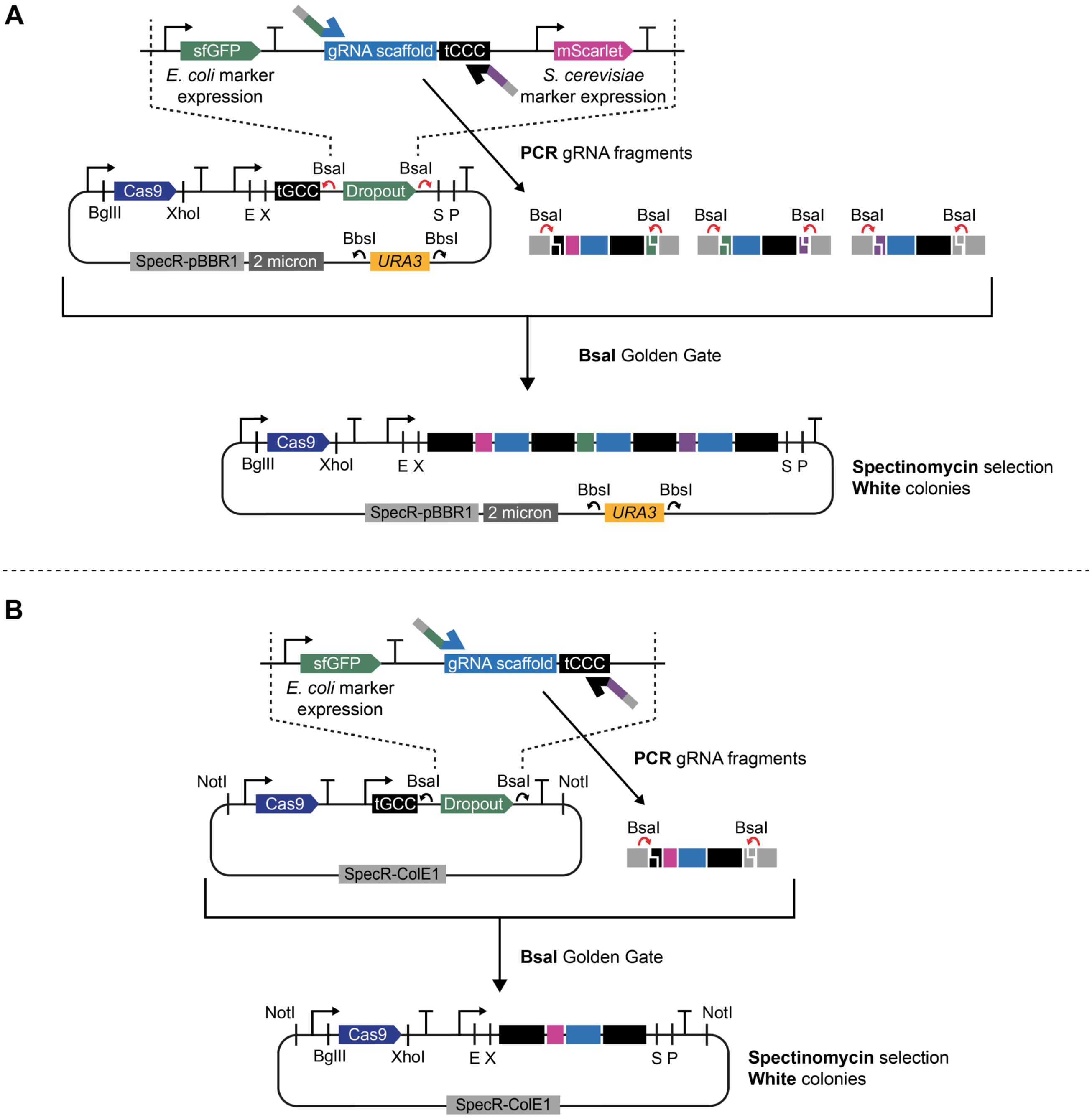

Assembly of gRNA-tRNA arrays. (A) Generation of gRNA-tRNA fragments and assembly of arrays into pMYT095. To create the gRNA-tRNA fragments for array assembly, the template for PCR is located within the dropout of pMYT095, which consists of the Cas9 handle followed by a tRNA (tCCC). Primers are designed to amplify the template and include the spacer and cloning sequences in the overhang, so that upon assembly the 20 bp spacer is prepended to the Cas9 handle (creating the gRNA), which is directly followed by a tRNA. As the plasmid backbone contains a tRNA (tGCC) at the start of the array, each gRNA is flanked by a tRNA on both sides once assembled. See **Supporting Table 3** for primer design. The purified fragments are then assembled into pMYT095 in a BsaI Golden Gate reaction and transformed into *E. coli* and selected on spectinomycin medium. Plasmids are prepped from non-fluorescent, white colonies (loss of green fluorescence) and correct assemblies are validated using Sanger sequencing. See **Supporting Table 9** for gRNA array

sequencing primers. Long-read sequencing can also be used for validating larger arrays where Sanger sequencing from both ends in insufficient. An mScarlet expression cassette was also included in the dropout for screening gRNA array assembly in yeast. Following the Golden Gate reaction, assemblies can be directly transformed into yeast, alongside the linearized integration vectors (or other donor DNA) to bypass plasmid isolation and sequencing, saving time and effort. Yeast colonies can then be screened for the loss of red fluorescence. However, we did not characterize this method, and we expect to see a large decrease in CRISPR-Cas9 editing efficiency, as seen in the Lightning GTR-CRISPR method reported by Zhang et al. (2019). For experiments with low multiplexing requirements, this may be a worthwhile trade-off. For experiments with greater multiplexing requirements, we recommend first assembling and validating the gRNA-tRNA array before transforming into yeast. (B) Assembly of an individual gRNA-tRNA fragment into pMYT096. We recommend only assembling a single gRNA into the transient CRISPR-Cas9 plasmid pMYT096, as multiplexed targeting can instead be achieved by combining transient CRISPR-Cas9 plasmids targeting different loci. This simplifies plasmid creation and allows greater flexibility downstream. gRNA arrays are generated using the same method as for pMYT095.

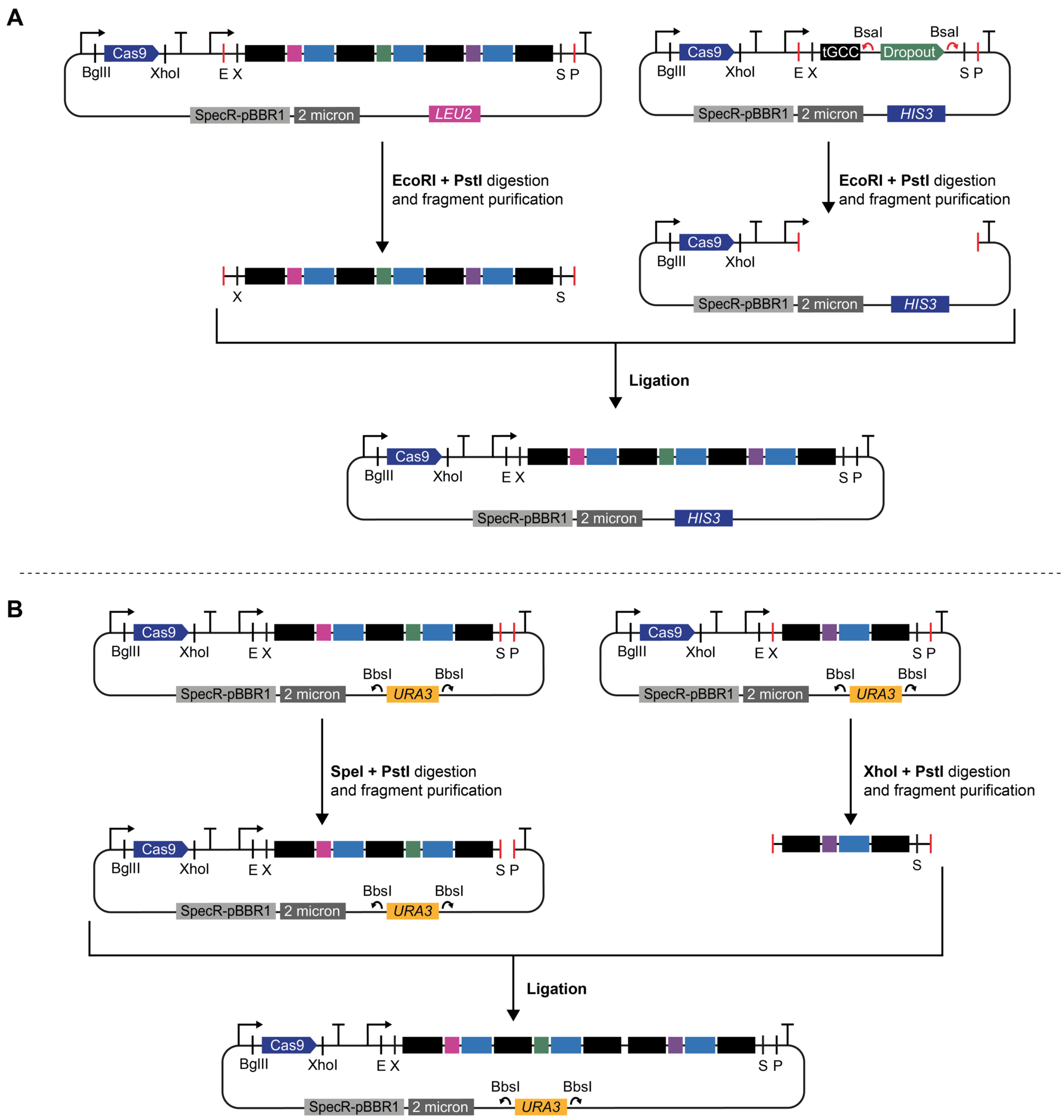

Manipulation of assembled gRNA arrays using restriction cloning. (A) Transposition of gRNA arrays into a new CRISPR-Cas9 plasmid with an alternative marker. If an array has been assembled into a CRISPR-Cas9 plasmid where the selection marker has already been terminally swapped out and a different marker is required, rather than recreating the array, the array can be transposed to a different CRISPR-Cas9 plasmid using an EcoRI and PstI digestion/ligation. (B) BioBrick assembly of gRNA arrays. To allow the combination of previously assembled arrays without the need to design the array, arrays can be merged using BioBrick assembly. Upon completion the restriction enzyme sites are restored, and additional arrays can be added.

Multiplex Yeast Toolkit plasmid genome integration methods

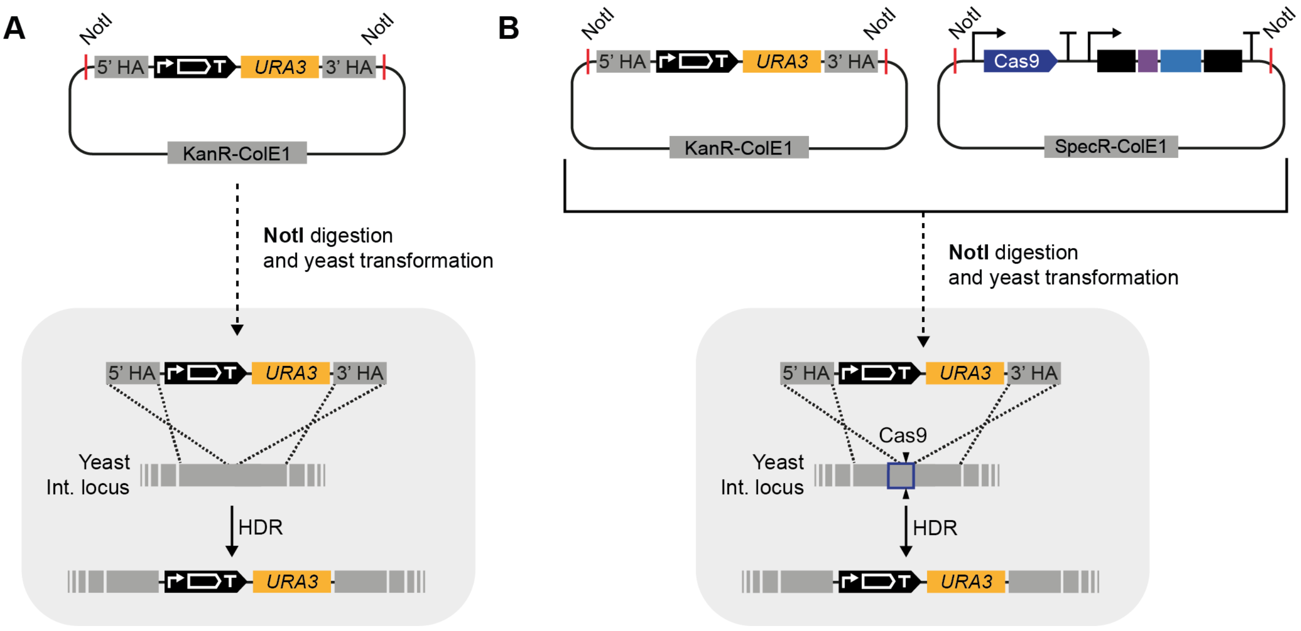

Integration of a single selectable vector into the yeast genome. (A) Integration of a single selectable integration vector into the yeast genome without CRISPR-Cas9. The plasmid is digested using NotI, heat inactivated, transformed directly into yeast without a clean-up step, and plated on the appropriate selection medium. (B) Integration of a single selectable integration vector into the yeast genome using transient CRISPR-Cas9 to boost integration efficiencies. The integration vector and transient CRISPR-Cas9 plasmid targeting the cognate locus are co-digested using NotI, heat inactivated, transformed directly into yeast without a clean-up step, and plated on the appropriate selection medium. Note: this is a very high efficiency method for a single integration vector and will lead to >10^5 colonies. This can be useful for creating large libraries of yeast, however, for routine integration of single vectors, we recommend not using the transient CRISPR-Cas9 method as dilutions of the resuspended transformants are required.

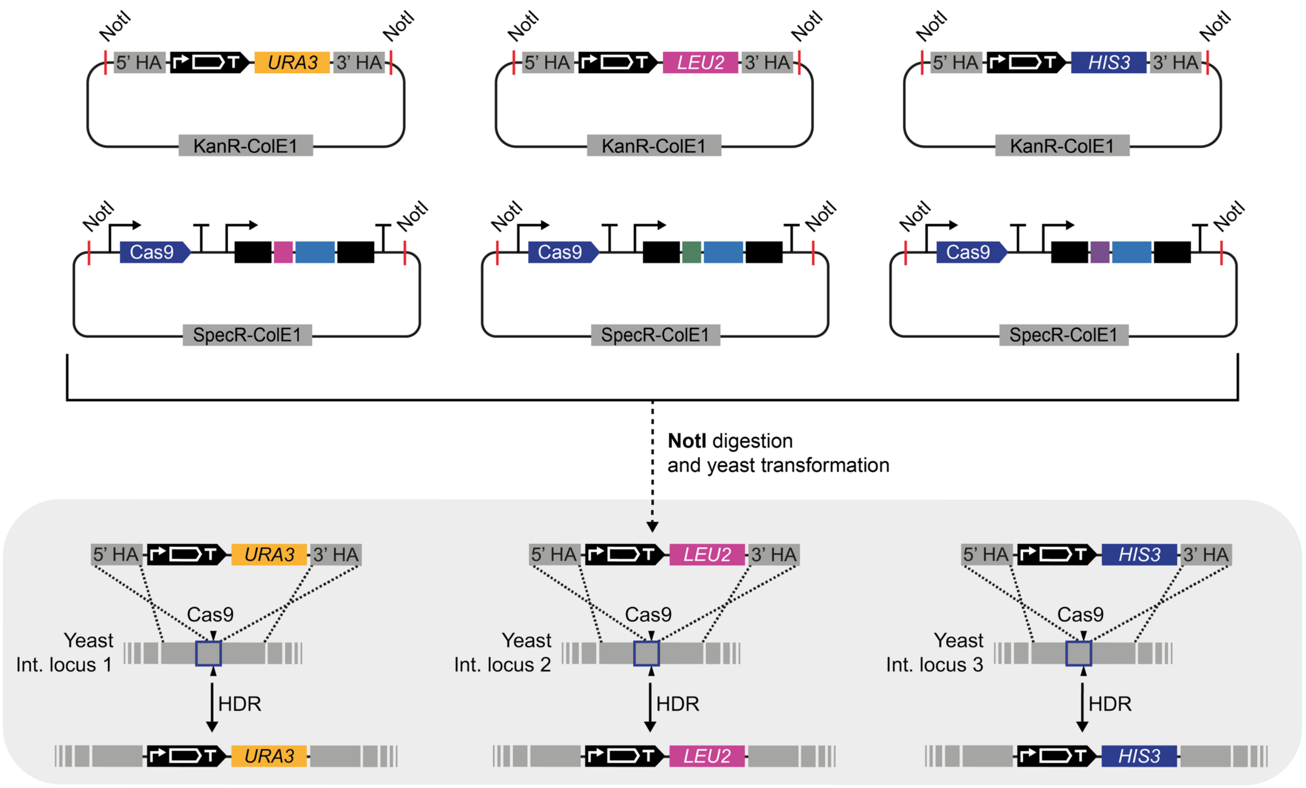

Multiplexed integration of selectable integration vectors into the yeast genome using transient CRISPR-Cas9. The integration vectors and transient CRISPR-Cas9 plasmids targeting the cognate loci are co-digested using NotI, heat inactivated, transformed directly into yeast without a clean-up step, and plated on medium lacking/containing the appropriate nutrients/antibiotics. We highly recommend this approach for routine work as the efficiency of multiplexed integration is very high, plasmids can be combined with very low effort, small amounts of each plasmid are required and do not require a clean-up following the co-digestion, and the selectable marker avoids the necessary genotyping of markerless integration.

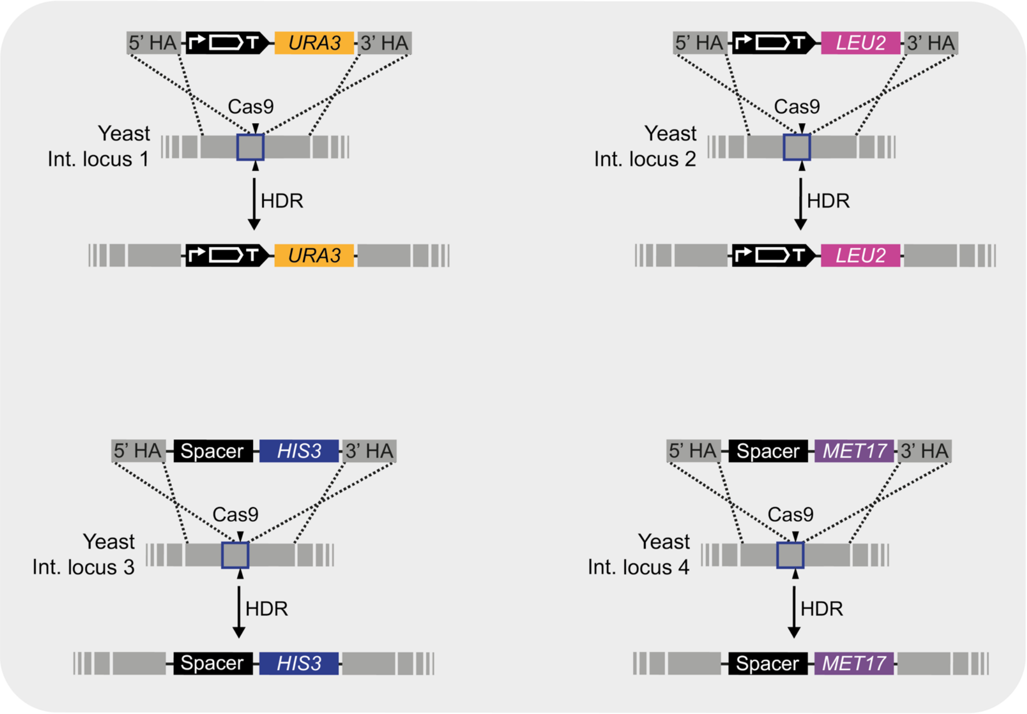

Complementation of yeast auxotrophies in a single transformation. One beneficial use for the multiplexed integration of multiple selectable vectors/spacers using transient CRISPR-Cas9 is to restore all auxotrophies of a yeast strain in a single transformation. If any remaining auxotrophic markers for a particular strain are not included on the integration vectors, these can instead be introduced using the integration spacers. This method is useful for restoring unused background auxotrophies to allow full flexibility of the growth medium. As the markers are integrated, they are single copy and will remain stable through all subsequent experiments. We also highly recommend this approach when consistency between strains is required, such as experiments where comparative growth is important. If restoring all auxotrophies is not important, or they need to be kept open for downstream purposes, we still recommend keeping the chosen markers consistent between strains, using the integration spacers where necessary. Growth is paramount to most applications, and this additionally simplifies plating, as a single dropout medium can be used across all transformants. This approach is performed as outlined in the previous figure.

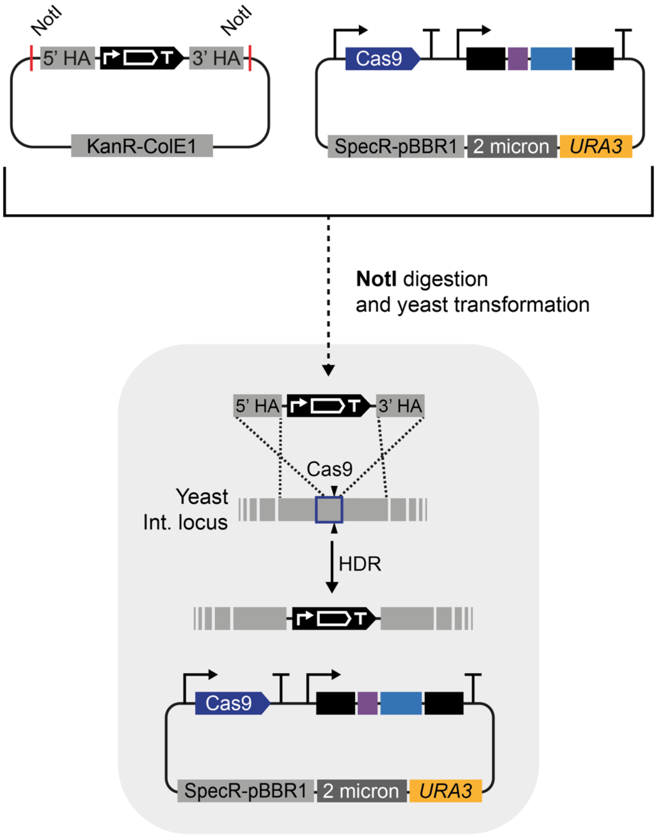

Markerless integration of a vector into the yeast genome using CRISPR-Cas9. The markerless integration vector is combined with the CRISPR-Cas9 plasmid which targets the cognate locus and digested using NotI. As the CRISPR-Cas9 plasmid does not contain a NotI site, only the integration vector is linearized. After heat inactivation, the entire reaction is transformed into yeast without a clean-up step and plated on the appropriate selection medium. Correct integration should be validated using PCR genotyping. See **Supporting Table 10** for a list of colony PCR primers.

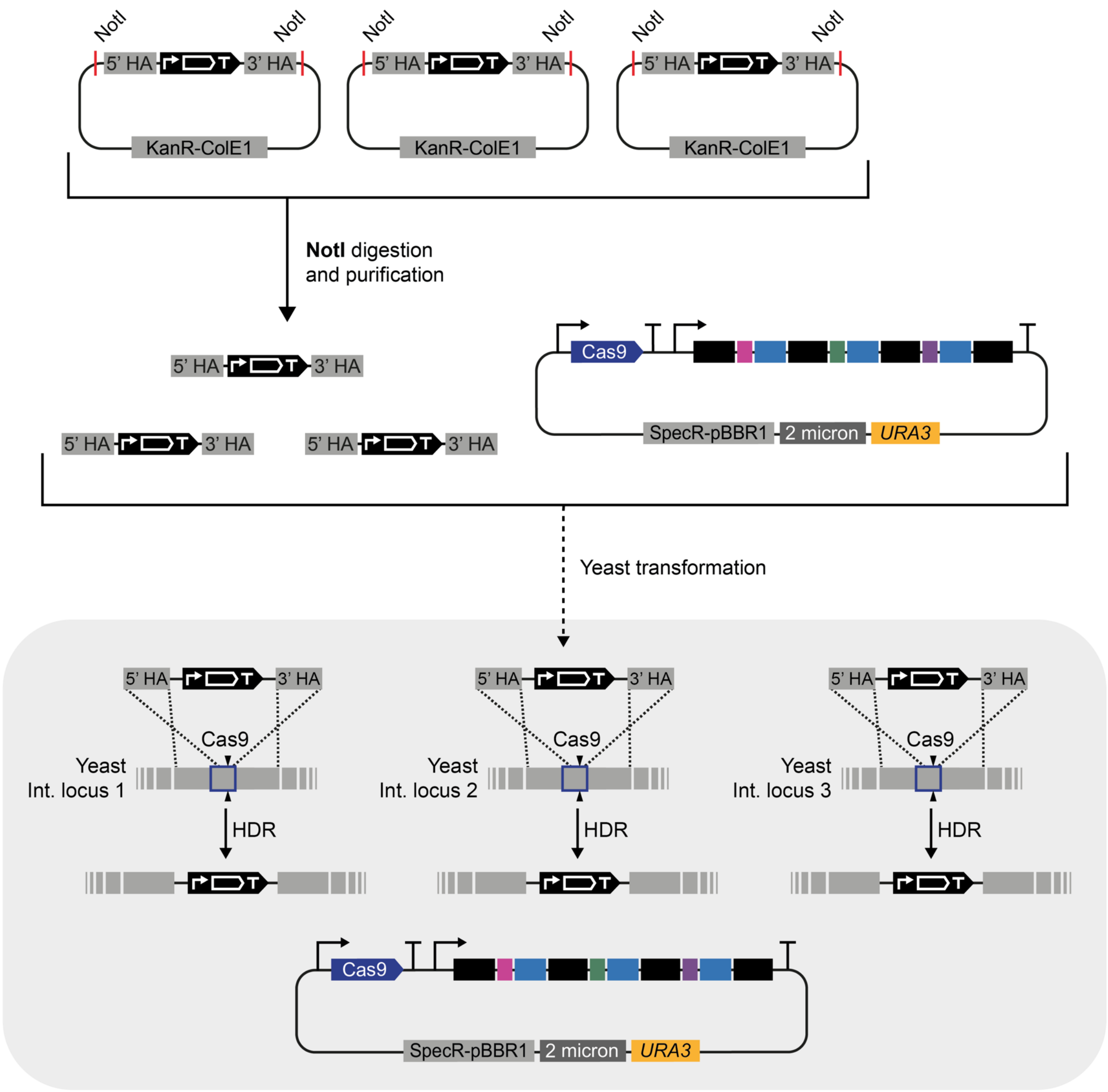

Multiplexed markerless integration of vectors into the yeast genome using CRISPR-Cas9. For multiplexed markerless integration, the integration vectors should be individually digested using NotI and cleaned up using a PCR column purification. Following DNA purification, the linearized integration vectors are then combined with the CRISPR-Cas9 plasmid, transformed into yeast, and plated on the appropriate selection medium. Correct integration should be validated using PCR genotyping. See **Supporting Table 10** for a list of colony PCR primers. As large quantities of DNA are required when multiplexing the integration of multiple integration vectors, the clean-up step serves to concentrate the final DNA used for transformation (which can be eluted in lower volumes) and remove the digestion buffer, which decreases the markerless integration efficiency. However, when integrating only a few integration vectors (≤4), this can likely be skipped, while maintaining high efficiencies. In this case, the integration vectors can be co-digested and directly transformed into yeast alongside the CRISPR-Cas9 plasmid. We recommend only using this approach if the NotI digestion can be performed in low volumes (e.g. < 20 μL), so large volumes of digestion buffer are not present in the yeast transformation.

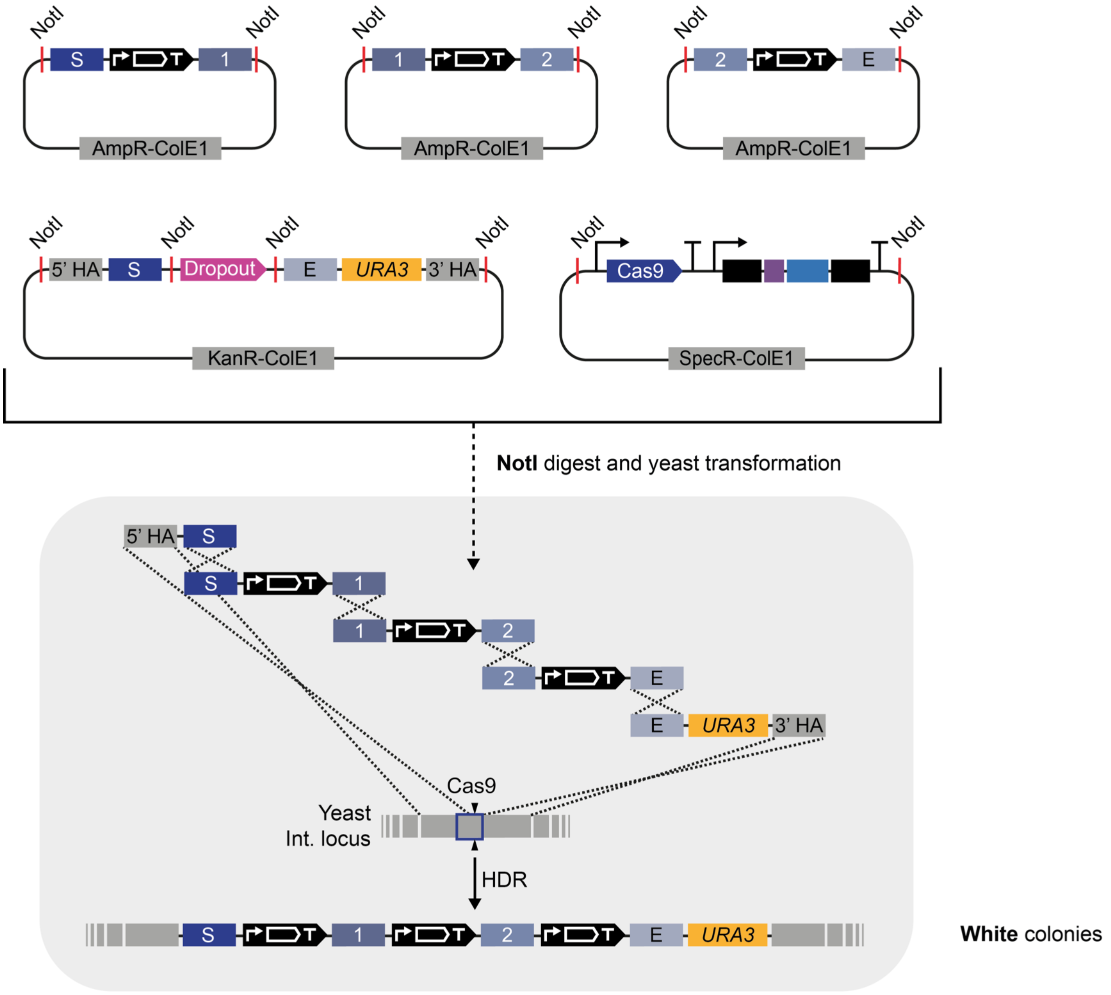

Gap repair assembly of Level 2 multigene cassettes into the yeast genome from Level 1 cassettes. The integration vector, Level 1 cassettes, and a transient CRISPR-Cas9 plasmid targeting the appropriate locus are digested in a one-pot reaction with NotI, heat inactivated, transformed directly into yeast without a clean-up step, and plated on the appropriate selection medium. Non-fluorescent, white yeast colonies are then chosen based on the loss of red fluorescence from the mScarlet dropout marker. Gap repair in yeast assembles the final multigene construct at the genomic locus according to the homology between the integration vector and cassettes, with the transient CRISPR-Cas9 increasing the overall efficiency of the process. As with Level 2 Golden Gate assemblies, the connector sequences included in the Level 1 cassettes need to begin with ConS and end with ConE, with matching connectors in between. This gap repair method can only be used once per strain, as reused connector sequences will lead to stability issues between loci. However, this method can be useful for rapidly assembling large multigene constructs in yeast which can be difficult to clone in vitro. This method is also amenable to a combinatorial approach by including multiple alternative cassettes at the same position in the multigene assembly. In our experience, this gap repair method has very high fidelity when all cassettes are transformed at equimolar concentrations (described in **Supporting Table 6**), and we did not encounter any misassembled multigene constructs in this work. Nevertheless, barcodes are included inside of each connector sequence for validating the gap repair junctions if desired (**Supporting Table 11**). It is essential that no repeated DNA sequences (promoters, CDSs, terminators) are used between Level 1 cassettes, as these may recombine during transformation and lead to incorrect assembly and loss of the TUs.
